# A network analysis of female sexual function: comparing symptom networks in women with decreased, increased, and stable sexual desire

**DOI:** 10.1038/s41598-018-34138-8

**Published:** 2018-10-25

**Authors:** Annika Gunst, Marlene Werner, Lourens J. Waldorp, Ellen T. M. Laan, Marianne Källström, Patrick Jern

**Affiliations:** 1 University of Turku, Department of Psychology, Turku, 20014 Finland; 2 University of Amsterdam, Department of Psychology, Amsterdam, 1018 The Netherlands; 3 University of Amsterdam, Academic Medical Center, Department of Sexology and Psychosomatic Obstetrics and Gynaecology, Amsterdam, 1105 The Netherlands; 4 Åbo Akademi University, Department of Psychology, Turku, 20500 Finland

## Abstract

Problems related to low sexual desire in women are common clinical complaints, and the aetiology is poorly understood. We investigated predictors of change in levels of sexual desire using a novel network approach, which assumes that mental disorders arise from direct interactions between symptoms. Using population-based data from 1,449 Finnish women, we compared between-subject networks of women whose sexual desire decreased, increased, or remained stable over time. Networks were estimated and analyzed at T1 (2006) and replicated at T2 (2013) using R. Domains included were, among others, sexual functions, sexual distress, anxiety, depression, body dissatisfaction, and relationship status. Overall, networks were fairly similar across groups. Sexual arousal, satisfaction, and relationship status were the most central variables, implying that they might play prominent roles in female sexual function; sexual distress mediated between general distress and sexual function; and sexual desire and arousal showed different patterns of relationships, suggesting that they represent unique sexual function aspects. Potential group-differences suggested that sex-related pain and body dissatisfaction might play roles in precipitating decreases of sexual desire. The general network structure and similarities between groups replicated well; however, the potential group-differences did not replicate. Our study sets the stage for future clinical and longitudinal network modelling of female sexual function.

## Introduction

Problems related to low sexual desire are common in women, with approximately one in four adult premenopausal women reporting low sexual desire^1^. Low sexual desire has been associated with various negative personal and interpersonal factors such as decreases in general health, personal wellbeing, and relationship satisfaction^2–4^. It is also the most common sexual complaint among women in sex therapy^5^. However, despite the high prevalence of low sexual desire and its negative associated factors, the aetiology of low sexual desire is still poorly understood. In the present study, we investigate whether a novel network approach might shed further light on the aetiology of low sexual desire in women.

The aetiology of low sexual desire in women is commonly referred to as a multifactorial phenomenon, with studies showing that it is associated with various inter- and intra-personal factors such as relationship status and partner compatibility^6,7^, having children^3^, body image^8^, physical health^9^, mental health^10^, alcohol consumption^2^, and sexual attitudes^11^. However, attempts to identify causal paths between these factors through longitudinal study have been largely disappointing^7,12^, and the nature, aetiology, and progression of low sexual desire is, to a large extent, still unknown.

In recent years, a network perspective of mental disorders has gained popularity in psychopathology research^13,14^. The network theory of psychopathology postulates that mental disorders arise from direct interactions between symptoms. For instance, major depression could be regarded as the final state in a cascade of symptoms affecting each other. After experiencing a stressful life event, one could have trouble sleeping, which could lead to difficulties concentrating, which could lead to feelings of worthlessness and sadness, which could exacerbate sleeping problems. In such a scenario, the symptoms act on each other in negative feedback loops and individuals might enter a pathological depressive state in which connected symptoms continue to activate each other^15^. In this way, the network perspective differs from the biomedical model wherein observed symptoms are assumed to have one, purely biological, latent aetiology and result from one shared causal factor^16^.

According to the network theory of psychopathology, individuals might differ in terms of how vulnerable/resilient they are to developing symptoms of a disorder or disease because of differences in how strongly symptoms are connected^17^. Individuals with stronger network connections between symptoms are more vulnerable to developing psychopathology: for some people, even one night of poor sleep could have severe consequences for the ability to concentrate the next day (i.e., strong connections between symptoms) whereas for other people, one night of poor sleep might not affect the ability to concentrate to any noteworthy extent (i.e., weak connections between symptoms).

One advantage of the network approach is that it can offer clinically insightful observations. For instance, it emphasises individual symptoms and their role in disorder progression, which is in line with novel approaches in medicine^18^. Furthermore, the symptom approach resembles how clinicians already conceptualise and diagnose mental disorders^19^. The framework can also help to explain common clinical phenomena: for instance, comorbidity is explained as a natural feature of psychopathology due to symptoms being shared between different disorders, which can act as bridges between disorders^20^. Such a conceptualization of comorbidity might be insightful for the aetiology of sexual dysfunctions, as comorbidity between diagnostically different sexual dysfunctions such as low sexual desire, orgasm problems, and sex-related pain is common^21^.

**Figure 1:**
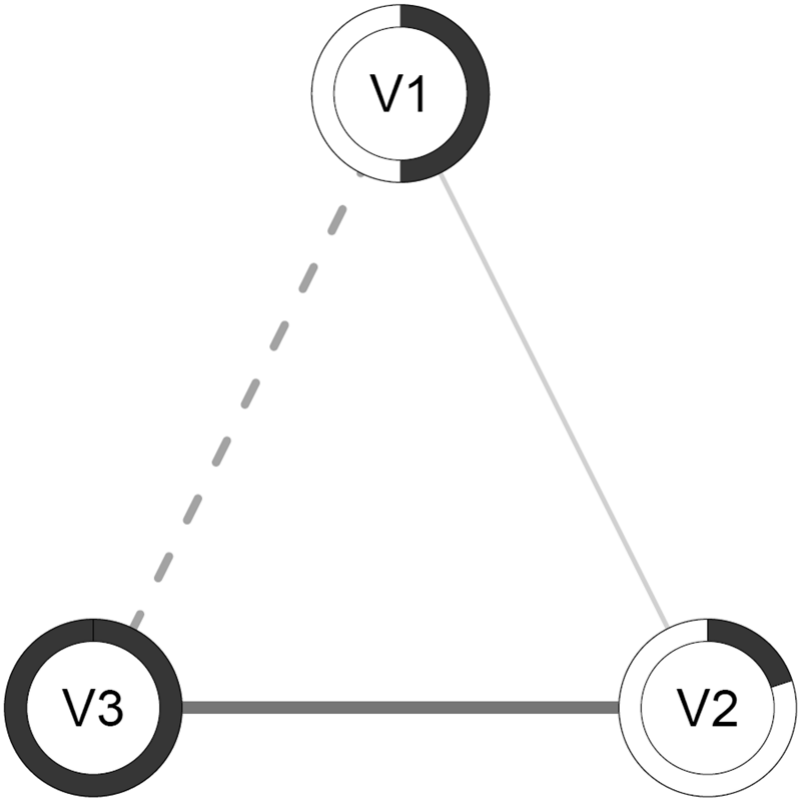
Illustrative example of a network model with three variables. In a network, variables are represented by “nodes” (illustrated as circles), and relationships between variables are represented by “edges” (illustrated as lines between nodes). Edges represent (regularized) partial relationships. Dashed edges represent negative relationships and solid edges represent positive relationships. The thicker and more saturated the edge, the stronger the relationship. The pies surrounding the nodes represent the amount of explained variance of the variable by the variables connected to it. The fuller the pie (darker), the more variance explained.

Recently, software has been developed for the estimation of psychological (symptom based) network models^22,23^, and the methodological quality of this novel approach has been ascertained in simulation studies^22,24,25^. Furthermore, network theory has already been applied successfully to empirical data on various concepts such as depression^15,26,27^, post-traumatic stress^28^, psychotic disorders^29^, and substance abuse^30^. Network models can be estimated and visualized with statistical software (e.g., *qgraph* package^27^). See Fig. 1 for an example of a visualized illustrative network model and a description of the visualized elements.

In the present study we aimed to explore the network structure of symptoms of female sexual (dys)function and relevant correlates using population-based data collected at two time points seven years apart (T1 and T2). We compared the network structures of three groups at T1: women whose desire *decreased* between T1 and T2, women whose desire *increased* between T1 and T2, and women whose desire remained relatively *stable* between T1 and T2. By comparing these groups, we wanted to explore network characteristics that are associated with changes in sexual desire. In line with network theory, we hypothesised that women whose desire decreased between the two time points would show stronger positive network connectivity among “negatively” valenced variables (so that variables such as, e.g., sexual distress and sex-related pain would show a stronger connection between each other) at T1, whereas women whose desire increased between the two time points would show stronger positive connections among “positively” valenced variables at T1 (e.g., sexual satisfaction and orgasm function). Similarly, we additionally hypothesised that women with decreases in desire would show stronger negative connectivity between “negatively” and “positively” valenced variables (e.g., sexual distress and orgasm function) compared to the other groups. These hypotheses reflect the idea that stronger connections between symptoms could be expected to lead to more changes.

## Methods

### Participants

The total sample used in the analyses included 1,449 women who had participated in two waves of a Finnish population-based study: the Genetics of Sexuality and Aggression study conducted in 2006 (T1; mean age 25.5 years, *SD* = 4.9) and 2013 (T2). The data collection procedure is described at length elsewhere^7,31^. To correct for familial dependency, due to the participants being twins and sisters of twins and thus genetically related, one person per family was randomly selected from the original 2,173 women who had submitted data at both time points, resulting in a sample of 1,729 women. Since network estimation needs full information, missing values were imputed for quantitative variables (described further in the *Statistical Analyses* section). Thirty-seven participants that had missing data on the categorical variable relationship status at T1 were excluded from the analyses. Furthermore, when comparing networks between groups, it is important that the sample size for each group is the same^32^. As the smallest subgroup consisted of 483 participants (the three subgroups were *decreased*, *increased*, and *stable* desire; the grouping procedure is described further in the *Statistical Analysis* section), we randomly removed participants from the two bigger subgroups in order to equalize the subgroup sizes to 483. This resulted in the final total sample of 1,449 women.

Both data collections were approved by the Ethics Committee of Åbo Akademi University, and the study was carried out in accordance with the Helsinki Declaration. Written informed consent was obtained from all participants at both time points. Data can be made available upon request to the corresponding author.

### Measures

A short form of the revised Sexual Desire Inventory^33^ (SDI-2) was used for creating the grouping variable assessing change in sexual desire over time. The following measures were included in the network: The Female Sexual Function Index^34^ (FSFI), assessing six domains of sexual functioning: *sexual desire*, *sexual arousal*, *lubrication*, *orgasm function*, *sexual satisfaction*, and *sex-related pain*; a short form of the Female Sexual Distress Scale^35^, assessing *sexual distress*; two subscales of the Brief Symptom Inventory-18^36^, assessing *depression* and *anxiety*; the body image subscale of the Derogatis Sexual Function Inventory^37^, assessing *body dissatisfaction*; the Alcohol Use Disorders Identification Test^38^, assessing *alcohol use*; the Desired and Actual Sexual Activity Scale^39^, assessing discrepancy in desired and actual frequency of sexual behaviours (*too much sexual activity* and *too little sexual activity*); and the Sociosexual Orientation Inventory^40^, assessing *sociosexual orientation*. Detailed information about the measures and psychometric properties is provided as supplementary material. Other measures included in the networks were *age*, *height*, *weight*, use of *hormonal contraception* (yes/no), *number of* (biological) *children*, and a question inquiring about whether the woman was in a steady sexual relationship (*relationship status*) at the time of study (yes/no).

### Statistical Analyses

#### Handling of missing data

At T1, 4.1% of the data were missing in the included variables, and at T2, 0.2% of the data were missing. We imputed missing data for quantitative variables with the SPSS Missing Value Analysis regression method, using residual adjustment. We used all quantitative study variables as predictors for T1 and T2, respectively.

#### Sexual desire groups

We based the grouping on the SDI-2 and not the FSFI desire subscale, as grouping based on a measure that is included in the network can lead to induced problematic artefacts within the covariance structure within the subgroups^41^. Individual change scores for the SDI-2 were first computed by subtracting the T1 score from the T2 score. Then, the women were divided into three groups based on the change score: those with (a) *decreased* levels of sexual desire (women with scores more than −0.5 standard deviations from the mean of the change score), (b) *increased* levels of sexual desire (women with scores more than +0.5 standard deviations from the mean), and (c) a relatively *stable* level of sexual desire (women with scores within 0.5 standard deviations from the mean). Note that variables included in the estimated networks were, in contrast to the grouping variable, based on only one time point (T1 and T2, respectively). Before the network estimations, we assured that our grouping variable did not correlate too strongly with any variables included in the estimated networks (see supplementary material), making it unlikely that the grouping procedure affected the covariance structure and induced relationships in the networks by conditioning on a common effect^42^.

#### Network analyses

We analysed the data both at T1 and T2, using network packages for *R* (version 3.3.3). In the analyses for T2, we aimed to replicate the analyses of T1. Each analysis was run for each desire group separately.

Network estimation and interpretation: First, we estimated the network models of the three groups and compared the network structure visually and by comparing differences in edge significance and explained variance of variables. We further checked whether edge estimates were stable across bootstrapped estimations based on subsamples of the data.

We estimated the network models with the *mgm* package (i.e., our networks are mixed graphical models, MGMs^23^, which model relationships according to the distributional assumptions of the respective variables; continuous, ordinal, or categorical). In our MGMs, all relationships represent pairwise interactions (i.e., *k* = 2, interactions). Furthermore, in all estimations of relationships between two variables, their relationships with all other variables included in the network are controlled for. Thus, the absence of a relationship between two variables indicates that those two variables are conditionally independent given all other variables. In order to minimize the number of estimated parameters and limit the likelihood of estimating false positives, we used regularization and EBIC model selection (i.e., the software estimates several models with differing levels of sparsity and chooses the best fitting model according to the Extended Bayesian Information Criterion, which is similar to the Bayesian Information Criterion but with an additional term that takes into account the size of all possible models), and set the hyperparameter to γ = 0.5 as suggested in the literature^43^. Further technical details about MGMs can be found elsewhere^23^.

We visualized the estimated network models using the *qgraph* package^44^. The network layout was determined with the Fruchterman-Reingold algorithm for each network separately, so that strongly connected nodes attract each other whereas disconnected nodes repulse each other^44^. However, to make the networks visually comparable we used the average layout across the three individual layouts when plotting the network models.

In the interpretation of relationships and respective differences between groups, we also used edge stability and significance plots (*bootnet* package^22^). Edge stability plots indicate how accurately we can estimate edges as well as how stable the order of relative edge magnitudes is based on the data at hand. In the edge stability plot, we visualized 95% bootstrapped confidence intervals based on re-estimations of edges on resampled observations in the data (with replacement). Note that edge significance plots do indicate which edges are significantly different from each other in magnitude (*α* = 0.05), but not whether edges are significantly different from 0.

We also calculated how much of a variable’s variance can be explained by variables connected to it in the network (i.e., nodewise predictability^45^). High predictability of a variable indicates that most of the variance of that variable can be predicted by the variables it is directly connected to.

Network connectivity comparison: Second, we compared the connectivity of the three group networks formally, using the three tests included in the *NetworkComparisonTest*^46^ (NCT): the test for differences in global strength (i.e., do the networks differ in overall strength of all relationships?), structure invariance (i.e., are there any significant differences in the nature and strength of individual edges?), and, in case the networks were structurally variant, the test for differences of each edge (i.e., which individual edges show significant differences across groups?). We should note that the NCT does not estimate relationships among variables exactly like the MGM. However, the NCT is the only statistical network comparison method currently available.

Node centrality: Third, we explored whether there were differences in the centrality of variables (i.e., nodes) between the three groups. We planned to retrieve the three most popular centrality statistics: betweenness, closeness, and strength centrality. However, since bootstrapped stability analyses indicated that the closeness and betweenness estimates were too instable across subsamples of the data (see supplementary material), we limited the interpretation of centrality statistics to strength centrality (i.e., the number and strength of a node’s direct relationship with other nodes). In the interpretation of centrality, we also used bootstrapped strength centrality statistics and strength centrality significance tests (i.e., we checked whether nodes were central across subsamples of the data and whether they were significantly more central than other nodes, which is the case when a node is more central than other nodes in 95% of bootstrapped subsamples).

Network clusters: Lastly, we ran a cluster detection algorithm to explore differences in the structure of connectivity across the three groups from an additional perspective. Clusters of nodes represent more strongly connected subnetworks in the larger network. A cluster can pinpoint a group of nodes that were to be affected most quickly when a node included in the respective subnetwork changes states. We used the *walktrap* algorithm, which identifies clusters of nodes through random walks across the network connections (*igraph* package^47^). We ran several estimations with increasing numbers of steps and chose the number of steps which resulted in the first stable number of clusters (see supplementary material for further detail).

## Results

### Network Estimation and Interpretation

**Figure 2:**
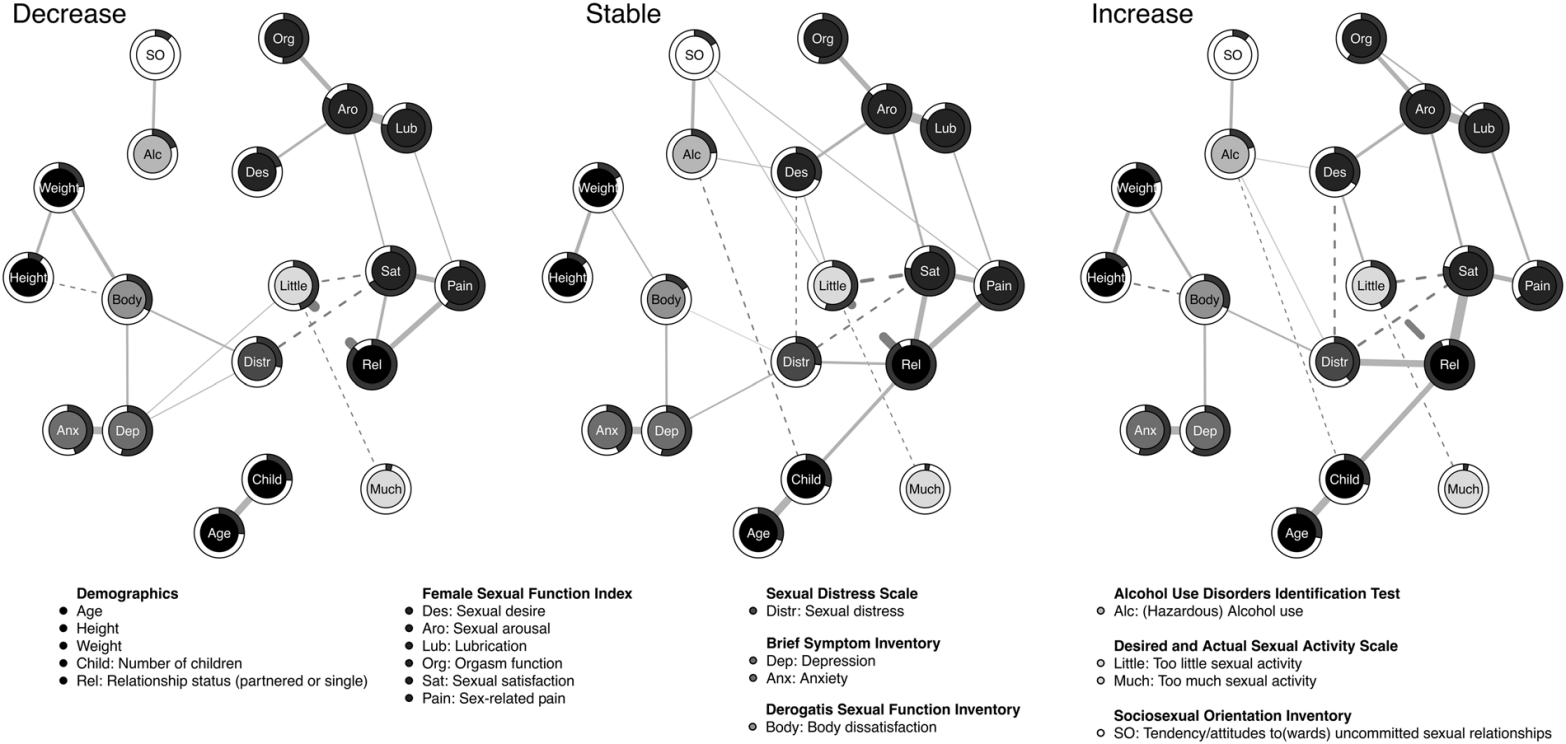
Mixed graphical model (MGM) networks for all three desire groups at the first time point. Decrease = women whose desire decreased between the two time points (2006 and 2013), Stable = women whose desire remained stable between the two time points, Increase = women whose desire increased between the two time points. Solid gray edges indicate positive, and dashed gray edges indicate negative relationships. Edges between the categorical variable relationship status and other variables can be interpreted such that, for instance, the positive edge between relationship status and sexual satisfaction implies that participants in relationships reported higher levels of satisfaction than single participants. Predictability of a variable is represented by a pie chart around the respective node; a fully filled pie chart (dark) indicates perfect predictability, an empty pie chart indicates that predictors of the variable are missing in the network model. Networks are layouted according to the averaged Fruchtermann-Rheingold layouts of all three networks; we chose a cut-off of 0.1 which controls the threshold above which edges become more saturated and a maximum of 1 which controls the maximum saturation and width of edges making the edges comparable across groups.

Descriptive statistics of all included variables can be found in Table 1. Figure 2 illustrates the network models including predictability estimates for all three desire groups based on the dataset at T1. We present network estimations which exclude *hormonal contraception* since *hormonal contraception* only related to *relationship status* in the increase group, indicating that women in relationships were more likely to use hormonal contraceptives in the increase group. *Hormonal contraception* did not relate to any other node in the increase group, and to no other node in the decrease or stable group (see supplementary material). We noted four interesting observations when comparing the networks visually.

1. First, there were fewer edges present in the network of the decrease group than in the networks of the stable and the increase group (22, 28, and 27 respectively). Specifically, the decrease group showed no direct or indirect edges between *sociosexual orientation* and the sexual function variables as well as *relationship status*, *sexual distress*, and *number of children*. Additionally, the overall network connectivity of the stable group was somewhat less strong compared to the decrease and the increase group (0.04, 0.06, and 0.08 respectively).
2. Second, we noticed many similarities in the network structure of all three groups: (a) many edges were present in all networks, (b) all edges that were present in all or two of the three networks had the same directionality and similar magnitude and (c) the pattern of predictability seemed quite comparable across networks (the latter was confirmed by looking at the values of explained variance, see supplementary material; mean explained variance was 0.41, 0.43, and 0.45 for each network respectively; *sexual arousal* was the best predicted node and *too much sexual activity* the least predicted node in all networks). Finally, (d) *sexual distress* appeared to act as a bridge between the more general distress-related nodes (*body dissatisfaction, depression, anxiety*) and sexual function in all three groups (importantly, these edges appeared to be stable, especially the *sexual distress* and *body dissatisfaction* edge in the increase and decrease group; see edge stability plot in supplementary material).
3. Third, we noted that *sexual satisfaction* and *sexual distress* showed different patterns of edges with other nodes, with *sexual satisfaction* being more closely related to sexual function indicators than *sexual distress*.
4. Fourth, we observed that the *sexual arousal* and *sexual desire* FSFI subscales showed different patterns of edges with other nodes, with *sexual arousal* being most closely related to other indicators related to sexual activity (*orgasm function* and *lubrication*) whereas *sexual desire* was related to *too little sexual activity*, *sexual distress* and *alcohol use* (at least in the stable and increase groups).

**Table 1:** Descriptive Statistics at the First Time Point (2006) for Variables Included in the Networks.

	Decrease			Stable			Increase		
	*M* (*SD*)	Mdn	Range	*M* (*SD*)	Mdn	Range	*M* (*SD*)	Mdn	Range
Age	25.67 (4.84)	25	18–43	25.18 (4.73)	25	18–43	25.54 (4.94)	25	18–44
Height	166.20 (6.44)	166	147–196.20	165.93 (6.13)	165.02	149–195	166.18 (6.18)	166	146–186
Weight	63.58 (11.33)	62	41–110	63.21 (11.11)	61	44–113	63.81 (11.77)	62	42–115
Number of children	0.50 (0.96)	0	0–6	0.52 (0.96)	0	0–6	0.57 (1.05)	0	0–9
Sexual desire	3.45 (0.87)	3.6	1.2–5.4	3.25 (0.92)	3.0	1.2–5.4	3.10 (0.92)	3	1.2–5.4
Sexual arousal	4.74 (1.56)	5.4	0–6	4.39 (1.74)	5.1	0–6	4.33 (1.82)	5.1	0–6
Lubrication	5.16 (1.63)	6	0–6	4.84 (1.88)	5.7	0–6.13	4.79 (1.98)	5.7	0–7.44
Orgasm function	4.18 (1.84)	4.8	0–6	3.85 (1.93)	4.4	0–6	3.87 (2.04)	4.8	0–6.31
Sexual satisfaction	4.20 (1.81)	4.8	0.28–6.84	4.10 (1.84)	4.8	0.4–6	4.18 (1.85)	4.8	0.4–6
Sex-related pain	4.32 (2.29)	5.4	0–6.05	4.14 (2.32)	5.4	0–6.41	4.27 (2.31)	5.4	0–6.21
Sexual distress	7.34 (5.30)	7	0–26	7.44 (5.48)	7	0–28	7.60 (5.79)	7	0–27
Depression	5.35 (4.59)	4	0–23	5.29 (4.45)	4	0–22	5.65 (4.83)	4	0–24
Anxiety	3.67 (3.71)	2	0–22	3.98 (4.15)	3	0–23	4.08 (4.24)	3	0–23
Body dissatisfaction	26.04 (6.48)	26	11–44	26.50 (6.24)	26	11–44	26.67 (6.85)	27	11–45.06
Alcohol use	7.74 (4.45)	7	1.02–26	7.16 (4.12)	6	0.93–22	6.82 (4.24)	6	0.74–31
Too little sexual activity	5.07 (4.91)	4	0–26	4.60 (5.03)	3	0–22	4.12 (4.47)	3	0–29
Too much sexual activity	0.10 (0.47)	0	0–4	0.09 (0.46)	0	0–4	0.16 (0.72)	0	0–7.63
Sociosexual orientation	68.38 (42.17)	59	7–277.86	60.81 (37.68)	56	7–335.68	60.69 (39.16)	55.75	6–249
Relationship status	*n* (%)			*n* (%)			*n* (%)		
Partner	368 (76.20)			366 (75.80)			389 (80.50)		
Single	115 (23.80)			117 (24.20)			94 (19.50)		

*Note. n* for all variables in all groups = 483; Some of the displayed median and range values differed slightly from the original scale due to imputation (i.e., values with two decimals); Decrease = Women whose desire decreased between the two time points (2006 and 2013); Stable = Women whose desire remained stable between the two time points; Increase = Women whose desire increased between the two time points. For sexual desire, sexual arousal, lubrication, orgasm function, sexual satisfaction and sex-related pain, higher scores indicate better function. For sexual distress, depression, anxiety, and body dissatisfaction, higher scores indicate higher distress or dissatisfaction. For too much sexual activity and too little sexual activity, higher scores indicate a greater discrepancy between desired and actual sexual behavior. For sociosexual orientation, higher scores indicate a more liberal sexual orientation.

Edge stability estimations indicated that the magnitude of edges showed variability across bootstrapped estimations, which suggests that the order of edge magnitudes for each network was instable across bootstrapped estimations based on subsamples of the data. However, the edge stability plot also indicated that the estimated edges were very similar across the three groups with large similarities in the order of magnitude. Furthermore, the edge significance plot showed that edges that were significantly different in magnitude from other edges in one group were significantly different from other edges in the other groups as well. This finding further supported our observation that the groups were fairly similar in network structure.

However, we noted three differences in relative edge magnitude between the groups: first, the stable group included a weaker and instable edge between *sexual distress* and *body dissatisfaction* compared to the decrease and increase group; second, the increase group showed a stronger edge between *sexual satisfaction* and *relationship status* compared to the decrease and stable group; third, the increase group showed a weaker and instable edge between *too little sexual activity* and *relationship status* compared to the decrease and stable group.

### Network Connectivity Comparison

Comparing the networks formally only partially supported the results we obtained when comparing the networks visually. The NCT indicated that there were no significant differences in global strength between the three groups (or alternatively, that we did not have enough power to detect a significant difference between the groups^44^; decrease vs. stable *p* = 0.06; stable vs. increase *p* = 0.93; decrease vs. increase *p* = 0.10). Furthermore, the structure invariance test did not indicate that there were significant differences among the nature of edges across the three groups (*p* = 0.53, 0.62, and 0.22 respectively).

### Node Centrality

**Figure 3:**
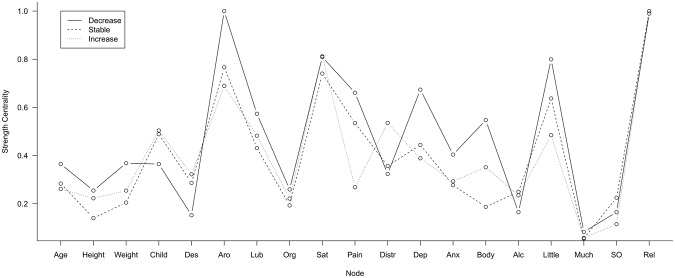
Node strength centrality for all three desire groups at the first time point. Decrease = women whose desire decreased between the two time points (2006 and 2013), Stable = women whose desire remained stable between the two time points, Increase = women whose desire increased between the two time points, Child = number of children, Des = sexual desire, Aro = sexual arousal, Lub = lubrication, Org = orgasm function, Sat = sexual satisfaction, Pain = sex-related pain, Distr = sexual distress, Dep = depression, Anx = anxiety, Body = body dissatisfaction, Alc = (hazardous) alcohol use, Little = too little sexual activity, Much = too much sexual activity, SO = sociosexual orientation, Rel = relationship status.

Node strength centrality is illustrated in Fig. 3 for all three desire groups. Overall, we concluded that, again, there was much similarity in the pattern of centrality of nodes across the three groups. Integrating the observations in Fig. 3 with the strength significance results from the bootstrapped stability analysis (see supplementary material), we concluded that the following nodes were most central across the groups’ networks: *sexual arousal*, *relationship status*, and *sexual satisfaction*. However, we also noted two differences between the groups. *Body dissatisfaction* seemed to be more central in the decrease and increase group than the stable group and *sex-related pain* seemed to be more central in the decrease than the increase group.

### Network Clusters

**Figure 4:**
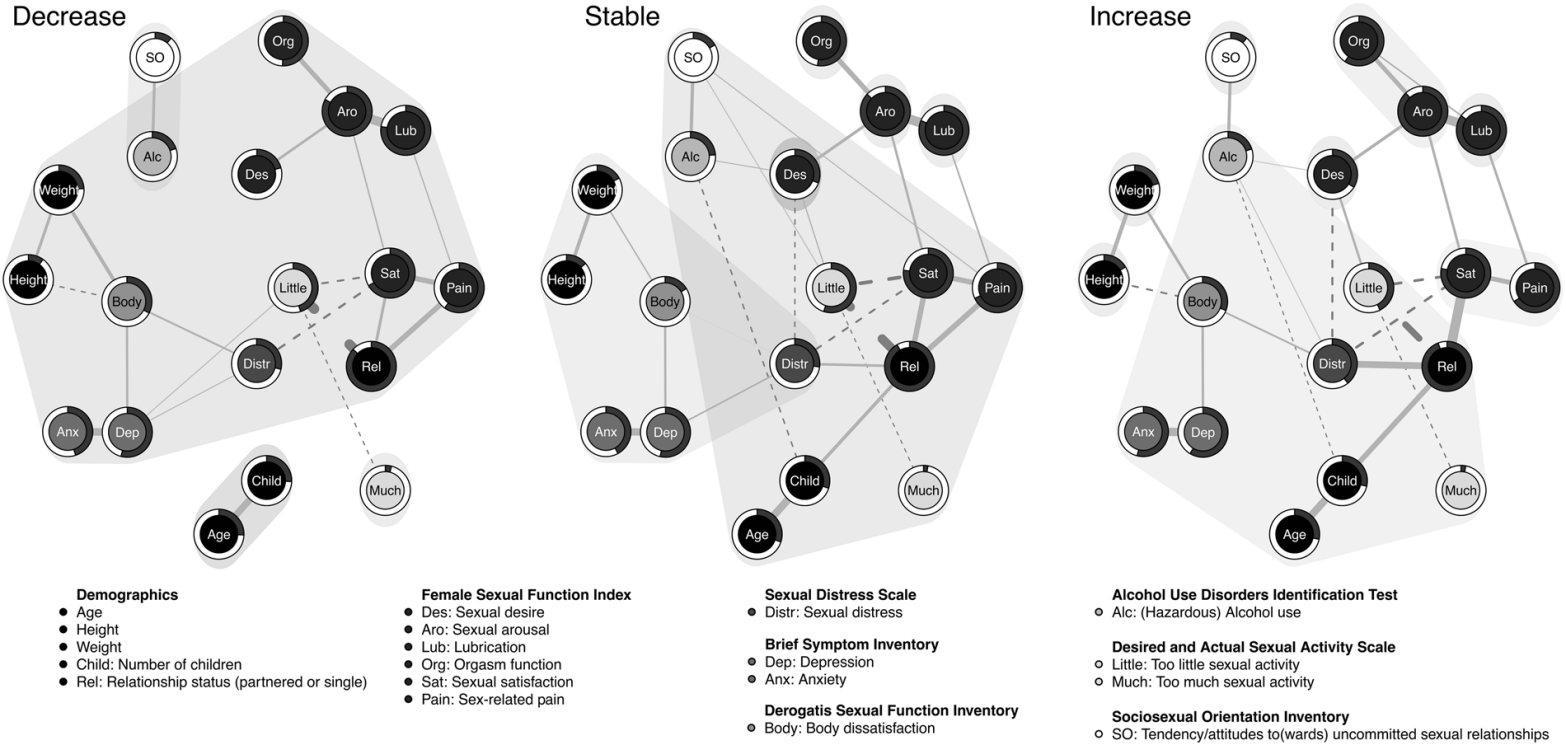
Node clustering based on the walktrap algorithm, visualized on the mixed graphical model (MGM) networks for all three desire groups at the first time point. Note that nodes that are covered by two clusters (i.e., superimposed clouds) do not belong to both clusters; nodes only belong to the cluster by which they are circled in (e.g., Alc is clustered with SO in the decrease group, not with the larger cluster including Weight and Des). Clouds superimposed on single nodes are also considered separate clusters. From left to right: the decrease group shows 4 clusters, the stable group shows 6 clusters, the increase group shows 8 clusters.

The results of the walktrap cluster detection algorithm are presented in Fig. 4. The clustering suggested that the networks became more fragmented going from the decrease to the stable to the increase group (i.e., the number of clusters were 4, 6, and 8, respectively). Even though several observations could be discussed based on these results, we chose to bring the attention to one particular observation. The clusters differentiated across the groups such that in the decrease group, all central nodes (*sexual arousal*, *relationship status*, and *sexual satisfaction*) were members of the same cluster, whereas they belonged to different clusters in the increase group.

### Replication

Finally, we ran all above analyses for the data of the same groups at T2 (see supplementary material for output). Overall, we noticed that the results seemed to partially reverse: (1) The stable group now included the least (rather than most) number of edges (22 vs. 28 and 26 for decrease and increase respectively), and the mean connectivity was 0.09, 0.07, and 0.08 for the decrease, stable and increase group respectively. (2) The stable group only showed a weaker edge between *sexual distress* and *body dissatisfaction* compared to the increase, but not the decrease group. No group showed an edge between *relationship status* and *sexual satisfaction*. The decrease group showed a more instable edge between *too little sexual activity* and *relationship status* than both the stable and the increase group. (3) *Body dissatisfaction* and *sex-related pain* were less (rather than more) central in the decrease group than the other two groups. (4) The number of clusters decreased (rather than increased) from the decrease to the stable and the increase group. Interestingly and importantly, however, the general network structure across groups seemed to replicate well from T1 to T2 as well as the finding that *hormonal contraception* did not relate to any other node at T2 in any of the groups.

## Discussion

In the present study, we investigated predictors of change in experienced levels of sexual desire in women, using a novel network approach to psychopathology. We compared the networks of women whose desire decreased, increased, or remained stable over time. Overall, and contrary to our hypotheses, we found the networks to be fairly similar across the three groups.

Although the networks were fairly similar across groups overall, we did find three potential differences between the desire groups. We choose to interpret these potential group differences cautiously because the formal Network Comparison Test suggested that there were no significant structural differences between the groups. However, the potential differences between the groups’ networks did arise after the regularization procedure had been applied to each network, suggesting that any differences between these networks might not represent false positives, despite the fact that the Network Comparison Test did not find any significant differences in connectivity. It is worth noting that the Network Comparison Test requires much power to detect differences and estimates the relationships between variables slightly differently than in the Mixed Graphical Model, which could explain why our networks showed potential differences that were not picked up in the Network Comparison Test. Replication studies are warranted to study the robustness of the following potential differences.

1. In the visual inspection, the overall network connectivity of the stable group was somewhat less strong compared to the decrease and the increase group, implying that there were more and/or stronger connections between symptoms (either negative or positive) in the groups that had changes in desire. This follows the hypothesis that stronger connections between symptoms in a network could be expected to lead to more changes (due to symptoms influencing each other over time).
2. Body dissatisfaction seemed to play a different role in the groups whose desire changed compared to the stable group: (a) body dissatisfaction was more central in the decrease and increase group than the stable group and (b) the relationship between body dissatisfaction and sexual distress was stronger in the increase and decrease group than in the stable group.
3. Sex-related pain was more central in the decrease group than the increase group. If these two group-differences represent replicable differences, this could imply that body dissatisfaction and sex-related pain might contribute to changes in sexual desire.
4. Lastly, in the cluster analyses, we found that the networks became more fragmented going from the decrease to the stable to the increase group. This implies that changes in nodes with outgoing relationships might spread somewhat more rapidly across the whole network structure in the decrease group compared to the increase group, where such changes would presumably first spread across the subnetworks. This suggests that, for instance, (clinically induced) changes in sexual function could result in changes in general distress (and vice versa) more quickly in the decrease group than in the increase group (i.e., assuming that these relationships are bidirectional).

Overall, these observations suggest that body dissatisfaction and sex-related pain (which are factors that have previously been associated with low sexual desire^8,21^) might be fruitful general points of intervention during clinical work with low sexual desire, in case the observations reflect outgoing relationships from body dissatisfaction and sex-related pain. Note, however, that we estimated between-subject-level networks. Adequate individual interventions should preferably be inferred from within-subject, rather than between-subject-based, networks. See the literature^48^ for a critical discussion on group-to-individual generalizability. However, group-based networks can be useful for generating hypotheses about causality^49^. See the literature^50^ for a critical discussion of network centrality and clinical interventions.

In this section, we discuss four similarities between the networks; that is, we discuss results that pertain to female sexual function in general.

1. Hormonal contraception associated only with relationship status in the increase group, implying that hormonal contraception might play a peripheral role in female sexual functioning. This finding concurs with review studies concerning overall effects of hormonal contraceptives on female sexuality and desire^51,52^.
2. We found relationship status, sexual arousal, and sexual satisfaction to be most central across groups. The results imply that it makes a difference whether a woman has a sexual relationship or not, as women in relationships tended to show higher sexual functioning. These results further suggest that women’s subjective experiences of sexual arousal as well as satisfaction with their sexual life are defining aspects of (self-reported) female sexual function. In case these results represent causal effects descending from sexual arousal and satisfaction, then sexual arousal and sexual satisfaction could be fruitful sexological intervention points for women in general. In case they represent (joint) effects descending from other sexual function aspects, they might act as “gauges” of sexual function showing how women are faring sexually.
3. Sexual distress acted as a bridge between more general distress (anxiety, depression, and body dissatisfaction) and sexual function, implying that the association between general distress and sexual function was mediated by sexual distress or that both converge on or from sexual distress. Furthermore, sexual distress was less closely related to sexual function than sexual satisfaction in all groups. This observation runs counter to a previous study^53^, who argued that distress is more closely related to sexual (dys)function than satisfaction.
4. Finally, we observed that sexual desire and arousal showed different patterns of relationships with other aspects. This finding is noteworthy considering that the Diagnostic and Statistical Manual of Mental Disorders 5 (ref.^9^) combines low sexual desire and low sexual arousal into one diagnosis: Female Sexual Interest/Arousal Disorder. Sexual Arousal and Sexual Desire might represent unique and operationally different sexual function aspects that might require unique classification and treatment.

We would like to highlight some limitations of our study. First, we focused on women whose desire decreased, increased, or remained stable over time. It might be more suitable to study the network structures of women whose desire remained low or high throughout the study. In the present study, however, we were interested in possible factors associated with *changes* in levels of sexual desire, as one could argue that it is clinically relevant to study factors that might contribute to *changes* in rather than differences in levels of sexual desire. It is, however, possible that due to our categorization, the groups at the second time point are not directly comparable to the groups at the first time point. The women in the decrease group, perhaps counter-intuitively, had higher mean levels of sexual desire at the first time point compared to the other two groups (cf. regression toward the mean^54^). In contrast, at the second time point, the women in the decrease group could be more comparable to a low desire group. These dissimilarities between the groups at the first time point and the second time point could (at least partly) explain why some of the results reversed from the first to the second time point and were thus not replicated at the second time point. It is also possible that the groups do not reflect increased, decreased, and stable sexual desire well, as there could have been (nonlinear) fluctuations in sexual desire between the two measurement points.

Second, it is possible that the time frame (seven years) for dividing the participants into groups of women with increased, decreased, and stable desire was not optimal for finding potential differences between the groups. In other words, the change in levels of desire might be associated with group-based network differences at more closely situated points in time rather than differences seven years before the second assessment. At least, the dissimilarities between the two time points did not seem to result from differences in partner change across time between the three groups.

Third, the relationships estimated among the FSFI subscales as well as the relationships of the too much sexual activity variable might have been affected by the ways they are encoded. The FSFI subscales showed strong interrelationships, which might result from participants who did not engage in sexual intercourse scoring 0 automatically on many of the FSFI scales; such “zero imputation” can artificially inflate relationships^55^. Also, the too much sexual activity variable did not relate strongly to any other variable because only few women indicated that they experienced too much sexual activity; a variable that has low variability cannot relate strongly to other variables.

To our knowledge, this is the first study that utilizes network analysis to investigare symptom networks of sexual functioning in women. By looking at female sexual function from a network perspective, we exemplify how differences and similarities in sexual function can be operationalised and assessed in a novel and clinically insightful way (i.e., in terms of differences in network connectivity, centrality, and clustering). We report on differences and similarities in network structure between three groups of women: women whose levels of desire decreased, increased, and remained stable over time. Our results suggest that sex-related pain and body dissatisfaction might play central roles in decreases of sexual desire, suggesting that they might be fruitful points of intervention during clinical work with low sexual desire. However, overall, the network structure of sexual function was surprisingly similar across groups, which ran against our hypotheses. Across groups, we observed that sexual arousal, satisfaction, and relationship status were the most central variables, implying that they might play prominent roles in female sexual function in general. Our study sets the stage for future network modelling of female sexual function and desire. We encourage future longitudinal network studies of sexual function and desire which allow for estimation of individual networks and inference of the direction of relationships.

## Electronic supplementary material

Supplementary Information
