# Supplementary material for: A network analysis of female sexual function: comparing symptom networks in women with decreased, increased, and stable sexual desire
Source: Sci Rep. 2018 Oct 25;8:15815. doi: 10.1038/s41598-018-34138-8 (PMC6202312; doi:10.1038/s41598-018-34138-8)
Supplement: Supplementary file 1 — Supplementary Information [file 41598_2018_34138_MOESM1_ESM.pdf]

SUPPLEMENTARY INFORMATION

A network analysis of female sexual function: comparing symptom networks in women with decreased, increased, and stable sexual desire

Annika Gunst, Marlene Werner, Lourens J. Waldorp, Ellen T. M. Laan, Marianne Källström, Patrick Jern

Data

Data can be made available upon request to the corresponding author, Annika Gunst (annika.gunst@utu.fi).

Measures

Sexual Desire Groups

A short form of the revised Sexual Desire Inventory[1] (SDI-2) was used for creating a grouping variable assessing change in sexual desire over time. The short form included six items (3, 5, 9, 11, 13, and 14) from the original SDI-2, summed together to form a composite variable. In the present study, the short-form SDI-2 demonstrated acceptable internal consistency at T1 ($\alpha = .79$) and good internal consistency at T2 ($\alpha = .84$).

Measures Included in the Networks

Female sexual function. The Female Sexual Function Index[2] (FSFI) was used to assess sexual function. The FSFI includes 19 Likert-type items, assessing six subdomains of sexual

function over the past four weeks: *sexual desire*, (subjective) *sexual arousal*, *lubrication*, *orgasm function*, *sexual satisfaction*, and *sex-related pain*. Item scores range from 1 to 5 or 0 to 5 (0 representing the supplementary option "no sexual activity / did not attempt intercourse") with lower scores indicating worse sexual function. We added the response option “no partnered activity” to items 14 and 15, which analogous to the response options of other items, was given the value of 0. We calculated domain-scores according to the guidelines[2].

The FSFI has repeatedly demonstrated good validity and reliability in different settings[2,3], including the Genetics of Sex and Aggression data collection from 2006[4]. In the present study, the FSFI demonstrated acceptable to excellent internal consistency for all six domains at both time points (Cronbach's α’s ranging from .77 to .98). Due to a technical error in the data collection phase, one item was omitted from the T2 data collection (item 18: ‘*Over the past 4 weeks, how often did you experience discomfort or pain following vaginal penetration?*’). Thus, the composite variable for *sex-related pain* included two items instead of three items the present study, both at T1 and T2. Nevertheless, the composite variable demonstrated excellent internal consistency at both time points ($\alpha_{T1} = .97$, $\alpha_{T2} = .98$).

Sexual distress. A short form of the Female Sexual Distress Scale[5] (FSDS) was used to assess sexual distress. The short form included seven Likert-type items from the original scale (1, 3, 5, 8, 9, 10, and 11), with a sum score ranging from 0 to 28. Higher scores indicate higher sexual distress. The FSDS has previously demonstrated good discriminant validity and test-retest reliability[6]. In the present study, the questionnaire demonstrated good internal consistency at T1 ($\alpha = .89$) and excellent internal consistency at T2 ($\alpha = .92$).

Psychological distress. Two subscales from the Brief Symptom Inventory-18[7] (BSI-18) were used to assess symptoms of anxiety and depression. Both subscales include six Likert-type

items, summed together to form composite variables ranging from 6 to 30 with higher scores indicating higher psychological distress. The BSI-18 has previously demonstrated good psychometric properties in various clinical population-based samples[8]. In the present study, both the *anxiety* subscale (α_{T1} and α_{T2} = .85) and the *depression* subscale (α_{T1} = .84, α_{T2} = .85) demonstrated good internal consistency at both time points.

Body dissatisfaction. The body image subscale of the Derogatis Sexual Function Inventory[9] (DSFI) was used to assess body dissatisfaction. Eleven gender-neutral Likert-type items were summed together to form a composite variable ranging from 11 to 55 with higher scores indicating higher body dissatisfaction. The DSFI has previously demonstrated sound psychometric properties in various settings measuring sexual function[10]. In the present study, the *body dissatisfaction* composite variable demonstrated acceptable internal consistency at both time points (α_{T1} = .70, α_{T2} = .75).

Alcohol use. The Alcohol Use Disorders Identification Test[11] (AUDIT) was used to assess (hazardous) alcohol use. The AUDIT consists of ten Likert-type items, summed together to form a composite variable ranging from 0 to 40 with higher scores indicating more frequent and hazardous alcohol use. The AUDIT is one of the most frequently used screening measures of alcohol use and related problems, and has previously demonstrated sound psychometric properties[12]. In the present study, the AUDIT demonstrated good internal consistency at both time points (α_{T1} and α_{T2} = .81).

Discrepancy in desired and actual sexual activity. The Desired and Actual Sexual Activity Scale[13], a modified version of the drive subscale of the DSFI[9], was used to assess discrepancy in desired and actual frequency of sexual behaviors. The DASA consists of 12 nine-point Likert-type items; 6 items inquiring about how frequently the respondent engages in

specific sexual behaviors (e.g., masturbation and oral sex) and 6 items inquiring about how frequently the respondent desires the specific sexual behaviors. Two discrepancy variables were created in the present study. First, the actual sexual activity items were subtracted from the desired sexual activity items, resulting in a discrepancy variable with a score range between -54 (indicating that the respondent had engaged in more sexual activity than she desired) and 54 (indicating that the respondent had engaged in less sexual activity than she desired). This variable was further divided into two variables: one ranging from -54 to 0 (i.e., positive values were replaced with 0) representing a variable of *too much sexual activity*, and another ranging from 0 to 54 (i.e., negative values were replaced with 0) representing a variable of *too little sexual activity*. For clarity, the absolute values were used for the *too much sexual activity* variable, meaning that higher scores indicated a bigger discrepancy between desired and actual behavior for both the *too much sexual activity* and the *too little sexual activity* variable.

Sociosexual orientation. The Sociosexual Orientation Inventory[14] (SOI) was used to assess sociosexual orientation. Sociosexual orientation refers to an individual's attitudes towards and tendency to engage in casual and uncommitted sexual relations. The SOI includes four nine-point Likert-type items measuring attitudes and three open ended numerical items measuring behaviors. The items were summed together according to the original formula[14] to form a composite variable with higher scores indicating a more liberal sexual orientation.

References

1. Spector, I. P., Carey, M. P. & Steinberg, L. The sexual desire inventory: development, factor structure, and evidence of reliability. *J. Sex Marital Ther.* **22,** 175-190 (1996).
2. Rosen, R. *et al.* The Female Sexual Function Index (FSFI): a multidimensional self-report

instrument for the assessment of female sexual function. *J. Sex Marital Ther.* **26,** 191-208 (2000).

3. Wiegel, M., Meston, C. & Rosen, R. The Female Sexual Function Index (FSFI): cross-validation and development of clinical cutoff scores. *J. Sex Marital Ther.* **31,** 1-20 (2005).
4. Witting, K. *et al.* Evaluation of the Female Sexual Function Index in a population based sample from Finland. *Arch. Sex. Behav.* **37,** 912-924 (2008).
5. Derogatis, L. R. *et al.* The Female Sexual Distress Scale (FSDS): initial validation of a standardized scale for assessment of sexually related personal distress in women. *J. Sex Marital Ther.* **28,** 317-330 (2002).
6. Derogatis, L., Clayton, A., Lewis-D'Agostino, D., Wunderlich, G. & Fu, Y. Validation of the female sexual distress scale-revised for assessing distress in women with hypoactive sexual desire disorder. *J. Sex. Med.* **5,** 357-364 (2008).
7. Derogatis, L. R. *Brief Symptom Inventory (BSI)-18. Administration, Scoring and Procedures Manual* (Pearson, 2001).
8. Derogatis, L. R. & Savitz, K. L. The SCL-90-R and Brief Symptom Inventory (BSI) in primary care in *Handbook of Psychological Assessment in Primary Care Settings (*ed. M. E. Maruish) 297-334 (Lawrence Erlbaum Associates, 2000).
9. Derogatis, L. R. & Melisarotos, N. The DSFI: a multidimensional measure of sexual functioning. *J. Sex Marital Ther.* **5,** 244-281 (1979).
10. Daker-White, G. Reliable and valid self-report outcome measures in sexual (dys)function: a systematic review. *Arch. Sex. Behav.* **31,** 197-209 (2002).
11. Babor, T. F., Higgins-Biddle, J. C., Saunders, J. B. & Monteiro, M. G. *The Alcohol Use Disorders Identification Test Guidelines for Use in Primary Care. Second Edition* (World Health Organization, Department of Mental Health and Substance Dependence, 2001).

12. Allen, J. P., Litten, R. Z., Fertig, J. B., Babor, T. A review of research on the Alcohol Use Disorders Identification Test (AUDIT). *Alcohol. Clin. Exp. Res.* **21,** 613-619 (1997).

13. Santtila, P. *et al.* Discrepancies between sexual desire and sexual activity: gender differences and associations with relationship satisfaction. *J. Sex Marital Ther.* **34,** 31-44 (2007).

14. Simpson, J. A. & Gangestad, S. W. Individual differences in sociosexuality: evidence for convergent and discriminant validity. *J. Pers. Soc. Psychol* **60,** 870-883 (1991).

Statistical Analyses

Table of Contents

1. Data Import
2. Data Preparation
3. Data Descriptives
4. Network Estimation (incl. hormonal contraception)
5. Network Visualization (incl. hormonal contraception)
6. Network Estimation (excl. hormonal contraception)
7. Network Visualization (excl. hormonal contraception)
8. Network Stability
9. Network Comparison
10. Network Communities
11. Replication - Data Import and Preparation
12. Replication - Network Estimation (incl. hormonal contraception)
13. Replication - Network Estimation (excl. hormonal contraception)
14. Replication - Network Visualization (excl. hormonal contraception)
15. Replication - Network Stability
16. Replication - Network Comparison
17. Replication - Network Communities

1. Data Import

```
##################################
# 1. Data Import First Timepoint#
##################################
setwd("C:/Users/Lene/Desktop/FSFI Collaboration/Datasets/Supplementary Material")
library(haven)
FSFI_Gunstetal_Imputed_NegTo0 <- read_sav("C:/Users/Lene/Desktop/FSFI Collaboration/Datasets/FS
FI_Gunstetal_Imputed_NegTo0.sav")
```

2. Data Preparation

```
########################
# 2. Data preparation #
########################

DataNetwork <- FSFI_Gunstetal_Imputed_NegTo0[,3:22]
DataNetwork[,21] <- FSFI_Gunstetal_Imputed_NegTo0$SDI_groups
DataNetwork[,22] <- FSFI_Gunstetal_Imputed_NegTo0$SDI_change

colnames(DataNetwork)[21] <- "SDI_groups"
colnames(DataNetwork)[22] <- "SDI_change"
```

3. Data Descriptive Statistics

```
# check whether there are any variables with missing values left
apply(apply(DataNetwork, 2, is.na), 2, sum) # no NA
##            age_OLD        height_OLD        weight_OLD            hc_OLD
##                  0                 0                 0                 0
##       biolchild_OLD       FSFIdes_OLD       FSFIaro_OLD       FSFIlub_OLD
##                  0                 0                 0                 0
##        FSFIorg_OLD       FSFIsat_OLD      FSFIpain_OLD           SDS_OLD
##                  0                 0                 0                 0
##         BSIdep_OLD        BSIanx_OLD            BI_OLD         AUDIT_OLD
##                  0                 0                 0                 0
## DSFItoolittle_OLD   DSFItoomuch_OLD           SOI_OLD     haspartner_OLD
##                  0                 0                 0                 0
##         SDI_groups        SDI_change
##                  0                 0

cor(DataNetwork)
##                        age_OLD   height_OLD   weight_OLD       hc_OLD
## age_OLD            1.000000000  0.026286749  0.169905620 -0.129566839
## height_OLD         0.026286749  1.000000000  0.344403818  0.013777569
## weight_OLD         0.169905620  0.344403818  1.000000000 -0.041399265
## hc_OLD            -0.129566839  0.013777569 -0.041399265  1.000000000
## biolchild_OLD      0.553024853 -0.015544891  0.122468948 -0.114682329
## FSFIdes_OLD       -0.158600184 -0.011732344 -0.004808011  0.051986124
## FSFIaro_OLD        0.066602656  0.011364167  0.007128246  0.105847045
## FSFIlub_OLD        0.069994684  0.017092991  0.017119480  0.116073879
## FSFIorg_OLD        0.139823381  0.015797078  0.047068992  0.028293057
## FSFIsat_OLD        0.046719679 -0.016479377 -0.038709108  0.211418912
## FSFIpain_OLD       0.135371696  0.055805252  0.037091493  0.205547420
## SDS_OLD            0.036611918 -0.037451549  0.044780692 -0.048357103
## BSIdep_OLD        -0.109035675 -0.030893066  0.029177358 -0.036115628
## BSIanx_OLD        -0.125321488 -0.031507516 -0.034318398 -0.008353472
## BI_OLD            -0.036467471 -0.116201100  0.282914633 -0.082925802
## AUDIT_OLD         -0.171922696 -0.013499062  0.032441472  0.065568794
## DSFItoolittle_OLD -0.065208434 -0.002056568  0.062283886 -0.117266029
## DSFItoomuch_OLD    0.037159678 -0.034300268 -0.036211889 -0.021652321
## SOI_OLD            0.075946510  0.017580255  0.084583548  0.024438576
## haspartner_OLD     0.191395727 -0.014633106  0.001151473  0.188922760
## SDI_groups        -0.007982908  0.005344832  0.009586199  0.019841234
## SDI_change        -0.006013512  0.016108854  0.007035689  0.022717709
##                   biolchild_OLD  FSFIdes_OLD  FSFIaro_OLD  FSFIlub_OLD
## age_OLD             0.553024853 -0.158600184  0.066602656  0.069994684
## height_OLD         -0.015544891 -0.011732344  0.011364167  0.017092991
## weight_OLD          0.122468948 -0.004808011  0.007128246  0.017119480
## hc_OLD             -0.114682329  0.051986124  0.105847045  0.116073879
## biolchild_OLD       1.000000000 -0.088111575  0.085649735  0.086861428
## FSFIdes_OLD        -0.088111575  1.000000000  0.441855986  0.334567339
```

```
## FSFIaro_OLD         0.085649735  0.441855986  1.000000000  0.899402740
## FSFIlub_OLD         0.086861428  0.334567339  0.899402740  1.000000000
## FSFIorg_OLD         0.153792430  0.243985692  0.746921907  0.703853945
## FSFIsat_OLD         0.119966743  0.299865876  0.678226212  0.647558766
## FSFIpain_OLD        0.188080729  0.215881099  0.603903882  0.643406041
## SDS_OLD            -0.015576057 -0.242072473 -0.241864866 -0.145039989
## BSIdep_OLD         -0.083599516  0.021111699 -0.159799617 -0.137973383
## BSIanx_OLD         -0.060763923  0.072267129 -0.098326574 -0.075064116
## BI_OLD             -0.002154330 -0.108127152 -0.183241501 -0.134036418
## AUDIT_OLD          -0.205861974  0.207832652  0.104553534  0.108883719
## DSFItoolittle_OLD  -0.140972947  0.075255684 -0.245232790 -0.279415900
## DSFItoomuch_OLD     0.049212237 -0.060309219 -0.033565309 -0.008889944
## SOI_OLD             0.007744323  0.154122379  0.109679984  0.097243532
## haspartner_OLD      0.216884564  0.081102801  0.399819735  0.427587487
## SDI_groups          0.031060566 -0.154437586 -0.090639955 -0.075902013
## SDI_change          0.035612623 -0.201990379 -0.105756365 -0.082546771
##                    FSFIorg_OLD  FSFIsat_OLD FSFIpain_OLD     SDS_OLD
## age_OLD            0.139823381  0.046719679  0.135371696  0.03661192
## height_OLD         0.015797078 -0.016479377  0.055805252 -0.03745155
## weight_OLD         0.047068992 -0.038709108  0.037091493  0.04478069
## hc_OLD             0.028293057  0.211418912  0.205547420 -0.04835710
## biolchild_OLD      0.153792430  0.119966743  0.188080729 -0.01557606
## FSFIdes_OLD        0.243985692  0.299865876  0.215881099 -0.24207247
## FSFIaro_OLD        0.746921907  0.678226212  0.603903882 -0.24186487
## FSFIlub_OLD        0.703853945  0.647558766  0.643406041 -0.14503999
## FSFIorg_OLD        1.000000000  0.505977015  0.433936468 -0.22411195
## FSFIsat_OLD        0.505977015  1.000000000  0.755847134 -0.35936046
## FSFIpain_OLD       0.433936468  0.755847134  1.000000000 -0.17629530
## SDS_OLD           -0.224111949 -0.359360457 -0.176295298  1.00000000
## BSIdep_OLD        -0.157038229 -0.251549353 -0.159838540  0.37395858
## BSIanx_OLD        -0.123474750 -0.116658825 -0.076692419  0.32713673
## BI_OLD            -0.156999934 -0.208781570 -0.146122660  0.34253970
## AUDIT_OLD         -0.001343699  0.006748583  0.059143282  0.12698738
## DSFItoolittle_OLD -0.221013112 -0.538806064 -0.434677671  0.19581689
## DSFItoomuch_OLD   -0.009370435  0.039744947  0.047522034  0.02803851
## SOI_OLD            0.015379267  0.028864166  0.110870841  0.02748733
## haspartner_OLD     0.314389620  0.643055966  0.632449098 -0.04810504
## SDI_groups        -0.055456791 -0.006424257 -0.012644879  0.01425262
## SDI_change        -0.057887062 -0.005970852 -0.003337505  0.01698209
##                    BSIdep_OLD   BSIanx_OLD      BI_OLD    AUDIT_OLD
## age_OLD           -0.10903567 -0.125321488 -0.03646747 -0.171922696
## height_OLD        -0.03089307 -0.031507516 -0.11620110 -0.013499062
## weight_OLD         0.02917736 -0.034318398  0.28291463  0.032441472
## hc_OLD            -0.03611563 -0.008353472 -0.08292580  0.065568794
## biolchild_OLD     -0.08359952 -0.060763923 -0.00215433 -0.205861974
## FSFIdes_OLD        0.02111170  0.072267129 -0.10812715  0.207832652
## FSFIaro_OLD       -0.15979962 -0.098326574 -0.18324150  0.104553534
## FSFIlub_OLD       -0.13797338 -0.075064116 -0.13403642  0.108883719
## FSFIorg_OLD       -0.15703823 -0.123474750 -0.15699993 -0.001343699
## FSFIsat_OLD       -0.25154935 -0.116658825 -0.20878157  0.006748583
## FSFIpain_OLD      -0.15983854 -0.076692419 -0.14612266  0.059143282
## SDS_OLD            0.37395858  0.327136728  0.34253970  0.126987381
## BSIdep_OLD         1.00000000  0.699078928  0.38785277  0.225342801
## BSIanx_OLD         0.69907893  1.000000000  0.25878846  0.249435233
## BI_OLD             0.38785277  0.258788462  1.00000000  0.103043308
## AUDIT_OLD          0.22534280  0.249435233  0.10304331  1.000000000
## DSFItoolittle_OLD  0.25233862  0.112831588  0.14471601  0.127408954
## DSFItoomuch_OLD    0.02370179  0.043392691  0.04673992 -0.006716973
## SOI_OLD            0.11283699  0.079464057  0.01633989  0.341677274
## haspartner_OLD    -0.16614574 -0.042841563 -0.12790931 -0.083739426
## SDI_groups         0.02578302  0.033911659  0.03508038 -0.085544567
## SDI_change         0.01362354  0.024294324  0.03100555 -0.104291038
##                   DSFItoolittle_OLD DSFItoomuch_OLD      SOI_OLD
```

```
## age_OLD               -0.065208434    0.037159678  0.075946510
## height_OLD            -0.002056568   -0.034300268  0.017580255
## weight_OLD             0.062283886   -0.036211889  0.084583548
## hc_OLD                -0.117266029   -0.021652321  0.024438576
## biolchild_OLD         -0.140972947    0.049212237  0.007744323
## FSFIdes_OLD            0.075255684   -0.060309219  0.154122379
## FSFIaro_OLD           -0.245232790   -0.033565309  0.109679984
## FSFIlub_OLD           -0.279415900   -0.008889944  0.097243532
## FSFIorg_OLD           -0.221013112   -0.009370435  0.015379267
## FSFIsat_OLD           -0.538806064    0.039744947  0.028864166
## FSFIpain_OLD          -0.434677671    0.047522034  0.110870841
## SDS_OLD                0.195816890    0.028038505  0.027487326
## BSIdep_OLD             0.252338622    0.023701790  0.112836993
## BSIanx_OLD             0.112831588    0.043392691  0.079464057
## BI_OLD                 0.144716008    0.046739918  0.016339895
## AUDIT_OLD              0.127408954   -0.006716973  0.341677274
## DSFItoolittle_OLD      1.000000000   -0.204318304  0.127275481
## DSFItoomuch_OLD       -0.204318304    1.000000000  0.029169988
## SOI_OLD                0.127275481    0.029169988  1.000000000
## haspartner_OLD        -0.589114197    0.064522766 -0.019818780
## SDI_groups            -0.068915731    0.034473712 -0.073218732
## SDI_change            -0.107234469    0.059502478 -0.091306208
##                    haspartner_OLD   SDI_groups   SDI_change
## age_OLD               0.191395727 -0.007982908 -0.006013512
## height_OLD           -0.014633106  0.005344832  0.016108854
## weight_OLD            0.001151473  0.009586199  0.007035689
## hc_OLD                0.188922760  0.019841234  0.022717709
## biolchild_OLD         0.216884564  0.031060566  0.035612623
## FSFIdes_OLD           0.081102801 -0.154437586 -0.201990379
## FSFIaro_OLD           0.399819735 -0.090639955 -0.105756365
## FSFIlub_OLD           0.427587487 -0.075902013 -0.082546771
## FSFIorg_OLD           0.314389620 -0.055456791 -0.057887062
## FSFIsat_OLD           0.643055966 -0.006424257 -0.005970852
## FSFIpain_OLD          0.632449098 -0.012644879 -0.003337505
## SDS_OLD              -0.048105036  0.014252621  0.016982085
## BSIdep_OLD           -0.166145743  0.025783020  0.013623544
## BSIanx_OLD           -0.042841563  0.033911659  0.024294324
## BI_OLD               -0.127909305  0.035080384  0.031005546
## AUDIT_OLD            -0.083739426 -0.085544567 -0.104291038
## DSFItoolittle_OLD    -0.589114197 -0.068915731 -0.107234469
## DSFItoomuch_OLD       0.064522766  0.034473712  0.059502478
## SOI_OLD              -0.019818780 -0.073218732 -0.091306208
## haspartner_OLD        1.000000000  0.035148707  0.044823579
## SDI_groups            0.035148707  1.000000000  0.885106810
## SDI_change            0.044823579  0.885106810  1.000000000
# SDI group variable is not too strongly correlated with other variables
# this would be a problem because we might otherwise condition on a latent effect!

#####################################################
# Histograms of all variables to check distributions#
#####################################################
colnames(DataNetwork) # to use with histograms
##  [1] "age_OLD"           "height_OLD"        "weight_OLD"
##  [4] "hc_OLD"            "biolchild_OLD"     "FSFIdes_OLD"
##  [7] "FSFIaro_OLD"       "FSFIlub_OLD"       "FSFIorg_OLD"
## [10] "FSFIsat_OLD"       "FSFIpain_OLD"      "SDS_OLD"
## [13] "BSIdep_OLD"        "BSIanx_OLD"        "BI_OLD"
## [16] "AUDIT_OLD"         "DSFItoolittle_OLD" "DSFItoomuch_OLD"
## [19] "SOI_OLD"           "haspartner_OLD"    "SDI_groups"
## [22] "SDI_change"

apply(DataNetwork, 2, hist)
```

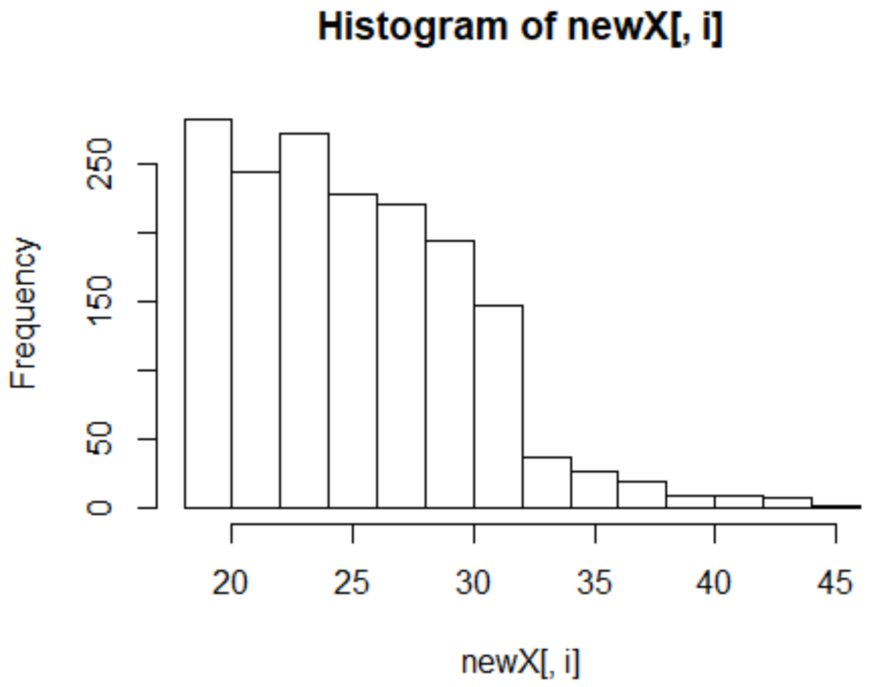

Histogram of newX[, i]
Frequency
0
50
150
250
20
25
30
35
40
45
newX[, i]

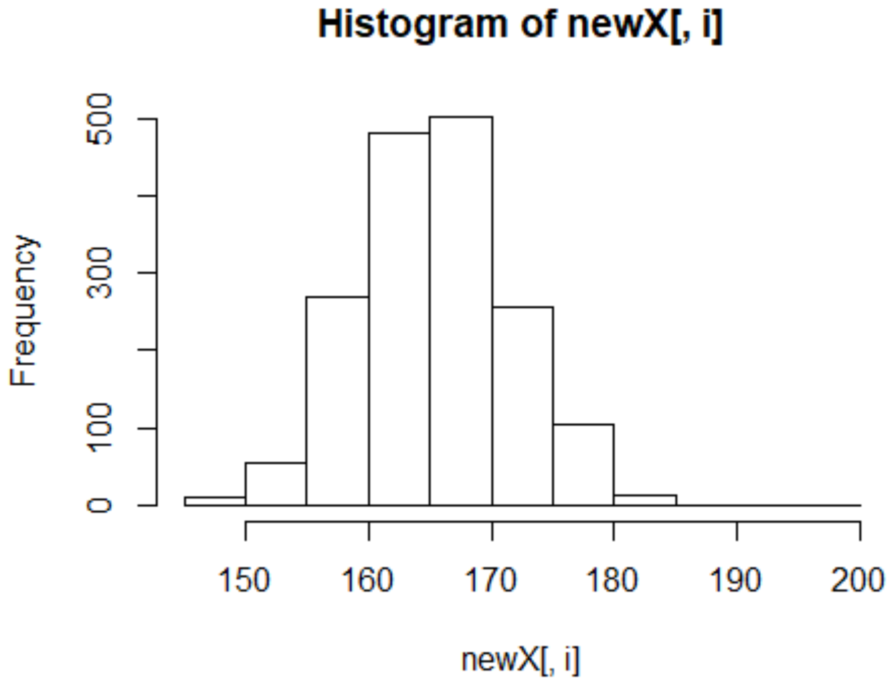

Histogram of newX[, i]
Frequency
0
100
300
500
150
160
170
180
190
200
newX[, i]

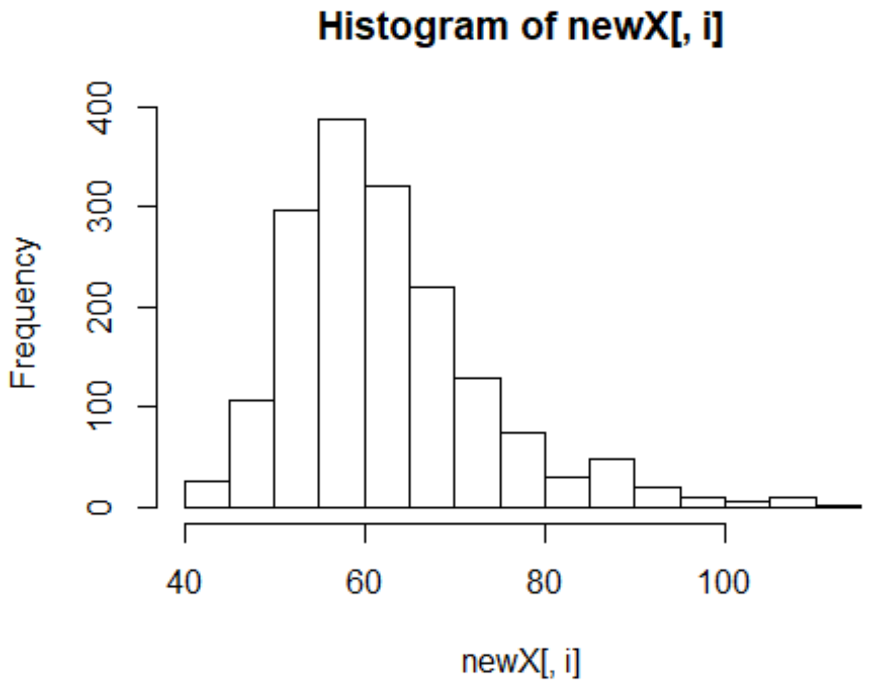

Histogram of newX[, i]
Frequency
0
100
200
300
400
40
60
80
100
newX[, i]

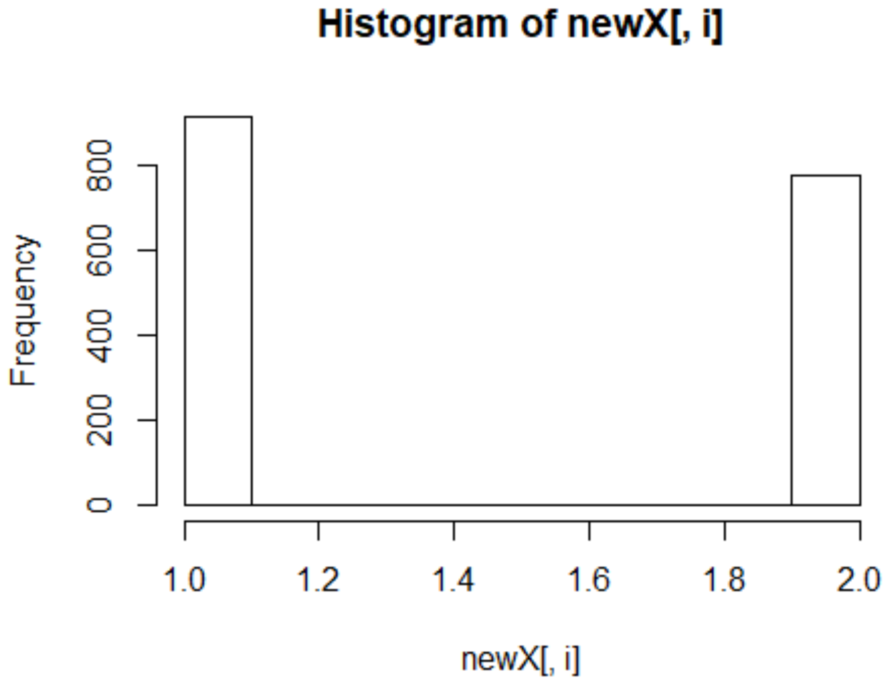

Histogram of newX[, i]
Frequency
0
200
400
600
800
1.0
1.2
1.4
1.6
1.8
2.0
newX[, i]

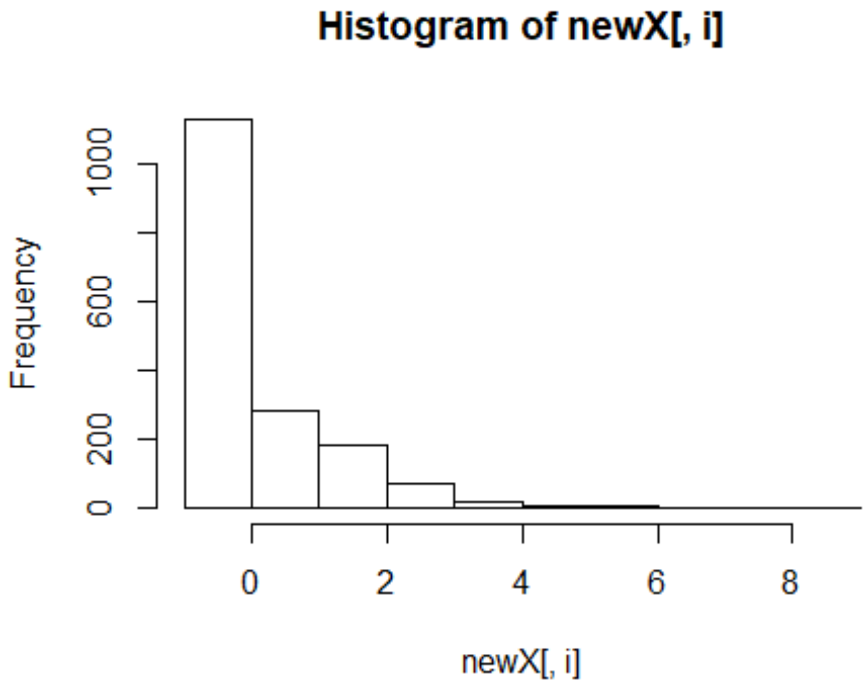
Histogram of newX[, i]
Frequency
0
200
600
1000
0
2
4
6
8
newX[, i]

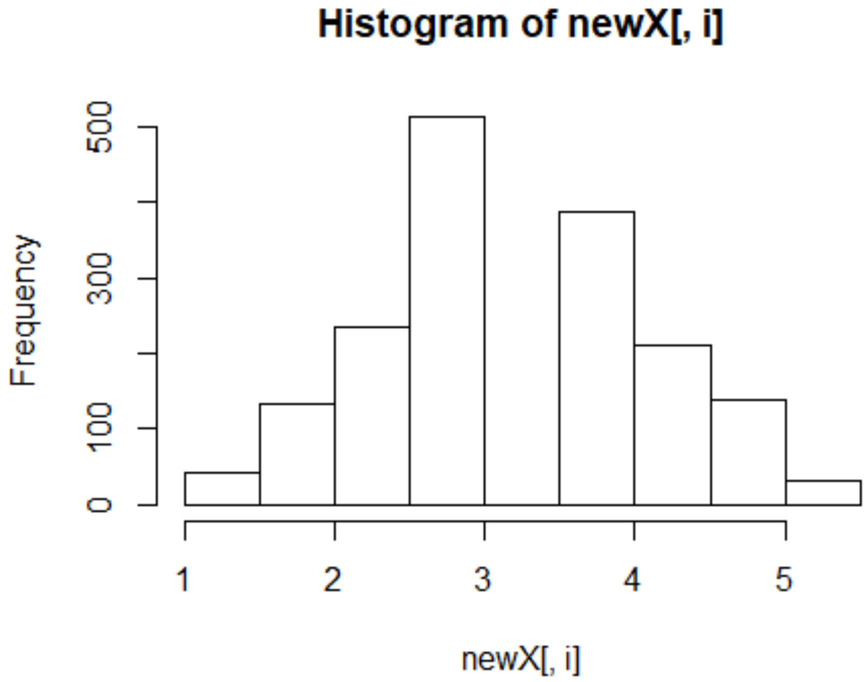
Histogram of newX[, i]
Frequency
0
100
300
500
1
2
3
4
5
newX[, i]

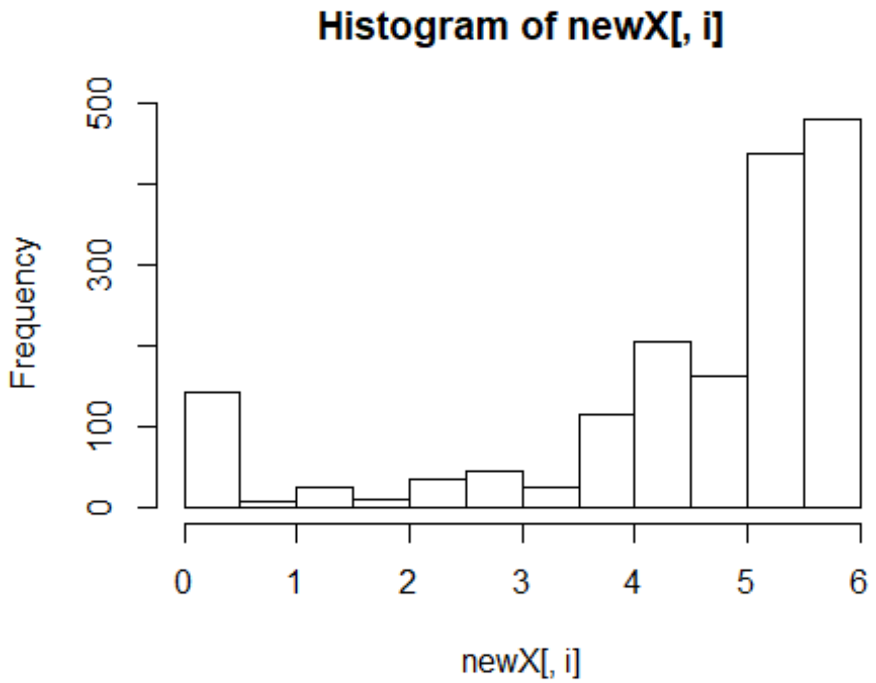
Histogram of newX[, i]
Frequency
0
100
300
500
0
1
2
3
4
5
6
newX[, i]

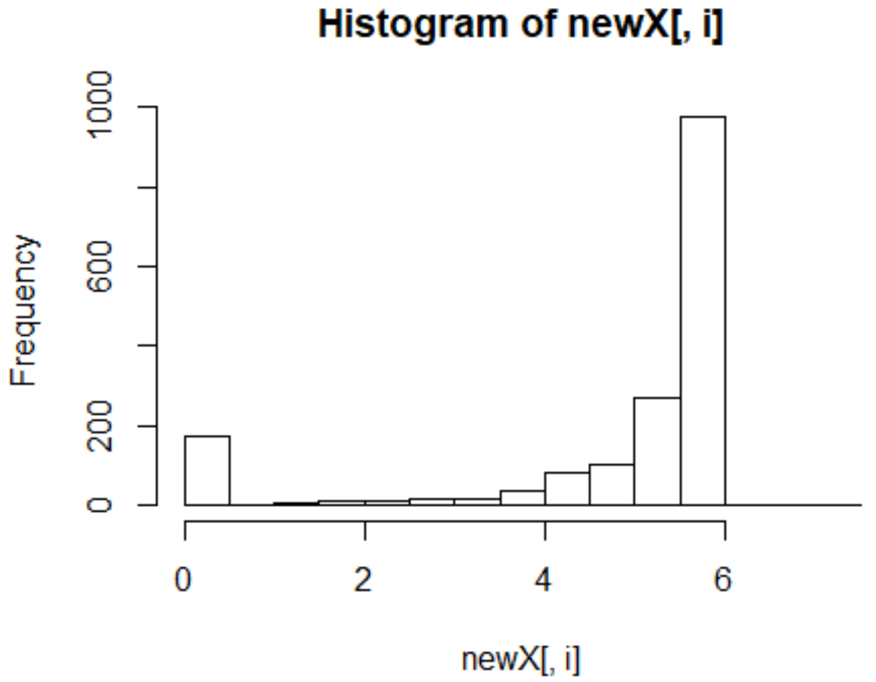
Histogram of newX[, i]
Frequency
0
200
600
1000
0
2
4
6
newX[, i]

Histogram of newX[, i]

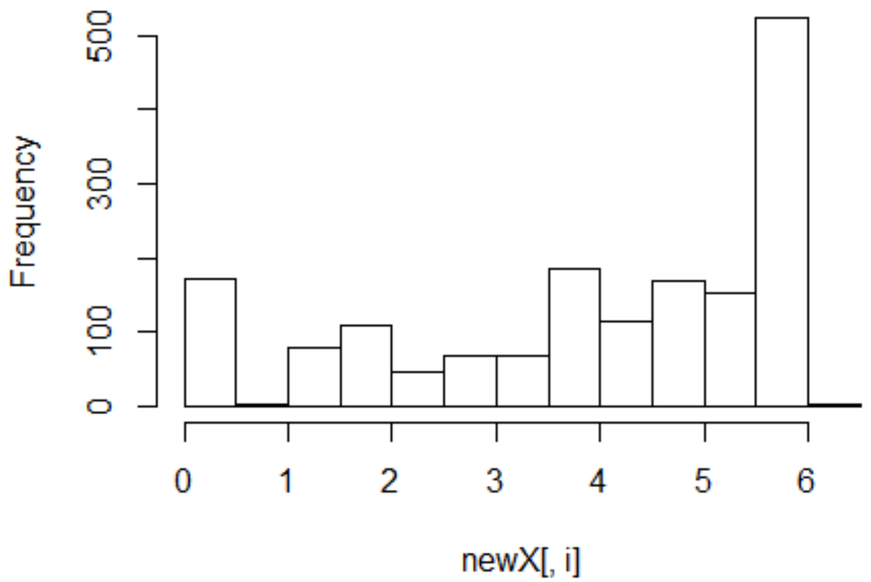

Histogram of newX[, i]

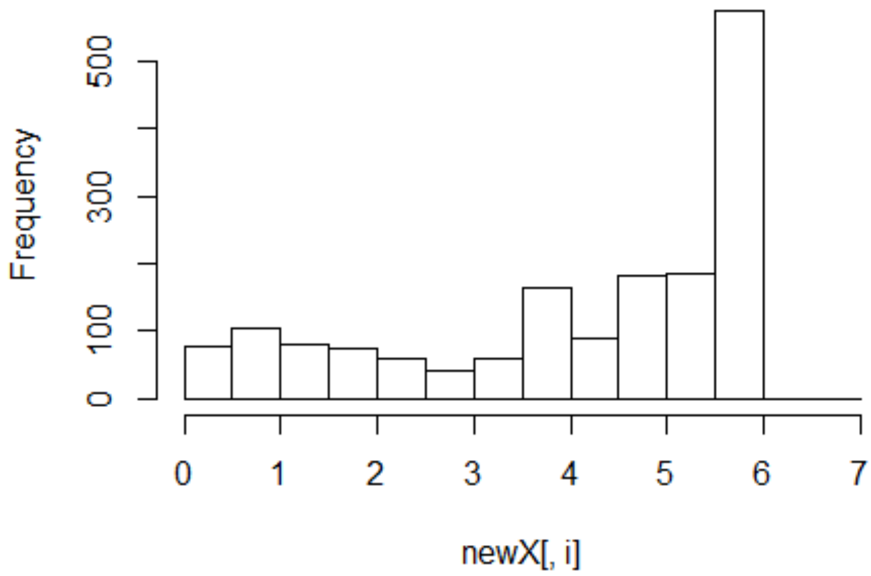

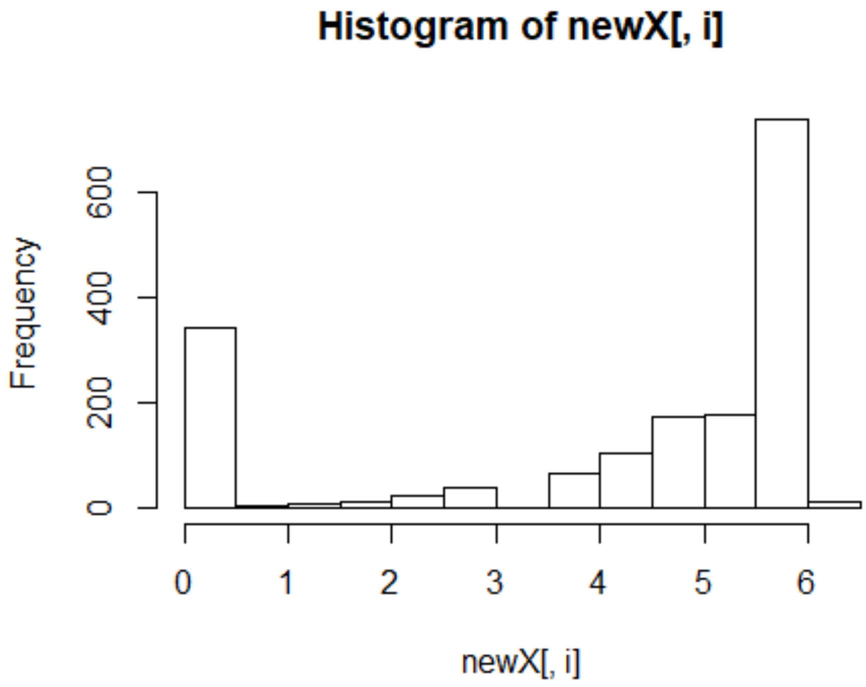
Histogram of newX[, i]
Frequency
0
200
400
600
0
1
2
3
4
5
6
newX[, i]

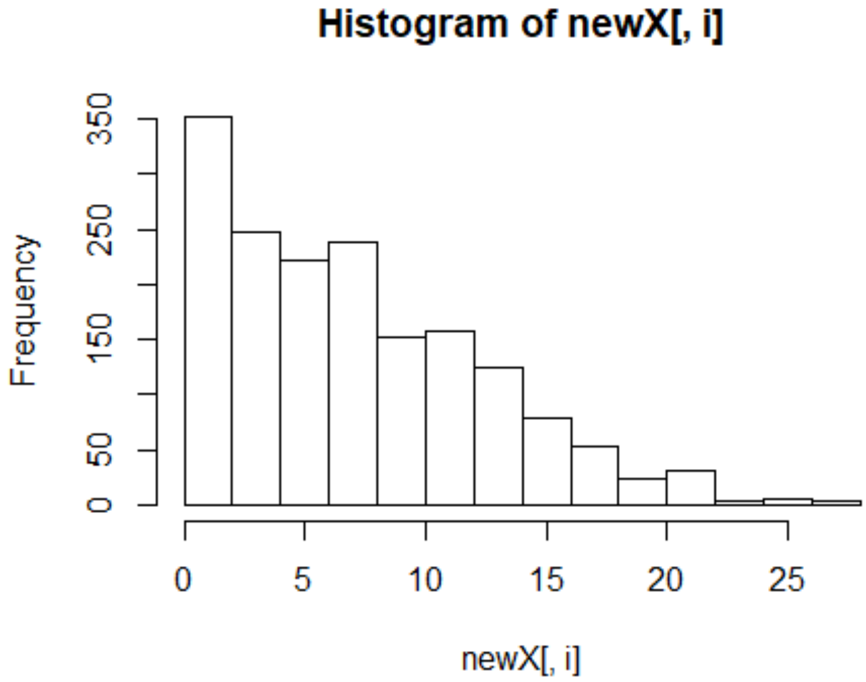
Histogram of newX[, i]
Frequency
0
50
150
250
350
0
5
10
15
20
25
newX[, i]

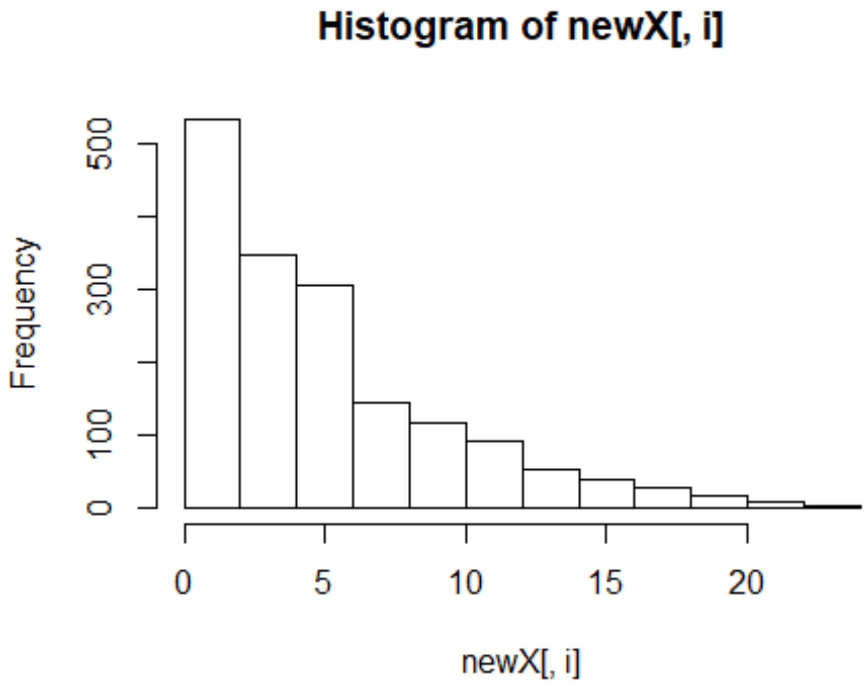
Histogram of newX[, i]
Frequency
0
100
300
500
0
5
10
15
20
newX[, i]

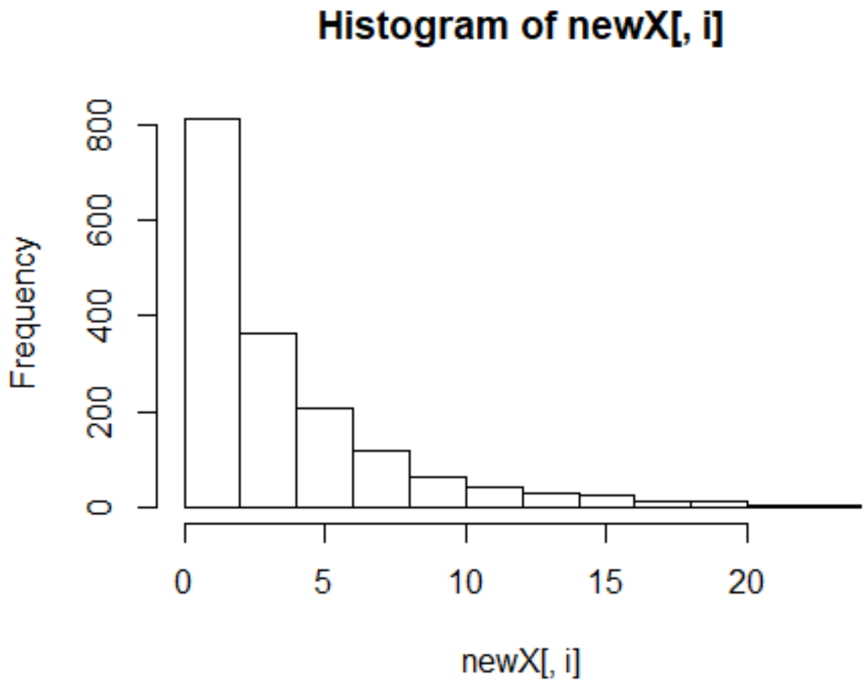
Histogram of newX[, i]
Frequency
0
200
400
600
800
0
5
10
15
20
newX[, i]

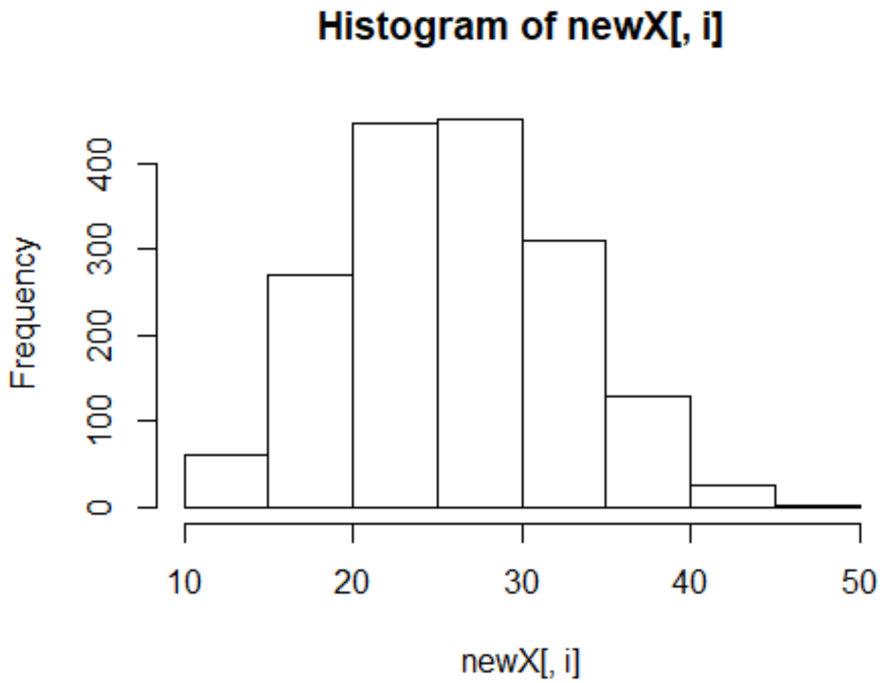
Histogram of newX[, i]
Frequency
0
100
200
300
400
10
20
30
40
50
newX[, i]

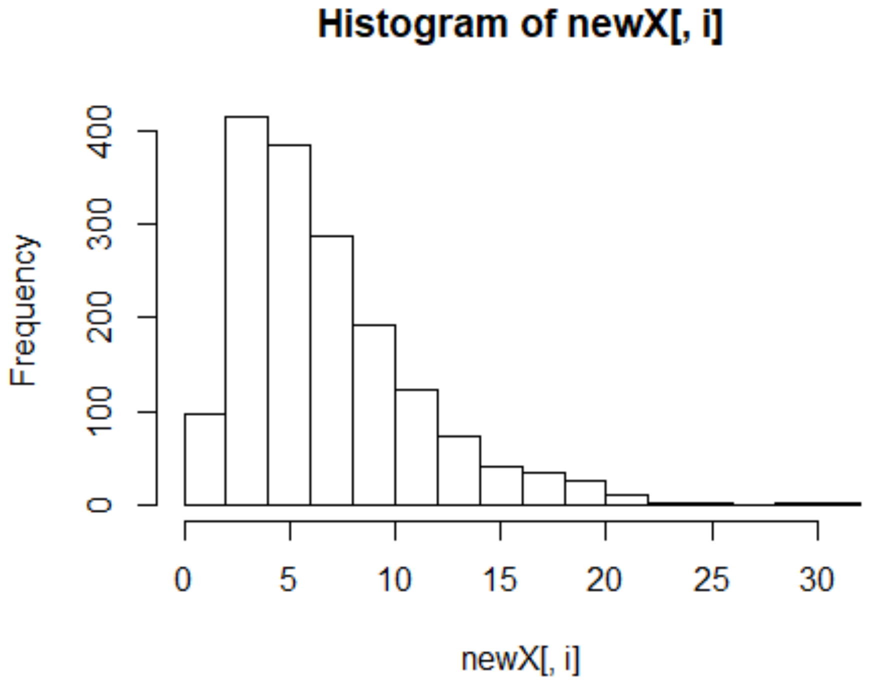
Histogram of newX[, i]
Frequency
0
100
200
300
400
0
5
10
15
20
25
30
newX[, i]

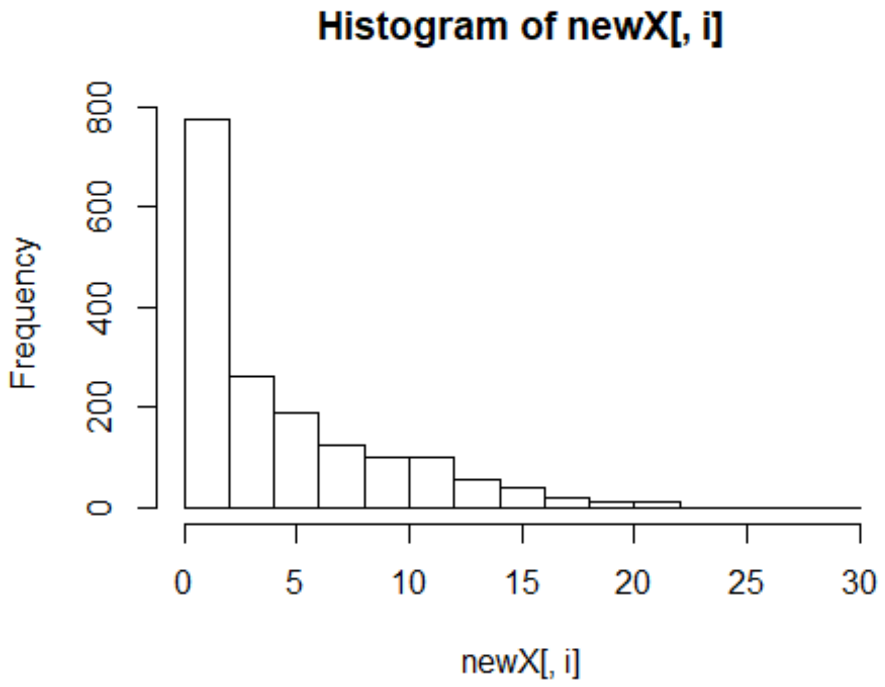
Histogram of newX[, i]
Frequency
0
200
400
600
800
0
5
10
15
20
25
30
newX[, i]

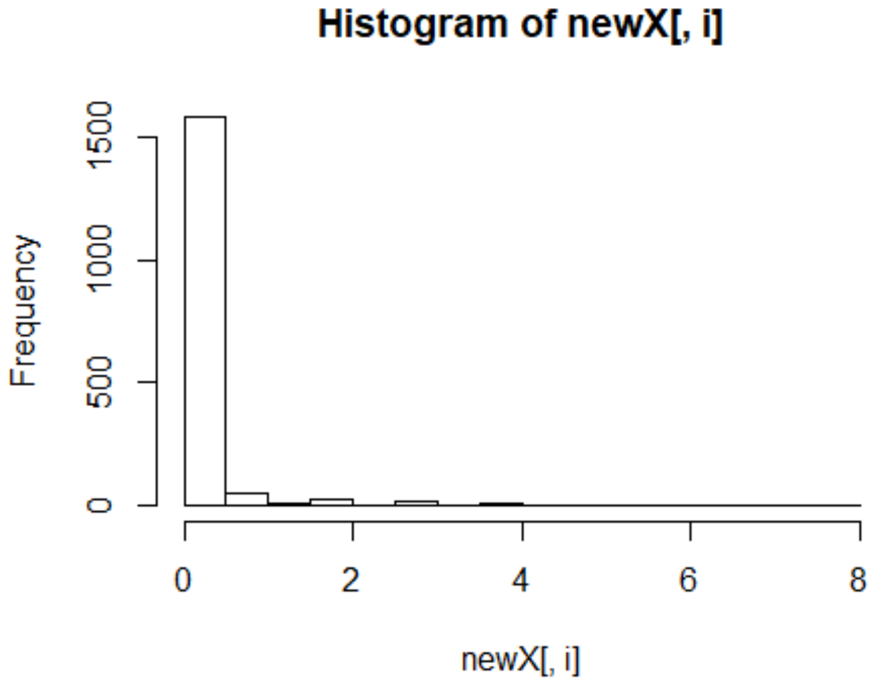
Histogram of newX[, i]
Frequency
0
500
1000
1500
0
2
4
6
8
newX[, i]

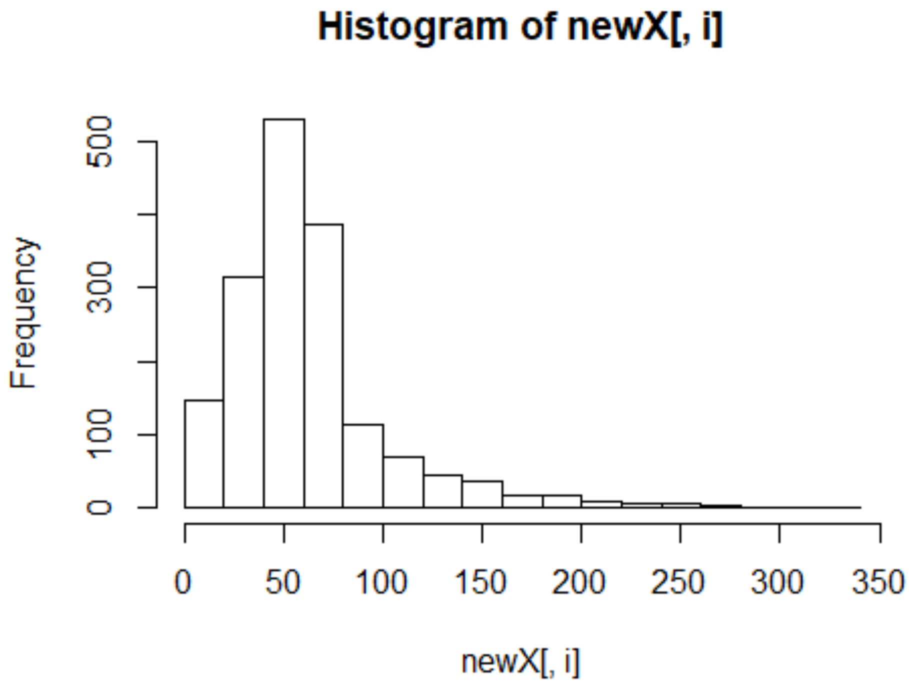
Histogram of newX[, i]
Frequency
0
100
300
500
0
50
100
150
200
250
300
350
newX[, i]

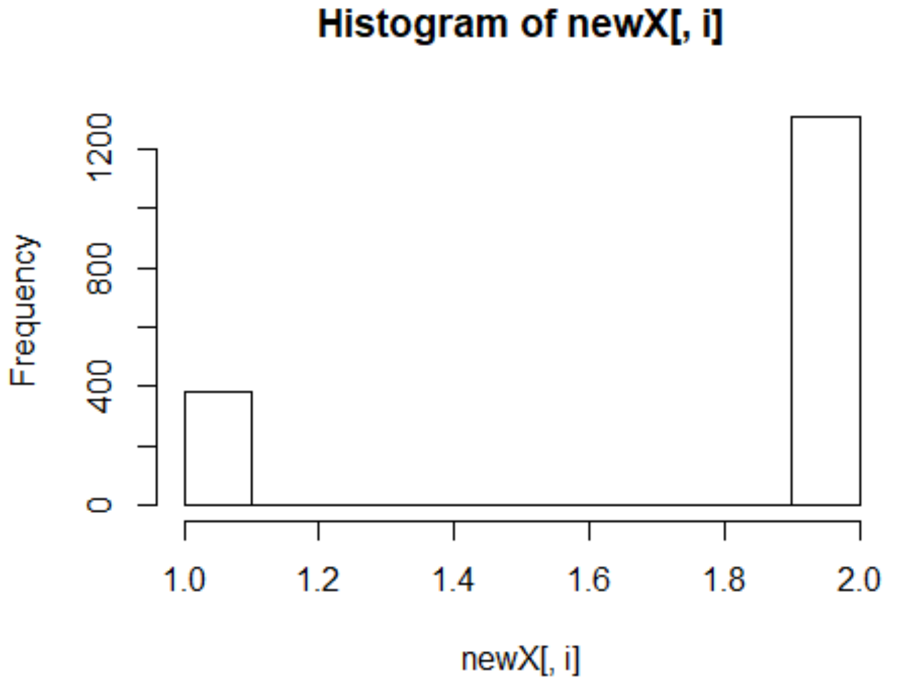
Histogram of newX[, i]
Frequency
0
400
800
1200
1.0
1.2
1.4
1.6
1.8
2.0
newX[, i]

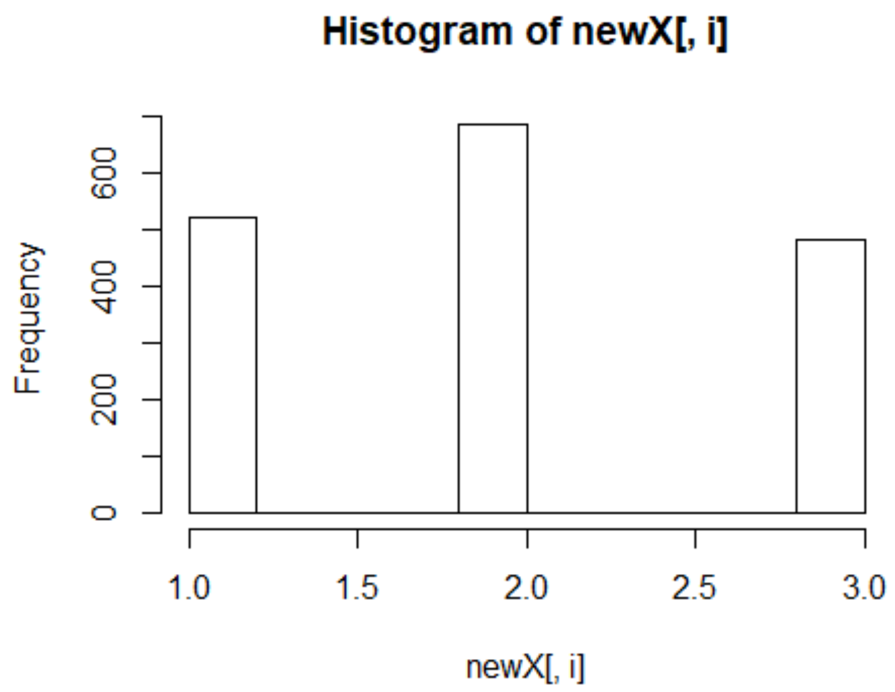

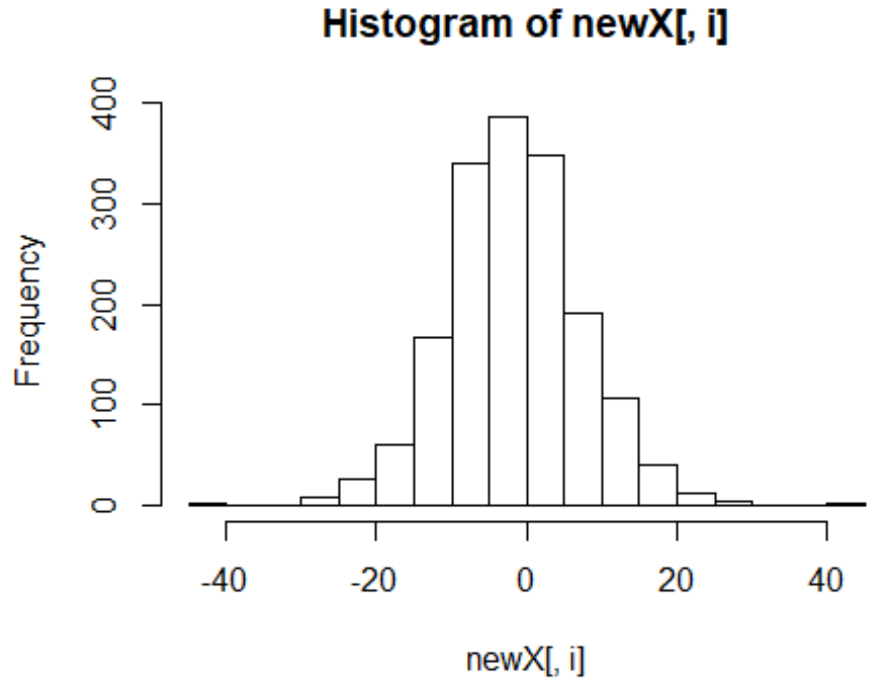


```
# round the children variable (count variable instead of continuous)
DataNetwork[,5] <- round(DataNetwork[,5])

summary(DataNetwork) # old dataset
##      age_OLD        height_OLD      weight_OLD         hc_OLD
##  Min.   :18.00   Min.   :145.0   Min.   : 41.00   Min.   :1.000
##  1st Qu.:22.00   1st Qu.:162.0   1st Qu.: 55.00   1st Qu.:1.000
```

```
##  Median :25.00   Median :166.0   Median : 61.00   Median :1.000  
##  Mean   :25.47   Mean   :166.1   Mean   : 63.45   Mean   :1.459  
##  3rd Qu.:29.00   3rd Qu.:170.0   3rd Qu.: 68.09   3rd Qu.:2.000  
##  Max.   :45.00   Max.   :196.2   Max.   :115.00   Max.   :2.000  
##  biolchild_OLD     FSFIdes_OLD     FSFIaro_OLD     FSFIlub_OLD   
##  Min.   :0.0000   Min.   :1.200   Min.   :0.000   Min.   :0.000  
##  1st Qu.:0.0000   1st Qu.:3.000   1st Qu.:4.200   1st Qu.:4.800  
##  Median :0.0000   Median :3.000   Median :5.100   Median :5.700  
##  Mean   :0.5296   Mean   :3.256   Mean   :4.482   Mean   :4.927  
##  3rd Qu.:1.0000   3rd Qu.:3.600   3rd Qu.:5.700   3rd Qu.:6.000  
##  Max.   :9.0000   Max.   :5.400   Max.   :6.000   Max.   :7.438  
##   FSFIorg_OLD     FSFIsat_OLD     FSFIpain_OLD      SDS_OLD     
##  Min.   :0.000   Min.   :0.281   Min.   :0.000   Min.   : 0.000  
##  1st Qu.:2.800   1st Qu.:2.800   1st Qu.:3.600   1st Qu.: 3.000  
##  Median :4.788   Median :4.800   Median :5.400   Median : 7.000  
##  Mean   :3.964   Mean   :4.149   Mean   :4.229   Mean   : 7.502  
##  3rd Qu.:5.600   3rd Qu.:5.600   3rd Qu.:6.000   3rd Qu.:11.000  
##  Max.   :6.306   Max.   :6.840   Max.   :6.412   Max.   :28.000  
##    BSIdep_OLD       BSIanx_OLD         BI_OLD        AUDIT_OLD      
##  Min.   : 0.000   Min.   : 0.000   Min.   :11.00   Min.   : 0.7428  
##  1st Qu.: 2.000   1st Qu.: 1.000   1st Qu.:22.00   1st Qu.: 4.0000  
##  Median : 4.000   Median : 3.000   Median :26.00   Median : 6.0000  
##  Mean   : 5.375   Mean   : 3.884   Mean   :26.42   Mean   : 7.1840  
##  3rd Qu.: 8.000   3rd Qu.: 5.000   3rd Qu.:31.00   3rd Qu.: 9.0000  
##  Max.   :24.000   Max.   :23.000   Max.   :45.06   Max.   :31.0000  
##  DSFItoolittle_OLD DSFItoomuch_OLD     SOI_OLD       haspartner_OLD 
##  Min.   : 0.00     Min.   :0.0000   Min.   :  6.00   Min.   :1.000  
##  1st Qu.: 1.00     1st Qu.:0.0000   1st Qu.: 38.01   1st Qu.:2.000  
##  Median : 3.00     Median :0.0000   Median : 56.00   Median :2.000  
##  Mean   : 4.58     Mean   :0.1224   Mean   : 62.36   Mean   :1.774  
##  3rd Qu.: 7.00     3rd Qu.:0.0000   3rd Qu.: 73.00   3rd Qu.:2.000  
##  Max.   :29.00     Max.   :7.6294   Max.   :335.68   Max.   :2.000  
##    SDI_groups      SDI_change     
##  Min.   :1.000   Min.   :-40.025  
##  1st Qu.:1.000   1st Qu.: -7.000  
##  Median :2.000   Median : -1.000  
##  Mean   :1.977   Mean   : -1.092  
##  3rd Qu.:3.000   3rd Qu.:  4.000  
##  Max.   :3.000   Max.   : 41.000

describe(DataNetwork) # old dataset
##                   vars    n   mean    sd median trimmed   mad    min
## age_OLD              1 1692  25.47  4.87  25.00   25.16  5.19  18.00
## height_OLD           2 1692 166.07  6.22 166.00  165.98  5.93 145.00
## weight_OLD           3 1692  63.45 11.46  61.00   62.14  8.90  41.00
## hc_OLD               4 1692   1.46  0.50   1.00    1.45  0.00   1.00
## biolchild_OLD        5 1692   0.53  0.98   0.00    0.31  0.00   0.00
## FSFIdes_OLD          6 1692   3.26  0.91   3.00    3.25  0.89   1.20
## FSFIaro_OLD          7 1692   4.48  1.70   5.10    4.83  0.89   0.00
## FSFIlub_OLD          8 1692   4.93  1.83   5.70    5.41  0.44   0.00
## FSFIorg_OLD          9 1692   3.96  1.94   4.79    4.20  1.80   0.00
## FSFIsat_OLD         10 1692   4.15  1.83   4.80    4.36  1.78   0.28
## FSFIpain_OLD        11 1692   4.23  2.31   5.40    4.54  0.89   0.00
## SDS_OLD             12 1692   7.50  5.52   7.00    7.03  5.93   0.00
## BSIdep_OLD          13 1692   5.38  4.55   4.00    4.77  2.97   0.00
## BSIanx_OLD          14 1692   3.88  4.00   3.00    3.19  2.97   0.00
## BI_OLD              15 1692  26.42  6.53  26.00   26.30  7.41  11.00
## AUDIT_OLD           16 1692   7.18  4.26   6.00    6.61  2.97   0.74
## DSFItoolittle_OLD   17 1692   4.58  4.83   3.00    3.83  4.45   0.00
## DSFItoomuch_OLD     18 1692   0.12  0.57   0.00    0.00  0.00   0.00
## SOI_OLD             19 1692  62.36 39.13  56.00   56.80 25.20   6.00
## haspartner_OLD      20 1692   1.77  0.42   2.00    1.84  0.00   1.00
## SDI_groups          21 1692   1.98  0.77   2.00    1.97  1.48   1.00
```

```
## SDI_change          22 1692  -1.09  8.74  -1.00   -1.21  8.90 -40.02
##                      max  range  skew kurtosis   se
## age_OLD             45.00  27.00  0.70     0.54 0.12
## height_OLD         196.20  51.20  0.17     0.46 0.15
## weight_OLD         115.00  74.00  1.25     2.16 0.28
## hc_OLD               2.00   1.00  0.17    -1.97 0.01
## biolchild_OLD        9.00   9.00  2.33     7.28 0.02
## FSFIdes_OLD          5.40   4.20  0.07    -0.23 0.02
## FSFIaro_OLD          6.00   6.00 -1.59     1.56 0.04
## FSFIlub_OLD          7.44   7.44 -1.98     2.55 0.04
## FSFIorg_OLD          6.31   6.31 -0.79    -0.59 0.05
## FSFIsat_OLD          6.84   6.56 -0.80    -0.74 0.04
## FSFIpain_OLD         6.41   6.41 -1.05    -0.56 0.06
## SDS_OLD             28.00  28.00  0.71     0.01 0.13
## BSIdep_OLD          24.00  24.00  1.22     1.26 0.11
## BSIanx_OLD          23.00  23.00  1.85     3.95 0.10
## BI_OLD              45.06  34.06  0.16    -0.49 0.16
## AUDIT_OLD           31.00  30.26  1.33     2.14 0.10
## DSFItoolittle_OLD  29.00  29.00  1.26     1.22 0.12
## DSFItoomuch_OLD      7.63   7.63  6.37    50.41 0.01
## SOI_OLD            335.68 329.68  1.95     5.79 0.95
## haspartner_OLD       2.00   1.00 -1.31    -0.29 0.01
## SDI_groups           3.00   2.00  0.04    -1.32 0.02
## SDI_change          41.00  81.02  0.13     0.55 0.21


##########################
# Subset Data into Groups#
##########################
# double checked that 1 = decrease 2 = stable 3 = increase
Decrease <- subset(DataNetwork, SDI_groups==1, select=age_OLD:haspartner_OLD)
Stable <- subset(DataNetwork, SDI_groups==2, select=age_OLD:haspartner_OLD)
Increase <- subset(DataNetwork, SDI_groups==3, select=age_OLD:haspartner_OLD)

nrow(Decrease) + nrow(Stable) + nrow(Increase) # check whether everyone has been assigned
## [1] 1692

# descriptives per group
describe(Decrease)
##                   vars   n   mean    sd median trimmed   mad    min    max
## age_OLD              1 522  25.63  4.86  25.00   25.31  4.45  18.00  43.00
## height_OLD           2 522 166.09  6.51 166.00  166.00  5.93 145.00 196.20
## weight_OLD           3 522  63.51 11.46  61.00   62.20 10.38  41.00 110.00
## hc_OLD               4 522   1.44  0.50   1.00    1.42  0.00   1.00   2.00
## biolchild_OLD        5 522   0.51  0.95   0.00    0.29  0.00   0.00   6.00
## FSFIdes_OLD          6 522   3.46  0.88   3.60    3.46  0.89   1.20   5.40
## FSFIaro_OLD          7 522   4.72  1.56   5.40    5.07  0.89   0.00   6.00
## FSFIlub_OLD          8 522   5.15  1.63   6.00    5.60  0.00   0.00   6.00
## FSFIorg_OLD          9 522   4.15  1.85   4.80    4.40  1.78   0.00   6.00
## FSFIsat_OLD         10 522   4.20  1.81   4.80    4.43  1.78   0.28   6.84
## FSFIpain_OLD        11 522   4.34  2.29   5.40    4.67  0.89   0.00   6.05
## SDS_OLD             12 522   7.40  5.38   7.00    6.98  5.93   0.00  26.00
## BSIdep_OLD          13 522   5.34  4.59   4.00    4.68  2.97   0.00  23.00
## BSIanx_OLD          14 522   3.73  3.79   2.14    3.05  2.75   0.00  22.00
## BI_OLD              15 522  26.08  6.52  26.00   25.93  7.41  11.00  44.00
## AUDIT_OLD           16 522   7.76  4.46   7.00    7.16  4.45   1.02  26.00
## DSFItoolittle_OLD   17 522   4.99  4.88   4.00    4.34  4.45   0.00  26.00
## DSFItoomuch_OLD     18 522   0.10  0.48   0.00    0.00  0.00   0.00   4.00
## SOI_OLD             19 522  67.96 41.51  59.00   62.06 25.57   7.00 277.86
## haspartner_OLD      20 522   1.77  0.42   2.00    1.83  0.00   1.00   2.00
##                    range  skew kurtosis   se
## age_OLD            25.00  0.68     0.36 0.21
## height_OLD         51.20  0.17     0.55 0.28
```

```
## weight_OLD          69.00  1.23     2.05 0.50
## hc_OLD               1.00  0.25    -1.94 0.02
## biolchild_OLD        6.00  2.32     6.45 0.04
## FSFIdes_OLD          4.20 -0.04    -0.18 0.04
## FSFIaro_OLD          6.00 -1.90     2.98 0.07
## FSFIlub_OLD          6.00 -2.46     4.93 0.07
## FSFIorg_OLD          6.00 -0.92    -0.27 0.08
## FSFIsat_OLD          6.56 -0.85    -0.60 0.08
## FSFIpain_OLD         6.05 -1.15    -0.35 0.10
## SDS_OLD             26.00  0.63    -0.26 0.24
## BSIdep_OLD          23.00  1.33     1.54 0.20
## BSIanx_OLD          22.00  1.76     3.32 0.17
## BI_OLD              33.00  0.18    -0.49 0.29
## AUDIT_OLD           24.98  1.25     1.58 0.20
## DSFItoolittle_OLD   26.00  1.08     0.75 0.21
## DSFItoomuch_OLD      4.00  5.48    32.58 0.02
## SOI_OLD            270.86  1.71     3.94 1.82
## haspartner_OLD       1.00 -1.25    -0.43 0.02
```

```
describe(Stable)
```

```
##                   vars   n   mean    sd median trimmed   mad    min    max
## age_OLD              1 687  25.30  4.82   25.0   24.97  4.45  18.00  45.00
## height_OLD           2 687 165.99  6.03  166.0  165.85  5.93 145.00 195.00
## weight_OLD           3 687  63.16 11.24   61.0   61.91  8.90  42.00 113.00
## hc_OLD               4 687   1.47  0.50    1.0    1.46  0.00   1.00   2.00
## biolchild_OLD        5 687   0.52  0.95    0.0    0.31  0.00   0.00   6.00
## FSFIdes_OLD          6 687   3.21  0.89    3.0    3.20  0.89   1.20   5.40
## FSFIaro_OLD          7 687   4.41  1.70    5.1    4.73  0.89   0.00   6.00
## FSFIlub_OLD          8 687   4.85  1.86    5.7    5.31  0.44   0.00   6.13
## FSFIorg_OLD          9 687   3.89  1.92    4.4    4.11  1.78   0.00   6.00
## FSFIsat_OLD         10 687   4.09  1.84    4.8    4.28  1.78   0.40   6.00
## FSFIpain_OLD        11 687   4.12  2.34    5.4    4.40  0.89   0.00   6.41
## SDS_OLD             12 687   7.51  5.45    7.0    7.04  5.93   0.00  28.00
## BSIdep_OLD          13 687   5.21  4.32    4.0    4.66  2.97   0.00  22.00
## BSIanx_OLD          14 687   3.87  3.98    3.0    3.19  2.97   0.00  23.00
## BI_OLD              15 687  26.49  6.29   26.0   26.35  7.01  11.00  45.00
## AUDIT_OLD           16 687   7.00  4.09    6.0    6.49  3.67   0.93  29.00
## DSFItoolittle_OLD   17 687   4.59  5.02    3.0    3.77  4.45   0.00  22.00
## DSFItoomuch_OLD     18 687   0.11  0.51    0.0    0.00  0.00   0.00   6.00
## SOI_OLD             19 687  59.28 36.79   56.0   54.44 23.72   7.00 335.68
## haspartner_OLD      20 687   1.76  0.43    2.0    1.82  0.00   1.00   2.00
##                    range  skew kurtosis   se
## age_OLD            27.00  0.76     0.78 0.18
## height_OLD         50.00  0.23     0.57 0.23
## weight_OLD         71.00  1.20     2.00 0.43
## hc_OLD              1.00  0.12    -1.99 0.02
## biolchild_OLD       6.00  2.08     4.64 0.04
## FSFIdes_OLD         4.20  0.13    -0.22 0.03
## FSFIaro_OLD         6.00 -1.54     1.39 0.06
## FSFIlub_OLD         6.13 -1.88     2.15 0.07
## FSFIorg_OLD         6.00 -0.75    -0.60 0.07
## FSFIsat_OLD         5.60 -0.76    -0.81 0.07
## FSFIpain_OLD        6.41 -0.96    -0.73 0.09
## SDS_OLD            28.00  0.75     0.18 0.21
## BSIdep_OLD         22.00  1.18     1.25 0.16
## BSIanx_OLD         23.00  1.92     4.42 0.15
## BI_OLD             34.00  0.22    -0.49 0.24
## AUDIT_OLD          28.07  1.21     1.77 0.16
## DSFItoolittle_OLD  22.00  1.24     0.73 0.19
## DSFItoomuch_OLD     6.00  6.12    45.88 0.02
## SOI_OLD           328.68  2.39     9.66 1.40
## haspartner_OLD      1.00 -1.20    -0.57 0.02
```

```
describe(Increase)
##                    vars   n   mean    sd median trimmed   mad    min    max
## age_OLD               1 483  25.54  4.94  25.00   25.25  5.93  18.00  44.00
## height_OLD            2 483 166.18  6.18 166.00  166.13  5.93 146.00 186.00
## weight_OLD            3 483  63.81 11.77  62.00   62.41  8.90  42.00 115.00
## hc_OLD                4 483   1.46  0.50   1.00    1.45  0.00   1.00   2.00
## biolchild_OLD         5 483   0.57  1.05   0.00    0.33  0.00   0.00   9.00
## FSFIdes_OLD           6 483   3.10  0.92   3.00    3.08  0.89   1.20   5.40
## FSFIaro_OLD           7 483   4.33  1.82   5.10    4.66  0.89   0.00   6.00
## FSFIlub_OLD           8 483   4.79  1.98   5.70    5.23  0.44   0.00   7.44
## FSFIorg_OLD           9 483   3.87  2.04   4.80    4.09  1.78   0.00   6.31
## FSFIsat_OLD          10 483   4.18  1.85   4.80    4.38  1.78   0.40   6.00
## FSFIpain_OLD         11 483   4.27  2.31   5.40    4.58  0.89   0.00   6.21
## SDS_OLD              12 483   7.60  5.79   7.00    7.10  5.93   0.00  27.00
## BSIdep_OLD           13 483   5.65  4.83   4.00    5.03  4.45   0.00  24.00
## BSIanx_OLD           14 483   4.08  4.24   3.00    3.34  2.97   0.00  23.00
## BI_OLD               15 483  26.67  6.85  27.00   26.62  7.41  11.00  45.06
## AUDIT_OLD            16 483   6.82  4.24   6.00    6.20  2.97   0.74  31.00
## DSFItoolittle_OLD    17 483   4.12  4.47   3.00    3.41  4.45   0.00  29.00
## DSFItoomuch_OLD      18 483   0.16  0.72   0.00    0.00  0.00   0.00   7.63
## SOI_OLD              19 483  60.69 39.16  55.75   55.08 27.80   6.00 249.00
## haspartner_OLD       20 483   1.81  0.40   2.00    1.88  0.00   1.00   2.00
##                     range  skew kurtosis   se
## age_OLD             26.00  0.65     0.38 0.22
## height_OLD          40.00  0.08     0.12 0.28
## weight_OLD          73.00  1.32     2.38 0.54
## hc_OLD               1.00  0.14    -1.98 0.02
## biolchild_OLD        9.00  2.54     9.95 0.05
## FSFIdes_OLD          4.20  0.16    -0.20 0.04
## FSFIaro_OLD          6.00 -1.37     0.72 0.08
## FSFIlub_OLD          7.44 -1.72     1.43 0.09
## FSFIorg_OLD          6.31 -0.70    -0.88 0.09
## FSFIsat_OLD          5.60 -0.80    -0.81 0.08
## FSFIpain_OLD         6.21 -1.06    -0.53 0.11
## SDS_OLD             27.00  0.72    -0.03 0.26
## BSIdep_OLD          24.00  1.12     0.85 0.22
## BSIanx_OLD          23.00  1.80     3.60 0.19
## BI_OLD              34.06  0.07    -0.53 0.31
## AUDIT_OLD           30.26  1.58     3.27 0.19
## DSFItoolittle_OLD   29.00  1.52     2.96 0.20
## DSFItoomuch_OLD      7.63  6.15    44.68 0.03
## SOI_OLD            243.00  1.70     3.92 1.78
## haspartner_OLD       1.00 -1.54     0.37 0.02

##################################
# Random Subsamples of same size #
##################################

set.seed(17) # to make sure we always get the same random sample
nrow(Decrease)
## [1] 522
nrow(Stable)
## [1] 687
nrow(Increase) # 483
## [1] 483
randomSample = function(df,n) {
  return (df[sample(nrow(df), n),])
}

DecreaseRS <- randomSample(Decrease, n = 483)
StableRS <- randomSample(Stable, n = 483)
IncreaseRS <- Increase
```

```
describe(DecreaseRS)
##                    vars   n   mean    sd median trimmed   mad    min    max
## age_OLD               1 483  25.67  4.84   25.0   25.35  4.45  18.00  43.00
## height_OLD            2 483 166.20  6.44  166.0  166.10  5.93 147.00 196.20
## weight_OLD            3 483  63.58 11.33   62.0   62.29 10.38  41.00 110.00
## hc_OLD                4 483   1.43  0.50    1.0    1.42  0.00   1.00   2.00
## biolchild_OLD         5 483   0.50  0.96    0.0    0.29  0.00   0.00   6.00
## FSFIdes_OLD           6 483   3.45  0.87    3.6    3.45  0.89   1.20   5.40
## FSFIaro_OLD           7 483   4.74  1.56    5.4    5.09  0.89   0.00   6.00
## FSFIlub_OLD           8 483   5.16  1.63    6.0    5.62  0.00   0.00   6.00
## FSFIorg_OLD           9 483   4.18  1.84    4.8    4.45  1.78   0.00   6.00
## FSFIsat_OLD          10 483   4.20  1.81    4.8    4.43  1.78   0.28   6.84
## FSFIpain_OLD         11 483   4.32  2.29    5.4    4.65  0.89   0.00   6.05
## SDS_OLD              12 483   7.34  5.30    7.0    6.92  5.93   0.00  26.00
## BSIdep_OLD           13 483   5.35  4.59    4.0    4.69  2.97   0.00  23.00
## BSIanx_OLD           14 483   3.67  3.71    2.0    3.02  2.97   0.00  22.00
## BI_OLD               15 483  26.04  6.48   26.0   25.88  7.41  11.00  44.00
## AUDIT_OLD            16 483   7.74  4.45    7.0    7.15  4.45   1.02  26.00
## DSFItoolittle_OLD    17 483   5.07  4.91    4.0    4.42  4.45   0.00  26.00
## DSFItoomuch_OLD      18 483   0.10  0.47    0.0    0.00  0.00   0.00   4.00
## SOI_OLD              19 483  68.38 42.17   59.0   62.36 26.69   7.00 277.86
## haspartner_OLD       20 483   1.76  0.43    2.0    1.83  0.00   1.00   2.00
##                     range  skew kurtosis   se
## age_OLD             25.00  0.67     0.34 0.22
## height_OLD          49.20  0.23     0.57 0.29
## weight_OLD          69.00  1.25     2.19 0.52
## hc_OLD               1.00  0.26    -1.94 0.02
## biolchild_OLD        6.00  2.38     6.81 0.04
## FSFIdes_OLD          4.20  0.00    -0.23 0.04
## FSFIaro_OLD          6.00 -1.94     3.14 0.07
## FSFIlub_OLD          6.00 -2.46     4.93 0.07
## FSFIorg_OLD          6.00 -0.97    -0.16 0.08
## FSFIsat_OLD          6.56 -0.85    -0.61 0.08
## FSFIpain_OLD         6.05 -1.13    -0.40 0.10
## SDS_OLD             26.00  0.66    -0.15 0.24
## BSIdep_OLD          23.00  1.35     1.69 0.21
## BSIanx_OLD          22.00  1.77     3.42 0.17
## BI_OLD              33.00  0.20    -0.41 0.30
## AUDIT_OLD           24.98  1.29     1.72 0.20
## DSFItoolittle_OLD   26.00  1.07     0.73 0.22
## DSFItoomuch_OLD      4.00  5.50    33.08 0.02
## SOI_OLD            270.86  1.69     3.81 1.92
## haspartner_OLD       1.00 -1.23    -0.50 0.02

describe(StableRS)
##                    vars   n   mean    sd median trimmed   mad    min    max
## age_OLD               1 483  25.18  4.73  25.00   24.89  4.45  18.00  43.00
## height_OLD            2 483 165.93  6.13 165.02  165.69  5.96 149.00 195.00
## weight_OLD            3 483  63.21 11.11  61.00   61.92  8.90  44.00 113.00
## hc_OLD                4 483   1.47  0.50   1.00    1.46  0.00   1.00   2.00
## biolchild_OLD         5 483   0.52  0.96   0.00    0.31  0.00   0.00   6.00
## FSFIdes_OLD           6 483   3.25  0.92   3.00    3.24  0.89   1.20   5.40
## FSFIaro_OLD           7 483   4.39  1.74   5.10    4.73  0.89   0.00   6.00
## FSFIlub_OLD           8 483   4.84  1.88   5.70    5.29  0.44   0.00   6.13
## FSFIorg_OLD           9 483   3.85  1.93   4.40    4.06  1.78   0.00   6.00
## FSFIsat_OLD          10 483   4.10  1.84   4.80    4.29  1.78   0.40   6.00
## FSFIpain_OLD         11 483   4.14  2.32   5.40    4.42  0.89   0.00   6.41
## SDS_OLD              12 483   7.44  5.48   7.00    6.95  5.93   0.00  28.00
## BSIdep_OLD           13 483   5.29  4.45   4.00    4.74  4.45   0.00  22.00
## BSIanx_OLD           14 483   3.98  4.15   3.00    3.27  2.97   0.00  23.00
## BI_OLD               15 483  26.50  6.24  26.00   26.36  5.93  11.00  44.00
## AUDIT_OLD            16 483   7.16  4.12   6.00    6.65  3.61   0.93  22.00
## DSFItoolittle_OLD    17 483   4.60  5.03   3.00    3.76  4.45   0.00  22.00
```

```
## DSFItoomuch_OLD     18 483   0.09  0.46   0.00    0.00  0.00   0.00   4.00
## SOI_OLD             19 483  60.81 37.68  56.00   55.68 23.72   7.00 335.68
## haspartner_OLD      20 483   1.76  0.43   2.00    1.82  0.00   1.00   2.00
##                    range  skew kurtosis   se
## age_OLD            25.00  0.63     0.41 0.22
## height_OLD         46.00  0.42     0.65 0.28
## weight_OLD         69.00  1.29     2.34 0.51
## hc_OLD              1.00  0.12    -1.99 0.02
## biolchild_OLD       6.00  2.17     5.33 0.04
## FSFIdes_OLD         4.20  0.10    -0.31 0.04
## FSFIaro_OLD         6.00 -1.51     1.18 0.08
## FSFIlub_OLD         6.13 -1.85     1.99 0.09
## FSFIorg_OLD         6.00 -0.73    -0.63 0.09
## FSFIsat_OLD         5.60 -0.76    -0.78 0.08
## FSFIpain_OLD        6.41 -0.98    -0.69 0.11
## SDS_OLD            28.00  0.83     0.49 0.25
## BSIdep_OLD         22.00  1.14     1.06 0.20
## BSIanx_OLD         23.00  1.90     4.25 0.19
## BI_OLD             33.00  0.22    -0.45 0.28
## AUDIT_OLD          21.07  1.02     0.61 0.19
## DSFItoolittle_OLD  22.00  1.25     0.75 0.23
## DSFItoomuch_OLD     4.00  6.04    40.15 0.02
## SOI_OLD           328.68  2.36     9.52 1.71
## haspartner_OLD      1.00 -1.20    -0.56 0.02

describe(IncreaseRS)
##                   vars   n   mean    sd median trimmed   mad    min    max
## age_OLD              1 483  25.54  4.94  25.00   25.25  5.93  18.00  44.00
## height_OLD           2 483 166.18  6.18 166.00  166.13  5.93 146.00 186.00
## weight_OLD           3 483  63.81 11.77  62.00   62.41  8.90  42.00 115.00
## hc_OLD               4 483   1.46  0.50   1.00    1.45  0.00   1.00   2.00
## biolchild_OLD        5 483   0.57  1.05   0.00    0.33  0.00   0.00   9.00
## FSFIdes_OLD          6 483   3.10  0.92   3.00    3.08  0.89   1.20   5.40
## FSFIaro_OLD          7 483   4.33  1.82   5.10    4.66  0.89   0.00   6.00
## FSFIlub_OLD          8 483   4.79  1.98   5.70    5.23  0.44   0.00   7.44
## FSFIorg_OLD          9 483   3.87  2.04   4.80    4.09  1.78   0.00   6.31
## FSFIsat_OLD         10 483   4.18  1.85   4.80    4.38  1.78   0.40   6.00
## FSFIpain_OLD        11 483   4.27  2.31   5.40    4.58  0.89   0.00   6.21
## SDS_OLD             12 483   7.60  5.79   7.00    7.10  5.93   0.00  27.00
## BSIdep_OLD          13 483   5.65  4.83   4.00    5.03  4.45   0.00  24.00
## BSIanx_OLD          14 483   4.08  4.24   3.00    3.34  2.97   0.00  23.00
## BI_OLD              15 483  26.67  6.85  27.00   26.62  7.41  11.00  45.06
## AUDIT_OLD           16 483   6.82  4.24   6.00    6.20  2.97   0.74  31.00
## DSFItoolittle_OLD   17 483   4.12  4.47   3.00    3.41  4.45   0.00  29.00
## DSFItoomuch_OLD     18 483   0.16  0.72   0.00    0.00  0.00   0.00   7.63
## SOI_OLD             19 483  60.69 39.16  55.75   55.08 27.80   6.00 249.00
## haspartner_OLD      20 483   1.81  0.40   2.00    1.88  0.00   1.00   2.00
##                    range  skew kurtosis   se
## age_OLD            26.00  0.65     0.38 0.22
## height_OLD         40.00  0.08     0.12 0.28
## weight_OLD         73.00  1.32     2.38 0.54
## hc_OLD              1.00  0.14    -1.98 0.02
## biolchild_OLD       9.00  2.54     9.95 0.05
## FSFIdes_OLD         4.20  0.16    -0.20 0.04
## FSFIaro_OLD         6.00 -1.37     0.72 0.08
## FSFIlub_OLD         7.44 -1.72     1.43 0.09
## FSFIorg_OLD         6.31 -0.70    -0.88 0.09
## FSFIsat_OLD         5.60 -0.80    -0.81 0.08
## FSFIpain_OLD        6.21 -1.06    -0.53 0.11
## SDS_OLD            27.00  0.72    -0.03 0.26
## BSIdep_OLD         24.00  1.12     0.85 0.22
## BSIanx_OLD         23.00  1.80     3.60 0.19
## BI_OLD             34.06  0.07    -0.53 0.31
```

```
## AUDIT_OLD           30.26  1.58      3.27 0.19
## DSFItoolittle_OLD   29.00  1.52      2.96 0.20
## DSFItoomuch_OLD      7.63  6.15     44.68 0.03
## SOI_OLD            243.00  1.70      3.92 1.78
## haspartner_OLD       1.00 -1.54      0.37 0.02

table(DecreaseRS[,20])
##
##   1   2
## 115 368
table(DecreaseRS[,20])[1]/483
##         1
## 0.2380952
table(DecreaseRS[,20])[2]/483
##         2
## 0.7619048

table(StableRS[,20])
##
##   1   2
## 117 366
table(StableRS[,20])[1]/483
##        1
## 0.242236
table(StableRS[,20])[2]/483
##        2
## 0.757764

table(IncreaseRS[,20])
##
##   1   2
##  94 389
table(IncreaseRS[,20])[1]/483
##        1
## 0.194617
table(IncreaseRS[,20])[2]/483
##        2
## 0.805383
```

4. Network Estimation including Hormonal Contraception

```
#########################
# 4. Network estimation #
#########################
colnames(DataNetwork)
##  [1] "age_OLD"           "height_OLD"        "weight_OLD"
##  [4] "hc_OLD"            "biolchild_OLD"     "FSFIdes_OLD"
##  [7] "FSFIaro_OLD"       "FSFIlub_OLD"       "FSFIorg_OLD"
## [10] "FSFIsat_OLD"       "FSFIpain_OLD"      "SDS_OLD"
## [13] "BSIdep_OLD"        "BSIanx_OLD"        "BI_OLD"
## [16] "AUDIT_OLD"         "DSFItoolittle_OLD" "DSFItoomuch_OLD"
## [19] "SOI_OLD"           "haspartner_OLD"    "SDI_groups"
## [22] "SDI_change"

VarDomain <- c("g","g","g","c",
               "p","g","g",
               "g","g","g",
               "g","g","g",
               "g","g","g",
               "g","g","g",
               "c")
```

```
VarLevel <- c(1,1,1,2,
              1,1,1,
              1,1,1,
              1,1,1,
              1,1,1,
              1,1,1,
              2)

library(mgm)

fit_Decrease <- mgm(data = as.matrix(DecreaseRS),
                    type = VarDomain,
                    level = VarLevel,
                    k = 2,
                    lambdaSel = 'EBIC',
                    lambdaGam = 0.5) # hyper parameter

fit_Stable <- mgm(data = as.matrix(StableRS),
                  type = VarDomain,
                  level = VarLevel,
                  k = 2,
                  lambdaSel = 'EBIC',
                  lambdaGam = 0.5) # hyper parameter

fit_Increase <- mgm(data = as.matrix(IncreaseRS),
                    type = VarDomain,
                    level = VarLevel,
                    k = 2,
                    lambdaSel = 'EBIC',
                    lambdaGam = 0.5) # hyper parameter

#######################
# Predictive Strength #
#######################

pred_Decrease <- predict(fit_Decrease,
                         DecreaseRS,
                         errorCon = 'R2') #save R2 into an object (varExpl = cont var)

pred_Stable <- predict(fit_Stable,
                       StableRS,
                       errorCon = 'R2') #save R2 into an object (varExpl = cont var)

pred_Increase <- predict(fit_Increase,
                         IncreaseRS,
                         errorCon = 'R2') #save R2 into an object (varExpl = cont var)

predR_Decrease <- pred_Decrease$errors$Error.R2
predR_Decrease <- as.numeric(as.character(predR_Decrease))

predR_Stable <- pred_Stable$errors$Error.R2
predR_Stable <- as.numeric(as.character(predR_Stable))

predR_Increase <- pred_Increase$errors$Error.R2
predR_Increase <- as.numeric(as.character(predR_Increase))

# set 'predictability' of categorical variables

predR_Decrease[4] <- pred_Decrease$errors$Error.CC[4]
predR_Stable[4] <- pred_Stable$errors$Error.CC[4]
predR_Increase[4] <- pred_Increase$errors$Error.CC[4]
predR_Decrease[20] <- pred_Decrease$errors$Error.CC[20]
predR_Stable[20] <- pred_Stable$errors$Error.CC[20]
```

```
predR_Increase[20] <- pred_Increase$errors$Error.CC[20]

mean(predR_Decrease, na.rm = TRUE) # mean variance explained across all nodes
## [1] 0.4168
sd(predR_Decrease, na.rm = TRUE)
## [1] 0.2517671

pred_Decrease$errors
##              Variable Error.R2 Error.CC Error.nCC CCmarg
## 1             age_OLD    0.255       NA        NA     NA
## 2          height_OLD    0.100       NA        NA     NA
## 3          weight_OLD    0.241       NA        NA     NA
## 4              hc_OLD       NA    0.631     0.152  0.565
## 5       biolchild_OLD    0.256       NA        NA     NA
## 6         FSFIdes_OLD    0.208       NA        NA     NA
## 7         FSFIaro_OLD    0.828       NA        NA     NA
## 8         FSFIlub_OLD    0.774       NA        NA     NA
## 9         FSFIorg_OLD    0.505       NA        NA     NA
## 10        FSFIsat_OLD    0.666       NA        NA     NA
## 11       FSFIpain_OLD    0.599       NA        NA     NA
## 12            SDS_OLD    0.286       NA        NA     NA
## 13         BSIdep_OLD    0.541       NA        NA     NA
## 14         BSIanx_OLD    0.449       NA        NA     NA
## 15             BI_OLD    0.326       NA        NA     NA
## 16          AUDIT_OLD    0.198       NA        NA     NA
## 17 DSFItoolittle_OLD    0.452       NA        NA     NA
## 18   DSFItoomuch_OLD    0.040       NA        NA     NA
## 19            SOI_OLD    0.111       NA        NA     NA
## 20      haspartner_OLD       NA    0.870     0.452  0.763

mean(predR_Stable, na.rm = TRUE) #mean variance explained across all nodes
## [1] 0.43065
sd(predR_Stable, na.rm = TRUE)
## [1] 0.2675724

pred_Stable$errors
##              Variable Error.R2 Error.CC Error.nCC CCmarg
## 1             age_OLD    0.300       NA        NA     NA
## 2          height_OLD    0.138       NA        NA     NA
## 3          weight_OLD    0.171       NA        NA     NA
## 4              hc_OLD       NA    0.530     0.000  0.530
## 5       biolchild_OLD    0.299       NA        NA     NA
## 6         FSFIdes_OLD    0.299       NA        NA     NA
## 7         FSFIaro_OLD    0.870       NA        NA     NA
## 8         FSFIlub_OLD    0.814       NA        NA     NA
## 9         FSFIorg_OLD    0.526       NA        NA     NA
## 10        FSFIsat_OLD    0.768       NA        NA     NA
## 11       FSFIpain_OLD    0.660       NA        NA     NA
## 12            SDS_OLD    0.261       NA        NA     NA
## 13         BSIdep_OLD    0.535       NA        NA     NA
## 14         BSIanx_OLD    0.420       NA        NA     NA
## 15             BI_OLD    0.163       NA        NA     NA
## 16          AUDIT_OLD    0.214       NA        NA     NA
## 17 DSFItoolittle_OLD    0.547       NA        NA     NA
## 18   DSFItoomuch_OLD    0.026       NA        NA     NA
## 19            SOI_OLD    0.167       NA        NA     NA
## 20      haspartner_OLD       NA    0.905     0.607  0.758

mean(predR_Increase, na.rm = TRUE) #mean variance explained across all nodes
## [1] 0.4575
sd(predR_Increase, na.rm = TRUE)
## [1] 0.267907
```

```
pred_Increase$errors
##             Variable Error.R2 Error.CC Error.nCC CCmarg
## 1            age_OLD    0.283       NA        NA     NA
## 2         height_OLD    0.166       NA        NA     NA
## 3         weight_OLD    0.208       NA        NA     NA
## 4             hc_OLD       NA    0.536     0.000  0.536 # nCC is the relative improvement
## 5      biolchild_OLD    0.315       NA        NA     NA
## 6        FSFIdes_OLD    0.336       NA        NA     NA
## 7        FSFIaro_OLD    0.880       NA        NA     NA
## 8        FSFIlub_OLD    0.850       NA        NA     NA
## 9        FSFIorg_OLD    0.595       NA        NA     NA
## 10       FSFIsat_OLD    0.779       NA        NA     NA
## 11      FSFIpain_OLD    0.657       NA        NA     NA
## 12           SDS_OLD    0.393       NA        NA     NA
## 13        BSIdep_OLD    0.587       NA        NA     NA
## 14        BSIanx_OLD    0.541       NA        NA     NA
## 15            BI_OLD    0.316       NA        NA     NA
## 16         AUDIT_OLD    0.201       NA        NA     NA
## 17 DSFItoolittle_OLD    0.426       NA        NA     NA
## 18   DSFItoomuch_OLD    0.031       NA        NA     NA
## 19           SOI_OLD    0.110       NA        NA     NA
## 20     haspartner_OLD       NA    0.940     0.691  0.806 # first is total last is intercept m
odel
```

5. Network Visualization including Hormonal Contraception

```
############################
# Network Layout Specifics #
############################

colNames <- c("Age", "Height", "Weight","HC", "Child", "Des", "Aro",
              "Lub", "Org", "Sat", "Pain", "Distr", "Dep", "Anx",
              "Body", "Alc", "Little", "Much", "SO", "Rel")

nodeDescr <- c("", "", "","", "Number of children", "Desire",
               "Arousal","Lubrication", "Orgasm", "Satisfaction", "Pain",
               "Sexually related personal distress", "Depression", "Anxiety",
               "Body image and dissatisfaction", "(Hazardous) Alcohol use",
               "Too little sexual sctivity", "Too much sexual activity",
               "Tendency/attitudes to(wards) uncommitted sexual relationships",
               "Relationship status (partnered or single)")

groupsNoHc <- list(Demographics = c(1:5,20),
                   Female_Sexual_Function_Index = c(6:11),
                   Sexual_Distress_Scale = c(12),
                   Brief_Symptom_Inventory = c(13:14),
                   Derogatis_Sexual_Function_Inventory = c(15),
                   Alcohol_Use_Disorders_Identification_Test = c(16),
                   Desired_and_Actual_Sexual_Activity_Scale = c(17:18),
                   Sociosexual_Orientation_Inventory = c(19))

nodeLabelColors <- c("white", "white", "white", "white","white",
                     "white","white","white","white","white","white",
                     "white",
                     "white","white",
                     "black","black","black","black","black",
                     "white")

#Plot Networks including hormonal contraception

library(qgraph)
qgraph(fit_Decrease$pairwise$wadj,
```

```
                    edge.color = fit_Decrease$pairwise$edgecolor,
                    layout = 'spring',
                    labels = colNames,
                    maximum = 1,
                    minimum = 0,
                    cut = 0.1,
                    details = TRUE,
                    vsize = 9,
                    label.cex = 1.2,
                    label.scale.equal = TRUE,
                    pie = predR_Decrease,
                    pieColor = "gray21",
                    pieBorder = 0.25,
                    groups = groupsNoHc,
                    nodeNames = nodeDescr,
                    legend = TRUE,
                    legend.mode = "style1",
                    legend.cex = 0.3,
                    palette = "gray",
                    negDashed = TRUE,
                    label.color = nodeLabelColors)
```

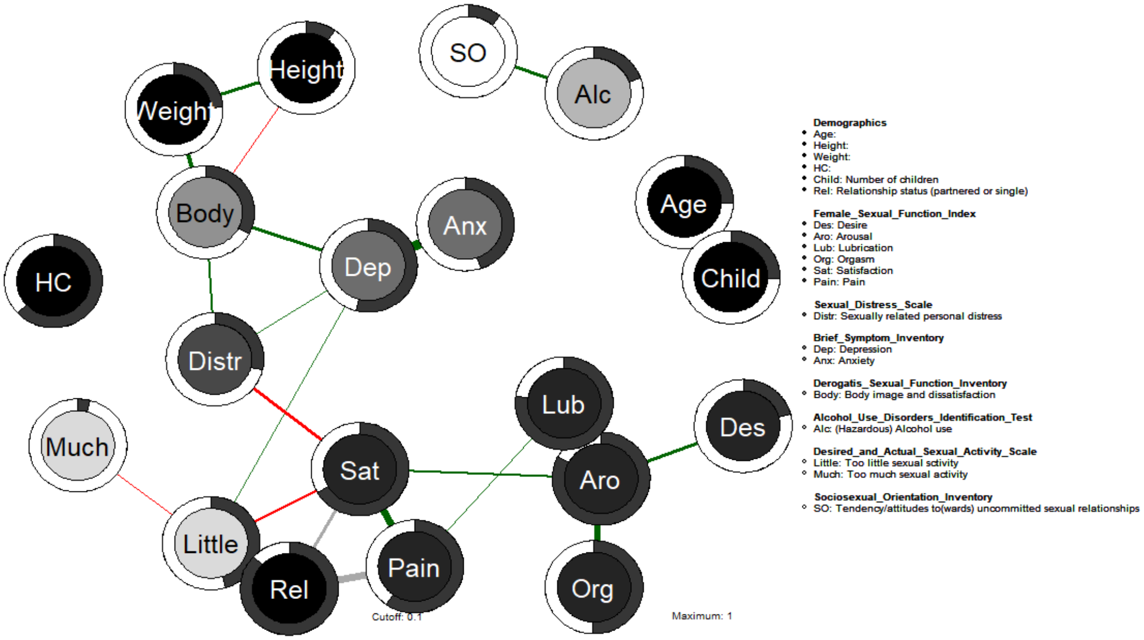


```
qgraph(fit_Stable$pairwise$wadj,
                  edge.color = fit_Stable$pairwise$edgecolor,
                  layout = 'spring',
                  labels = colNames,
                  maximum = 1,
                  minimum = 0,
                  cut = 0.1,
                  details = TRUE,
                  vsize = 9,
                  label.cex = 1.2,
                  label.scale.equal = TRUE,
                  pie = predR_Stable,
                  pieColor = "gray21",
                  pieBorder = 0.25,
                  groups = groupsNoHc,
                  nodeNames = nodeDescr,
                  legend = TRUE,
```

```r
                  legend.mode = "style1",
                  legend.cex = 0.3,
                  palette = "gray",
                  negDashed = TRUE,
                  label.color = nodeLabelColors)
```

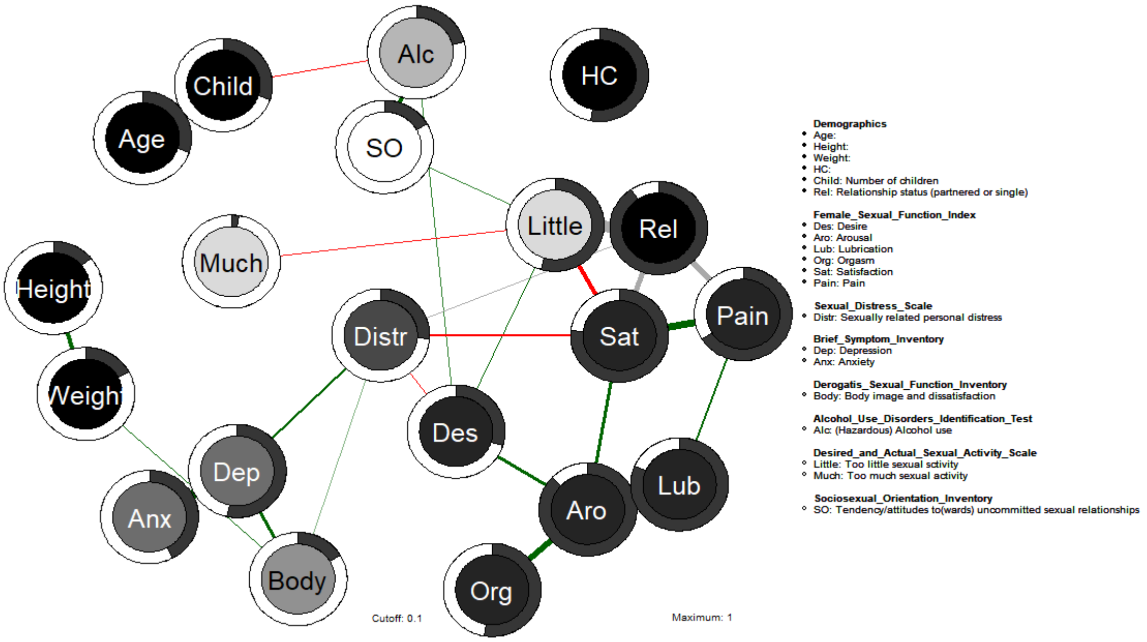


```r
qgraph(fit_Increase$pairwise$wadj,
                    edge.color = fit_Increase$pairwise$edgecolor,
                    layout = 'spring',
                    labels = colNames,
                    maximum = 1,
                    minimum = 0,
                    cut = 0.1,
                    details = TRUE,
                    vsize = 9,
                    label.cex = 1.2,
                    label.scale.equal = TRUE,
                    pie = predR_Increase,
                    pieColor = "gray21",
                    pieBorder = 0.25,
                    groups = groupsNoHc,
                    nodeNames = nodeDescr,
                    legend = TRUE,
                    legend.mode = "style1",
                    legend.cex = 0.3,
                    palette = "gray",
                    negDashed = TRUE,
                    label.color = nodeLabelColors)
```

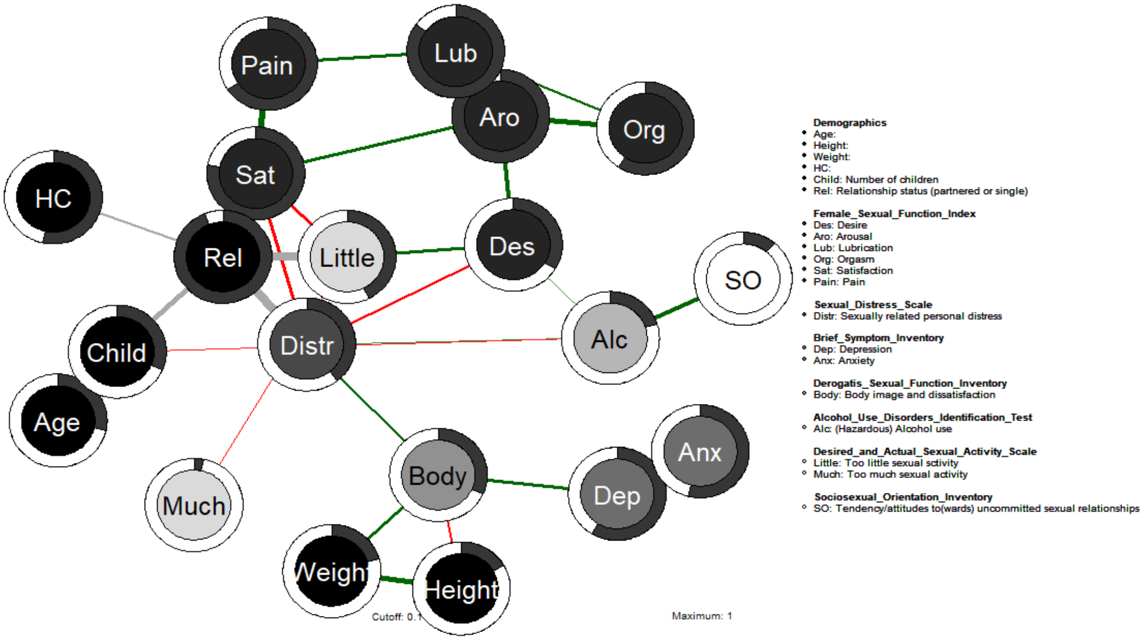

6. Network Estimation excluding Hormonal Contraception

```
#####################
# get rid of HC ####
#####################
# We decided to exclude hormonal contraception from the main analysis since it only related to
# relationship status in the increase group - such an unconnected node can lead to problems
# with the centrality computation

DataNetwork <- DataNetwork[,-4]

colnames(DataNetwork)
##  [1] "age_OLD"           "height_OLD"        "weight_OLD"
##  [4] "biolchild_OLD"     "FSFIdes_OLD"       "FSFIaro_OLD"
##  [7] "FSFIlub_OLD"       "FSFIorg_OLD"       "FSFIsat_OLD"
## [10] "FSFIpain_OLD"      "SDS_OLD"           "BSIdep_OLD"
## [13] "BSIanx_OLD"        "BI_OLD"            "AUDIT_OLD"
## [16] "DSFItoolittle_OLD" "DSFItoomuch_OLD"   "SOI_OLD"
## [19] "haspartner_OLD"    "SDI_groups"        "SDI_change"

##########################
# Subset Data into Groups#
##########################
# double checked that 1 = decrease 2 = stable 3 = increase
Decrease <- subset(DataNetwork, SDI_groups==1, select=age_OLD:haspartner_OLD)
Stable <- subset(DataNetwork, SDI_groups==2, select=age_OLD:haspartner_OLD)
Increase <- subset(DataNetwork, SDI_groups==3, select=age_OLD:haspartner_OLD)

nrow(Decrease) + nrow(Stable) + nrow(Increase) # check whether everyone has been assigned
## [1] 1692

##################################
# Random Subsamples of same size #
##################################

set.seed(17) # to make sure we always get the same sample...
nrow(Decrease)
```

```
## [1] 522
nrow(Stable)
## [1] 687
nrow(Increase) # 483
## [1] 483

randomSample = function(df,n) {
  return (df[sample(nrow(df), n),])
}

DecreaseRS <- randomSample(Decrease, n = 483)
StableRS <- randomSample(Stable, n = 483)
IncreaseRS <- Increase

describe(DecreaseRS)
##                    vars   n   mean    sd median trimmed   mad    min    max
## age_OLD               1 483  25.67  4.84   25.0   25.35  4.45  18.00  43.00
## height_OLD            2 483 166.20  6.44  166.0  166.10  5.93 147.00 196.20
## weight_OLD            3 483  63.58 11.33   62.0   62.29 10.38  41.00 110.00
## biolchild_OLD         4 483   0.50  0.96    0.0    0.29  0.00   0.00   6.00
## FSFIdes_OLD           5 483   3.45  0.87    3.6    3.45  0.89   1.20   5.40
## FSFIaro_OLD           6 483   4.74  1.56    5.4    5.09  0.89   0.00   6.00
## FSFIlub_OLD           7 483   5.16  1.63    6.0    5.62  0.00   0.00   6.00
## FSFIorg_OLD           8 483   4.18  1.84    4.8    4.45  1.78   0.00   6.00
## FSFIsat_OLD           9 483   4.20  1.81    4.8    4.43  1.78   0.28   6.84
## FSFIpain_OLD         10 483   4.32  2.29    5.4    4.65  0.89   0.00   6.05
## SDS_OLD              11 483   7.34  5.30    7.0    6.92  5.93   0.00  26.00
## BSIdep_OLD           12 483   5.35  4.59    4.0    4.69  2.97   0.00  23.00
## BSIanx_OLD           13 483   3.67  3.71    2.0    3.02  2.97   0.00  22.00
## BI_OLD               14 483  26.04  6.48   26.0   25.88  7.41  11.00  44.00
## AUDIT_OLD            15 483   7.74  4.45    7.0    7.15  4.45   1.02  26.00
## DSFItoolittle_OLD    16 483   5.07  4.91    4.0    4.42  4.45   0.00  26.00
## DSFItoomuch_OLD      17 483   0.10  0.47    0.0    0.00  0.00   0.00   4.00
## SOI_OLD              18 483  68.38 42.17   59.0   62.36 26.69   7.00 277.86
## haspartner_OLD       19 483   1.76  0.43    2.0    1.83  0.00   1.00   2.00
##                     range  skew kurtosis   se
## age_OLD             25.00  0.67     0.34 0.22
## height_OLD          49.20  0.23     0.57 0.29
## weight_OLD          69.00  1.25     2.19 0.52
## biolchild_OLD        6.00  2.38     6.81 0.04
## FSFIdes_OLD          4.20  0.00    -0.23 0.04
## FSFIaro_OLD          6.00 -1.94     3.14 0.07
## FSFIlub_OLD          6.00 -2.46     4.93 0.07
## FSFIorg_OLD          6.00 -0.97    -0.16 0.08
## FSFIsat_OLD          6.56 -0.85    -0.61 0.08
## FSFIpain_OLD         6.05 -1.13    -0.40 0.10
## SDS_OLD             26.00  0.66    -0.15 0.24
## BSIdep_OLD          23.00  1.35     1.69 0.21
## BSIanx_OLD          22.00  1.77     3.42 0.17
## BI_OLD              33.00  0.20    -0.41 0.30
## AUDIT_OLD           24.98  1.29     1.72 0.20
## DSFItoolittle_OLD   26.00  1.07     0.73 0.22
## DSFItoomuch_OLD      4.00  5.50    33.08 0.02
## SOI_OLD            270.86  1.69     3.81 1.92
## haspartner_OLD       1.00 -1.23    -0.50 0.02

describe(StableRS)
##                    vars   n   mean    sd median trimmed   mad    min    max
## age_OLD               1 483  25.18  4.73  25.00   24.89  4.45  18.00  43.00
## height_OLD            2 483 165.93  6.13 165.02  165.69  5.96 149.00 195.00
## weight_OLD            3 483  63.21 11.11  61.00   61.92  8.90  44.00 113.00
## biolchild_OLD         4 483   0.52  0.96   0.00    0.31  0.00   0.00   6.00
## FSFIdes_OLD           5 483   3.25  0.92   3.00    3.24  0.89   1.20   5.40
```

```
## FSFIaro_OLD          6 483   4.39  1.74   5.10    4.73  0.89   0.00   6.00
## FSFIlub_OLD          7 483   4.84  1.88   5.70    5.29  0.44   0.00   6.13
## FSFIorg_OLD          8 483   3.85  1.93   4.40    4.06  1.78   0.00   6.00
## FSFIsat_OLD          9 483   4.10  1.84   4.80    4.29  1.78   0.40   6.00
## FSFIpain_OLD        10 483   4.14  2.32   5.40    4.42  0.89   0.00   6.41
## SDS_OLD             11 483   7.44  5.48   7.00    6.95  5.93   0.00  28.00
## BSIdep_OLD          12 483   5.29  4.45   4.00    4.74  4.45   0.00  22.00
## BSIanx_OLD          13 483   3.98  4.15   3.00    3.27  2.97   0.00  23.00
## BI_OLD              14 483  26.50  6.24  26.00   26.36  5.93  11.00  44.00
## AUDIT_OLD           15 483   7.16  4.12   6.00    6.65  3.61   0.93  22.00
## DSFItoolittle_OLD   16 483   4.60  5.03   3.00    3.76  4.45   0.00  22.00
## DSFItoomuch_OLD     17 483   0.09  0.46   0.00    0.00  0.00   0.00   4.00
## SOI_OLD             18 483  60.81 37.68  56.00   55.68 23.72   7.00 335.68
## haspartner_OLD      19 483   1.76  0.43   2.00    1.82  0.00   1.00   2.00
##                    range  skew kurtosis   se
## age_OLD            25.00  0.63     0.41 0.22
## height_OLD         46.00  0.42     0.65 0.28
## weight_OLD         69.00  1.29     2.34 0.51
## biolchild_OLD       6.00  2.17     5.33 0.04
## FSFIdes_OLD         4.20  0.10    -0.31 0.04
## FSFIaro_OLD         6.00 -1.51     1.18 0.08
## FSFIlub_OLD         6.13 -1.85     1.99 0.09
## FSFIorg_OLD         6.00 -0.73    -0.63 0.09
## FSFIsat_OLD         5.60 -0.76    -0.78 0.08
## FSFIpain_OLD        6.41 -0.98    -0.69 0.11
## SDS_OLD            28.00  0.83     0.49 0.25
## BSIdep_OLD         22.00  1.14     1.06 0.20
## BSIanx_OLD         23.00  1.90     4.25 0.19
## BI_OLD             33.00  0.22    -0.45 0.28
## AUDIT_OLD          21.07  1.02     0.61 0.19
## DSFItoolittle_OLD  22.00  1.25     0.75 0.23
## DSFItoomuch_OLD     4.00  6.04    40.15 0.02
## SOI_OLD           328.68  2.36     9.52 1.71
## haspartner_OLD      1.00 -1.20    -0.56 0.02

describe(IncreaseRS)
##                   vars   n   mean    sd median trimmed   mad    min    max
## age_OLD              1 483  25.54  4.94  25.00   25.25  5.93  18.00  44.00
## height_OLD           2 483 166.18  6.18 166.00  166.13  5.93 146.00 186.00
## weight_OLD           3 483  63.81 11.77  62.00   62.41  8.90  42.00 115.00
## biolchild_OLD        4 483   0.57  1.05   0.00    0.33  0.00   0.00   9.00
## FSFIdes_OLD          5 483   3.10  0.92   3.00    3.08  0.89   1.20   5.40
## FSFIaro_OLD          6 483   4.33  1.82   5.10    4.66  0.89   0.00   6.00
## FSFIlub_OLD          7 483   4.79  1.98   5.70    5.23  0.44   0.00   7.44
## FSFIorg_OLD          8 483   3.87  2.04   4.80    4.09  1.78   0.00   6.31
## FSFIsat_OLD          9 483   4.18  1.85   4.80    4.38  1.78   0.40   6.00
## FSFIpain_OLD        10 483   4.27  2.31   5.40    4.58  0.89   0.00   6.21
## SDS_OLD             11 483   7.60  5.79   7.00    7.10  5.93   0.00  27.00
## BSIdep_OLD          12 483   5.65  4.83   4.00    5.03  4.45   0.00  24.00
## BSIanx_OLD          13 483   4.08  4.24   3.00    3.34  2.97   0.00  23.00
## BI_OLD              14 483  26.67  6.85  27.00   26.62  7.41  11.00  45.06
## AUDIT_OLD           15 483   6.82  4.24   6.00    6.20  2.97   0.74  31.00
## DSFItoolittle_OLD   16 483   4.12  4.47   3.00    3.41  4.45   0.00  29.00
## DSFItoomuch_OLD     17 483   0.16  0.72   0.00    0.00  0.00   0.00   7.63
## SOI_OLD             18 483  60.69 39.16  55.75   55.08 27.80   6.00 249.00
## haspartner_OLD      19 483   1.81  0.40   2.00    1.88  0.00   1.00   2.00
##                    range  skew kurtosis   se
## age_OLD            26.00  0.65     0.38 0.22
## height_OLD         40.00  0.08     0.12 0.28
## weight_OLD         73.00  1.32     2.38 0.54
## biolchild_OLD       9.00  2.54     9.95 0.05
## FSFIdes_OLD         4.20  0.16    -0.20 0.04
## FSFIaro_OLD         6.00 -1.37     0.72 0.08
```

```
## FSFIlub_OLD         7.44 -1.72     1.43 0.09
## FSFIorg_OLD         6.31 -0.70    -0.88 0.09
## FSFIsat_OLD         5.60 -0.80    -0.81 0.08
## FSFIpain_OLD        6.21 -1.06    -0.53 0.11
## SDS_OLD            27.00  0.72    -0.03 0.26
## BSIdep_OLD         24.00  1.12     0.85 0.22
## BSIanx_OLD         23.00  1.80     3.60 0.19
## BI_OLD             34.06  0.07    -0.53 0.31
## AUDIT_OLD          30.26  1.58     3.27 0.19
## DSFItoolittle_OLD  29.00  1.52     2.96 0.20
## DSFItoomuch_OLD     7.63  6.15    44.68 0.03
## SOI_OLD           243.00  1.70     3.92 1.78
## haspartner_OLD      1.00 -1.54     0.37 0.02

table(DecreaseRS[,19]) # same random sample!
## 
##   1   2 
## 115 368
table(DecreaseRS[,19])[1]/483
##         1 
## 0.2380952
table(DecreaseRS[,19])[2]/483
##         2 
## 0.7619048

table(StableRS[,19])
## 
##   1   2 
## 117 366
table(StableRS[,19])[1]/483
##        1 
## 0.242236
table(StableRS[,19])[2]/483
##        2 
## 0.757764

table(IncreaseRS[,19])
## 
##   1   2 
##  94 389
table(IncreaseRS[,19])[1]/483
##        1 
## 0.194617
table(IncreaseRS[,19])[2]/483
##        2 
## 0.805383

######################
# Network estimation #
######################

colnames(DataNetwork)
##  [1] "age_OLD"           "height_OLD"        "weight_OLD"       
##  [4] "biolchild_OLD"     "FSFIdes_OLD"       "FSFIaro_OLD"      
##  [7] "FSFIlub_OLD"       "FSFIorg_OLD"       "FSFIsat_OLD"      
## [10] "FSFIpain_OLD"      "SDS_OLD"           "BSIdep_OLD"       
## [13] "BSIanx_OLD"        "BI_OLD"            "AUDIT_OLD"        
## [16] "DSFItoolittle_OLD" "DSFItoomuch_OLD"   "SOI_OLD"          
## [19] "haspartner_OLD"    "SDI_groups"        "SDI_change"

VarDomain <- c("g","g","g",
               "p","g","g",
               "g","g","g",
```

```
               "g","g","g",
               "g","g","g",
               "g","g","g",
               "c")

VarLevel <- c(1,1,1,
              1,1,1,
              1,1,1,
              1,1,1,
              1,1,1,
              1,1,1,
              2)

library(mgm)

fit_Decrease <- mgm(data = as.matrix(DecreaseRS),
                    type = VarDomain,
                    level = VarLevel,
                    k = 2,
                    lambdaSel = 'EBIC',
                    lambdaGam = 0.5) # hyper parameter

fit_Stable <- mgm(data = as.matrix(StableRS),
                  type = VarDomain,
                  level = VarLevel,
                  k = 2,
                  lambdaSel = 'EBIC',
                  lambdaGam = 0.5) # hyper parameter

fit_Increase <- mgm(data = as.matrix(IncreaseRS),
                    type = VarDomain,
                    level = VarLevel,
                    k = 2,
                    lambdaSel = 'EBIC',
                    lambdaGam = 0.5) # hyper parameter

########################
# Predictive Strength ##
########################

pred_Decrease <- predict(fit_Decrease,
                         DecreaseRS,
                         errorCon = 'R2') #save R2 into an object (varExpl = cont var)

pred_Stable <- predict(fit_Stable,
                       StableRS,
                       errorCon = 'R2') #save R2 into an object (varExpl = cont var)

pred_Increase <- predict(fit_Increase,
                         IncreaseRS,
                         errorCon = 'R2') #save R2 into an object (varExpl = cont var)

predR_Decrease <- pred_Decrease$errors$Error.R2
predR_Decrease <- as.numeric(as.character(predR_Decrease))

predR_Stable <- pred_Stable$errors$Error.R2
predR_Stable <- as.numeric(as.character(predR_Stable))

predR_Increase <- pred_Increase$errors$Error.R2
predR_Increase <- as.numeric(as.character(predR_Increase))

# set 'predictability' of categorical variable
predR_Decrease[19] <- pred_Decrease$errors$Error.CC[19]
```

```
predR_Stable[19] <- pred_Stable$errors$Error.CC[19]
predR_Increase[19] <- pred_Increase$errors$Error.CC[19]

mean(predR_Decrease, na.rm = TRUE) #mean variance explained across all nodes
## [1] 0.4055263
sd(predR_Decrease, na.rm = TRUE)
## [1] 0.2534266

pred_Decrease$errors
##             Variable Error.R2 Error.CC Error.nCC CCmarg
## 1            age_OLD    0.255       NA        NA     NA
## 2         height_OLD    0.100       NA        NA     NA
## 3         weight_OLD    0.241       NA        NA     NA
## 4      biolchild_OLD    0.256       NA        NA     NA
## 5        FSFIdes_OLD    0.208       NA        NA     NA
## 6        FSFIaro_OLD    0.828       NA        NA     NA
## 7        FSFIlub_OLD    0.774       NA        NA     NA
## 8        FSFIorg_OLD    0.505       NA        NA     NA
## 9        FSFIsat_OLD    0.666       NA        NA     NA
## 10      FSFIpain_OLD    0.599       NA        NA     NA
## 11           SDS_OLD    0.286       NA        NA     NA
## 12        BSIdep_OLD    0.541       NA        NA     NA
## 13        BSIanx_OLD    0.449       NA        NA     NA
## 14            BI_OLD    0.326       NA        NA     NA
## 15         AUDIT_OLD    0.198       NA        NA     NA
## 16 DSFItoolittle_OLD    0.452       NA        NA     NA
## 17   DSFItoomuch_OLD    0.040       NA        NA     NA
## 18           SOI_OLD    0.111       NA        NA     NA
## 19     haspartner_OLD       NA     0.87     0.452  0.763

mean(predR_Stable, na.rm = TRUE) #mean variance explained across all nodes
## [1] 0.4295789
sd(predR_Stable, na.rm = TRUE)
## [1] 0.2742941

pred_Stable$errors
##             Variable Error.R2 Error.CC Error.nCC CCmarg
## 1            age_OLD    0.300       NA        NA     NA
## 2         height_OLD    0.138       NA        NA     NA
## 3         weight_OLD    0.171       NA        NA     NA
## 4      biolchild_OLD    0.299       NA        NA     NA
## 5        FSFIdes_OLD    0.306       NA        NA     NA
## 6        FSFIaro_OLD    0.870       NA        NA     NA
## 7        FSFIlub_OLD    0.814       NA        NA     NA
## 8        FSFIorg_OLD    0.526       NA        NA     NA
## 9        FSFIsat_OLD    0.768       NA        NA     NA
## 10      FSFIpain_OLD    0.680       NA        NA     NA
## 11           SDS_OLD    0.271       NA        NA     NA
## 12        BSIdep_OLD    0.535       NA        NA     NA
## 13        BSIanx_OLD    0.420       NA        NA     NA
## 14            BI_OLD    0.163       NA        NA     NA
## 15         AUDIT_OLD    0.239       NA        NA     NA
## 16 DSFItoolittle_OLD    0.549       NA        NA     NA
## 17   DSFItoomuch_OLD    0.029       NA        NA     NA
## 18           SOI_OLD    0.167       NA        NA     NA
## 19     haspartner_OLD       NA    0.917     0.658  0.757

mean(predR_Increase, na.rm = TRUE) #mean variance explained across all nodes
## [1] 0.4515263
sd(predR_Increase, na.rm = TRUE)
## [1] 0.2759063

pred_Increase$errors
```

```
##              Variable Error.R2 Error.CC Error.nCC CCmarg
## 1             age_OLD    0.283       NA        NA     NA
## 2          height_OLD    0.166       NA        NA     NA
## 3          weight_OLD    0.208       NA        NA     NA
## 4       biolchild_OLD    0.286       NA        NA     NA
## 5         FSFIdes_OLD    0.336       NA        NA     NA
## 6         FSFIaro_OLD    0.880       NA        NA     NA
## 7         FSFIlub_OLD    0.850       NA        NA     NA
## 8         FSFIorg_OLD    0.595       NA        NA     NA
## 9         FSFIsat_OLD    0.779       NA        NA     NA
## 10       FSFIpain_OLD    0.657       NA        NA     NA
## 11            SDS_OLD    0.393       NA        NA     NA
## 12         BSIdep_OLD    0.587       NA        NA     NA
## 13         BSIanx_OLD    0.541       NA        NA     NA
## 14             BI_OLD    0.308       NA        NA     NA
## 15          AUDIT_OLD    0.201       NA        NA     NA
## 16 DSFItoolittle_OLD    0.426       NA        NA     NA
## 17   DSFItoomuch_OLD    0.031       NA        NA     NA
## 18            SOI_OLD    0.110       NA        NA     NA
## 19      haspartner_OLD       NA    0.942     0.702  0.805
```

7. Network Visualization excluding Hormonal Contraception

```
############################
# Network Layout Specifics #
############################

colNames <- c("Age", "Height", "Weight", "Child", "Des", "Aro",
              "Lub", "Org", "Sat", "Pain", "Distr", "Dep", "Anx",
              "Body", "Alc", "Little", "Much", "SO", "Rel")

nodeDescr <- c("", "", "", "Number of children", "Desire",
               "Arousal", "Lubrication", "Orgasm", "Satisfaction", "Pain",
               "Sexually related personal distress", "Depression", "Anxiety",
               "Body image and dissatisfaction", "(Hazardous) Alcohol use",
               "Too little sexual activity", "Too much sexual activity",
               "Tendency/attitudes to(wards) uncommitted sexual relationships",
               "Relationship status (partnered or single)")

groupsNoHc <- list(Demographics = c(1:4,19),
                   Female_Sexual_Function_Index = c(5:10),
                   Sexual_Distress_Scale = c(11),
                   Brief_Symptom_Inventory = c(12:13),
                   Derogatis_Sexual_Function_Inventory = c(14),
                   Alcohol_Use_Disorders_Identification_Test = c(15),
                   Desired_and_Actual_Sexual_Activity_Scale = c(16:17),
                   Sociosexual_Orientation_Inventory = c(18))

nodeLabelColors <- c("white", "white", "white", "white",
                     "white","white","white","white","white","white",
                     "white",
                     "white","white",
                     "black","black","black","black","black",
                     "white")

#Plot Networks
# We adjusted the visualization so that the edges are also in gray-scale and so that negative
# edges are visualized as dashed edges

library(qgraph)
```

```
fit_Decrease$pairwise$edgecolor[which(fit_Decrease$pairwise$edgecolor=="red")]<- "gray50"
fit_Decrease$pairwise$edgecolor[which(fit_Decrease$pairwise$edgecolor=="darkgreen")]<- "gray70"
fit_Decrease$pairwise$edgecolor[which(fit_Decrease$pairwise$edgecolor=="darkgrey")]<- "black"
fit_Decrease$pairwise$edgecolor[c(16),19] <- "gray50" # categ relationship
fit_Decrease$pairwise$edgecolor[c(19),16] <- "gray50"
fit_Decrease$pairwise$edgecolor[c(9,10),19] <- "gray70"
fit_Decrease$pairwise$edgecolor[19,c(9,10)] <- "gray70"
fit_Decrease$pairwise$wadj[which(fit_Decrease$pairwise$edgecolor=="gray50")] <- fit_Decrease$pa
irwise$wadj[which(fit_Decrease$pairwise$edgecolor=="gray50")]*-1

fit_Decrease$pairwise$wadj[which(fit_Decrease$pairwise$edgecolor=="gray50")]
##  [1] -0.1200629 -0.1844332 -0.1624988 -0.1844332 -0.1200629 -0.1624988
##  [7] -0.1121987 -0.7201683 -0.1121987 -0.7201683

fit_Decrease$pairwise$wadj[c(16),19]
## [1] -0.7201683

fit_Decrease$pairwise$wadj[19, 16]
## [1] -0.7201683

qDecrease <- qgraph(fit_Decrease$pairwise$wadj,
                    edge.color = fit_Decrease$pairwise$edgecolor,
                    layout = 'spring',
                    labels = colNames,
                    maximum = 1,
                    minimum = 0,
                    cut = 0.1,
                    details = TRUE,
                    vsize = 9,
                    label.cex = 1.2,
                    label.scale.equal = TRUE,
                    pie = predR_Decrease,
                    pieColor = "gray21",
                    pieBorder = 0.25,
                    groups = groupsNoHc,
                    nodeNames = nodeDescr,
                    legend = TRUE,
                    legend.mode = "style1",
                    legend.cex = 0.5,
                    palette = "gray",
                    negDashed = TRUE,
                    label.color = nodeLabelColors)
```

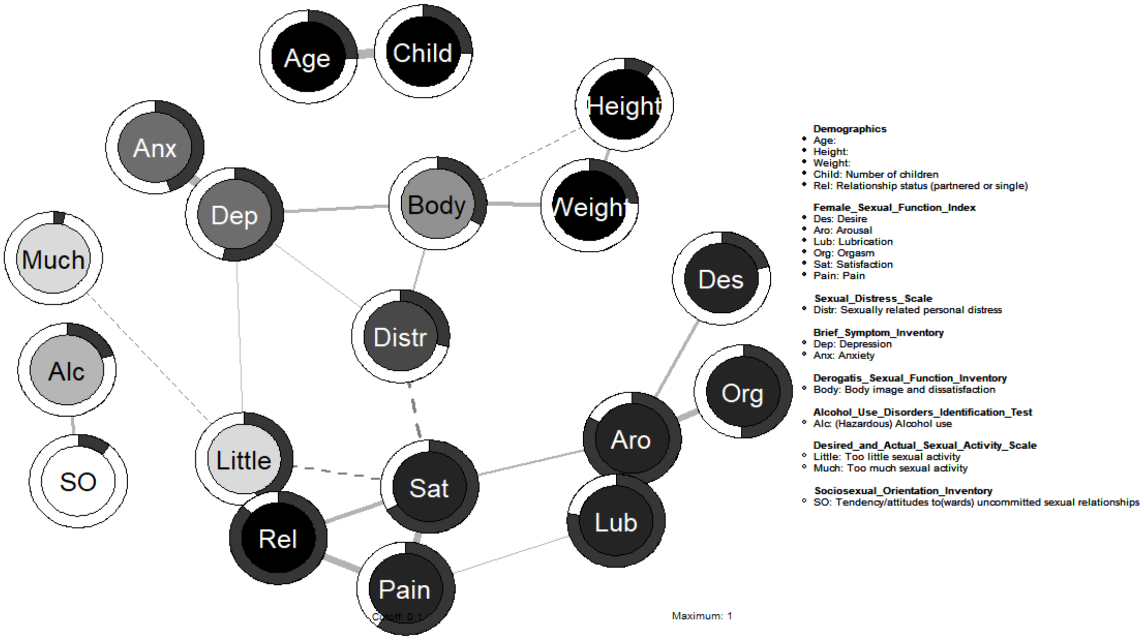


```r
fit_Stable$pairwise$edgecolor[which(fit_Stable$pairwise$edgecolor=="red")]<- "gray50"
fit_Stable$pairwise$edgecolor[which(fit_Stable$pairwise$edgecolor=="darkgreen")]<- "gray70"
fit_Stable$pairwise$edgecolor[which(fit_Stable$pairwise$edgecolor=="darkgrey")]<- "black"
fit_Stable$pairwise$edgecolor[c(16),19] <- "gray50" # categ relationship
fit_Stable$pairwise$edgecolor[c(19),16] <- "gray50"
fit_Stable$pairwise$edgecolor[c(9,10,4,11),19] <- "gray70"
fit_Stable$pairwise$edgecolor[19,c(9,10,4,11)] <- "gray70"
fit_Stable$pairwise$wadj[which(fit_Stable$pairwise$edgecolor=="gray50")] <- fit_Stable$pairwise
$wadj[which(fit_Stable$pairwise$edgecolor=="gray50")]*-1

fit_Stable$pairwise$wadj[which(fit_Stable$pairwise$edgecolor=="gray50")]
##  [1] -0.1315370 -0.1247692 -0.1668837 -0.2775090 -0.1247692 -0.1668837
##  [7] -0.1315370 -0.2775090 -0.1068109 -0.6522733 -0.1068109 -0.6522733

fit_Stable$pairwise$wadj[c(9,10,4,11),19]
## [1] 0.4016786 0.4042179 0.2631987 0.2038407

fit_Stable$pairwise$wadj[c(16),19]
## [1] -0.6522733

qStable <- qgraph(fit_Stable$pairwise$wadj,
                  edge.color = fit_Stable$pairwise$edgecolor,
                  layout = 'spring',
                  labels = colNames,
                  maximum = 1,
                  minimum = 0,
                  cut = 0.1,
                  details = TRUE,
                  vsize = 9,
                  label.cex = 1.2,
                  label.scale.equal = TRUE,
                  pie = predR_Stable,
                  pieColor = "gray21",
                  pieBorder = 0.25,
                  groups = groupsNoHc,
                  nodeNames = nodeDescr,
                  legend = TRUE,
                  legend.mode = "style1",
```

```
                  legend.cex = 0.5,
                  palette = "gray",
                  negDashed = TRUE,
                  label.color = nodeLabelColors)
```

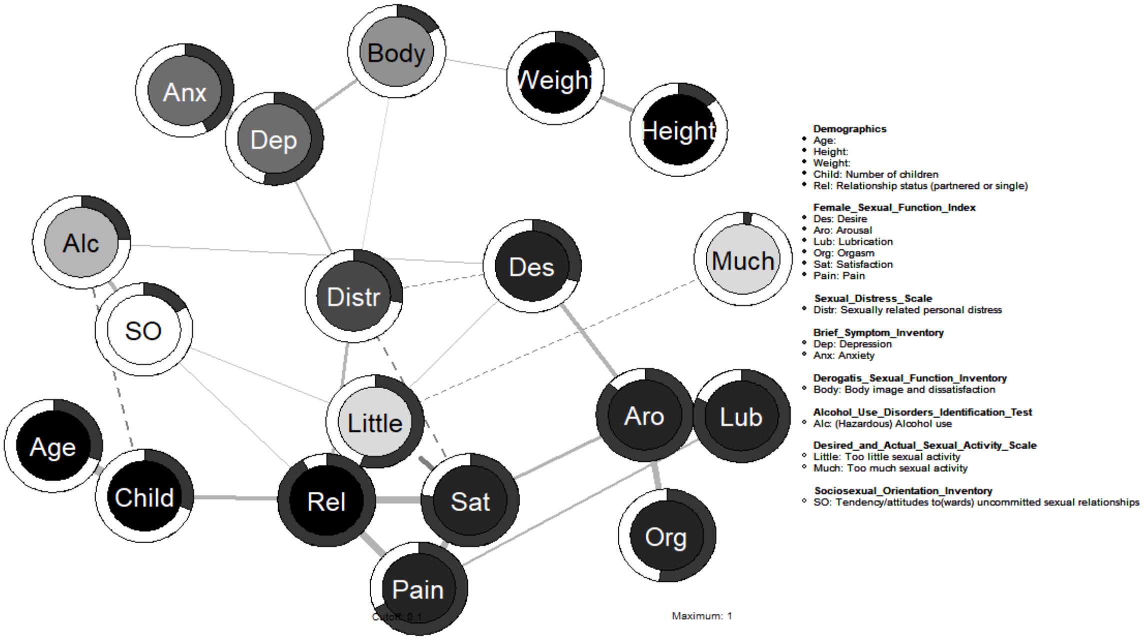


```
fit_Increase$pairwise$edgecolor[which(fit_Increase$pairwise$edgecolor=="red")]<- "gray50"
fit_Increase$pairwise$edgecolor[which(fit_Increase$pairwise$edgecolor=="darkgreen")]<- "gray70"
fit_Increase$pairwise$edgecolor[which(fit_Increase$pairwise$edgecolor=="darkgrey")]<- "black"
fit_Increase$pairwise$edgecolor[c(16),19] <- "gray50" # categ relationship
fit_Increase$pairwise$edgecolor[c(19),16] <- "gray50"
fit_Increase$pairwise$edgecolor[c(9,4,11),19] <- "gray70"
fit_Increase$pairwise$edgecolor[19,c(9,4,11)] <- "gray70"
fit_Increase$pairwise$wadj[which(fit_Increase$pairwise$edgecolor=="gray50")] <- fit_Increase$pa
irwise$wadj[which(fit_Increase$pairwise$edgecolor=="gray50")]*-1

fit_Increase$pairwise$wadj[which(fit_Increase$pairwise$edgecolor=="gray50")]
##  [1] -0.1529757 -0.1063900 -0.1759594 -0.2062433 -0.2153194 -0.1759594
##  [7] -0.2062433 -0.1529757 -0.1063900 -0.2153194 -0.1191332 -0.4778137
## [13] -0.1191332 -0.4778137

fit_Increase$pairwise$wadj[c(19),16]
## [1] -0.4778137

fit_Increase$pairwise$wadj[16, 19]
## [1] -0.4778137

qIncrease <- qgraph(fit_Increase$pairwise$wadj,
                    edge.color = fit_Increase$pairwise$edgecolor,
                    layout = 'spring',
                    labels = colNames,
                    maximum = 1,
                    minimum = 0,
                    cut = 0.1,
                    details = TRUE,
                    vsize = 9,
                    label.cex = 1.2,
                    label.scale.equal = TRUE,
                    pie = predR_Increase,
                    pieColor = "gray21",
```

```r
                    pieBorder = 0.25,
                    groups = groupsNoHc,
                    nodeNames = nodeDescr,
                    legend = TRUE,
                    legend.mode = "style1",
                    legend.cex = 0.5,
                    palette = "gray",
                    negDashed = TRUE,
                    label.color = nodeLabelColors)
```

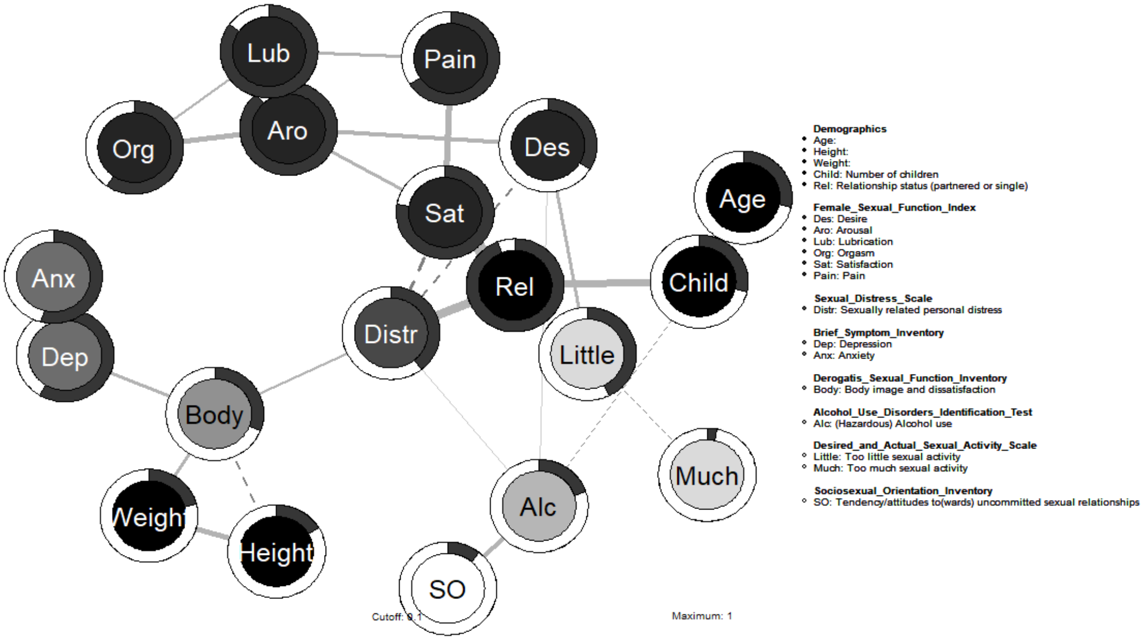


```r
length(qDecrease$Edgelist$weight)
## [1] 22
length(qStable$Edgelist$weight)
## [1] 28
length(qIncrease$Edgelist$weight)
## [1] 27

LayoutAverage <- averageLayout(qDecrease, qStable, qIncrease)
LayoutAverage
##                [,1]          [,2]
##  [1,] -0.11530750 -1.0000000000
##  [2,] -1.00000000  0.0322473495
##  [3,] -0.85772120  0.3954204609
##  [4,]  0.10621364 -0.7828882333
##  [5,]  0.06047028  0.4589610011
##  [6,]  0.47985819  0.7147809183
##  [7,]  0.75187419  0.6374354693
##  [8,]  0.18336058  1.0000000000
##  [9,]  0.67935638  0.0586519881
## [10,]  1.00000000 -0.0006634476
## [11,]  0.05954809 -0.3086100067
## [12,] -0.54064749 -0.5866338083
## [13,] -0.81726660 -0.5840684455
## [14,] -0.54255762 -0.0567517604
## [15,] -0.42461600  0.5310292915
## [16,]  0.37896893 -0.1213227140
## [17,]  0.65704109 -0.8224269149
## [18,] -0.41163631  0.9333091298
## [19,]  0.59209635 -0.3196238530
```

```
LayoutAverage[16,1] <- LayoutAverage[16,1]-0.15 # Move the “little” node to make edges visible
LayoutAverage[16,2] <- -0.0006634476

LayoutAverage
##              [,1]          [,2]
##  [1,] -0.11530750 -1.0000000000
##  [2,] -1.00000000  0.0322473495
##  [3,] -0.85772120  0.3954204609
##  [4,]  0.10621364 -0.7828882333
##  [5,]  0.06047028  0.4589610011
##  [6,]  0.47985819  0.7147809183
##  [7,]  0.75187419  0.6374354693
##  [8,]  0.18336058  1.0000000000
##  [9,]  0.67935638  0.0586519881
## [10,]  1.00000000 -0.0006634476
## [11,]  0.05954809 -0.3086100067
## [12,] -0.54064749 -0.5866338083
## [13,] -0.81726660 -0.5840684455
## [14,] -0.54255762 -0.0567517604
## [15,] -0.42461600  0.5310292915
## [16,]  0.22896893 -0.0006634476
## [17,]  0.65704109 -0.8224269149
## [18,] -0.41163631  0.9333091298
## [19,]  0.59209635 -0.3196238530

pdf('FSFINetworkWithPrednoHCGrayScale.pdf', width = 55, height = 15)
par(mfrow = c(1,3))
qDecrease2 <- qgraph(fit_Decrease$pairwise$wadj,
                    edge.color = fit_Decrease$pairwise$edgecolor,
                    layout = LayoutAverage,
                    labels = colNames,
                    maximum = 1,
                    minimum = 0,
                    cut = 0.1,
                    details = TRUE,
                    vsize = 10,
                    label.cex = 1.2,
                    label.scale.equal = TRUE,
                    pie = predR_Decrease,
                    pieColor = "gray21",
                    pieBorder = 0.25,
                    groups = groupsNoHc,
                    nodeNames = nodeDescr,
                    legend = TRUE,
                    legend.mode = "style1",
                    legend.cex = 0.5,
                    palette = "gray",
                    negDashed = TRUE,
                    label.color = nodeLabelColors,
                    repulsion = 2)

qStable2 <- qgraph(fit_Stable$pairwise$wadj,
                  edge.color = fit_Stable$pairwise$edgecolor,
                  layout = LayoutAverage,
                  labels = colNames,
                  maximum = 1,
                  minimum = 0,
                  cut = 0.1,
                  details = TRUE,
                  vsize = 10,
                  label.cex = 1.2,
                  label.scale.equal = TRUE,
```

```
                  pie = predR_Stable,
                  pieColor = "gray21",
                  pieBorder = 0.25,
                  groups = groupsNoHc,
                  nodeNames = nodeDescr,
                  legend = TRUE,
                  legend.mode = "style1",
                  legend.cex = 0.5,
                  palette = "gray",
                  negDashed = TRUE,
                  label.color = nodeLabelColors,
                  repulsion = 2)

qIncrease2 <- qgraph(fit_Increase$pairwise$wadj,
                    edge.color = fit_Increase$pairwise$edgecolor,
                    layout = LayoutAverage,
                    labels = colNames,
                    maximum = 1,
                    minimum = 0,
                    cut = 0.1,
                    details = TRUE,
                    vsize = 10,
                    label.cex = 1.2,
                    label.scale.equal = TRUE,
                    pie = predR_Increase,
                    pieColor = "gray21",
                    pieBorder = 0.25,
                    groups = groupsNoHc,
                    nodeNames = nodeDescr,
                    legend = TRUE,
                    legend.mode = "style1",
                    legend.cex = 0.5,
                    palette = "gray",
                    negDashed = TRUE,
                    label.color = nodeLabelColors,
                    repulsion = 2)
dev.off()
## png
##   2
```

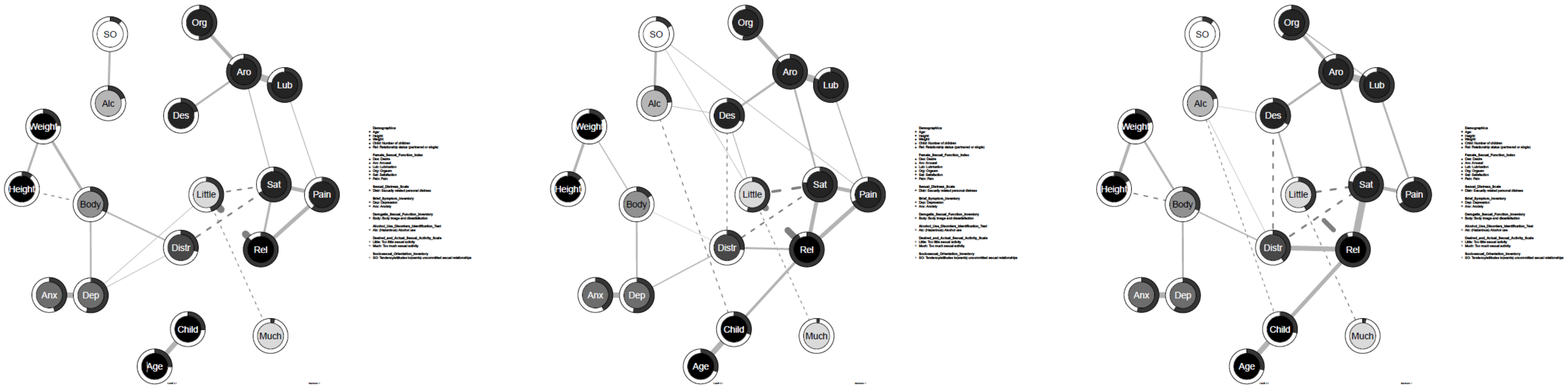
SO
Org
Aro
Lub
Alc
Des
Weight
Height
Body
Little
Sat
Pain
Distr
Rel
Anx
Dep
Child
Much
Age
Demographics
Age:
Height:
Weight:
Child: Number of children
Rel: Relationship status (partnered or single)
Female_Sexual_Function_Index
Des: Desire
Aro: Arousal
Lub: Lubrication
Org: Orgasm
Sat: Satisfaction
Pain: Pain
Sexual_Distress_Scale
Distr: Sexually related personal distress
Brief_Symptom_Inventory
Dep: Depression
Anx: Anxiety
Derogatis_Sexual_Function_Inventory
Body: Body image and dissatisfaction
Alcohol_Use_Disorders_Identification_Test
Alc: (Hazardous) Alcohol use
Desired_and_Actual_Sexual_Activity_Scale
Little: Too little sexual activity
Much: Too much sexual activity
Sociosexual_Orientation_Inventory
SO: Tendency/attitudes to(wards) uncommitted sexual relationships

```
par(mfrow = c(1,1))

# CUSTOM MADE CENTRALITY PLOT
cenDecrease <- centralityTable(qDecrease2, standardized = FALSE, relative = TRUE)
cenIncrease <- centralityTable(qIncrease2, standardized = FALSE, relative = TRUE)
cenStable <- centralityTable(qStable2, standardized = FALSE, relative = TRUE)

pdf("CustomCentralityFSFINetwork.pdf", width = 15, height = 7)
plot(cenDecrease$value[39:57],
     type = "b",
     bty = "l",
     family = "sans",
     las = 1,
     lwd = 1,
     xaxt="n",
     xlab = "Node",
     ylab = "Strength Centrality")
axis(side = 1, labels = colNames, at = c(1:19))
lines(cenIncrease$value[39:57], type = "b", lty = "dotted")
lines(cenStable$value[39:57], type = "b", lty = "dashed")
legend(x = 1, legend=c("Decrease", "Stable", "Increase"),
       lty=c("solid", "dashed", "dotted"))
dev.off()
## png
##   2
```

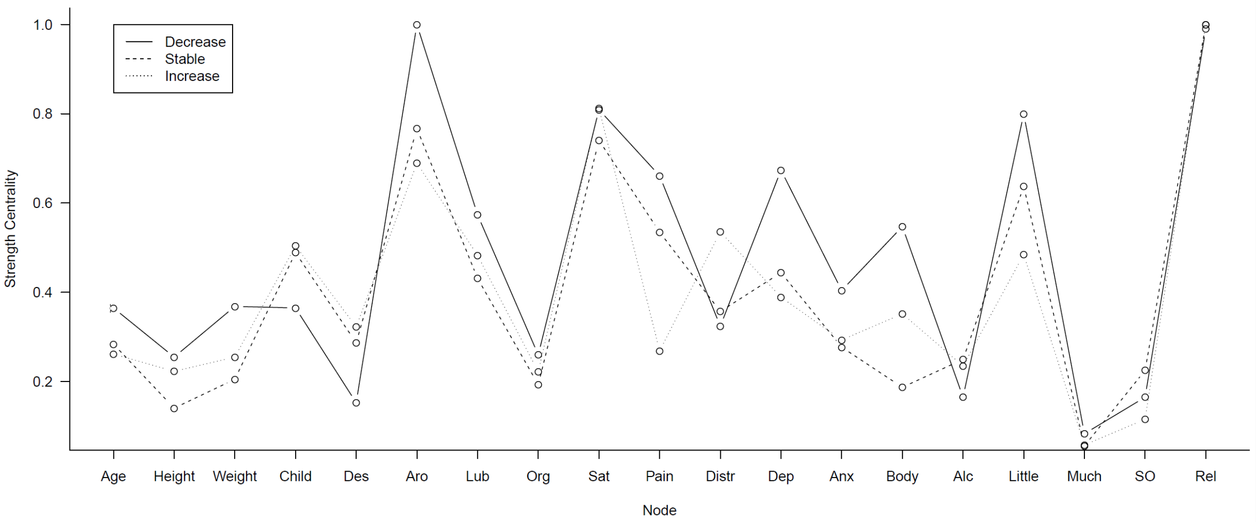


```
##############################################################
# RETRIEVE EDGE WEIGHTS CATERGORICAL (Relationship Status)
# Decrease Network
fit_Decrease$rawfactor$indicator[[1]][13,] # 9 and 19 satisfaction
## [1]  9 19
fit_Decrease$rawfactor$indicator[[1]][14,] # 10 and 19 pain
## [1] 10 19
fit_Decrease$rawfactor$indicator[[1]][22,] # 16 and 19 too little
## [1] 16 19
fit_Decrease$rawfactor$weights[[1]][13]
## [[1]]
## [[1]][[1]]
##              [,1]
## V19.2 0.2562422
##
```

```
## [[1]][[2]]
## [1] -0.1982032  0.1982032
fit_Decrease$rawfactor$weights[[1]][14]
## [[1]]
## [[1]][[1]]
##             [,1]
## V19.2 0.4611961
##
## [[1]][[2]]
## [1] -0.3254598  0.3254598
fit_Decrease$rawfactor$weights[[1]][22]
## [[1]]
## [[1]][[1]]
##              [,1]
## V19.2 -0.9094696
##
## [[1]][[2]]
## [1]  0.530867 -0.530867

# Stable Network
fit_Stable$rawfactor$indicator[[1]][5,] # 4 child
## [1]  4 19
fit_Stable$rawfactor$indicator[[1]][17,] # 9 satisfaction
## [1]  9 19
fit_Stable$rawfactor$indicator[[1]][19,] # 10 pain
## [1] 10 19
fit_Stable$rawfactor$indicator[[1]][22,] # 11 distress
## [1] 11 19
fit_Stable$rawfactor$indicator[[1]][28,] # 16 too little
## [1] 16 19
fit_Stable$rawfactor$weights[[1]][5]
## [[1]]
## [[1]][[1]]
##             [,1]
## V19.2 0.4400181
##
## [[1]][[2]]
## [1] -0.08637923  0.08637923
fit_Stable$rawfactor$weights[[1]][17]
## [[1]]
## [[1]][[1]]
##             [,1]
## V19.2 0.2881257
##
## [[1]][[2]]
## [1] -0.5152314  0.5152314
fit_Stable$rawfactor$weights[[1]][19]
## [[1]]
## [[1]][[1]]
##             [,1]
## V19.2 0.4640637
##
## [[1]][[2]]
## [1] -0.3443721  0.3443721
fit_Stable$rawfactor$weights[[1]][22]
## [[1]]
## [[1]][[1]]
##             [,1]
## V19.2 0.1816205
##
## [[1]][[2]]
## [1] -0.226061  0.226061
fit_Stable$rawfactor$weights[[1]][28]
```

```
## [[1]]
## [[1]][[1]]
##             [,1]
## V19.2 -0.8035653
##
## [[1]][[2]]
## [1]  0.5009813 -0.5009813

# Increase Network
fit_Increase$rawfactor$indicator[[1]][6,] # 4 child
## [1]  4 19
fit_Increase$rawfactor$indicator[[1]][19,] # 10 pain
## [1]  9 19
fit_Increase$rawfactor$indicator[[1]][22,] # 11 distress
## [1] 11 19
fit_Increase$rawfactor$indicator[[1]][27,] # 16 too little
## [1] 16 19
fit_Increase$rawfactor$weights[[1]][6]
## [[1]]
## [[1]][[1]]
##            [,1]
## V19.2 0.4287313
##
## [[1]][[2]]
## [1] -0.3607776  0.3607776
fit_Increase$rawfactor$weights[[1]][19]
## [[1]]
## [[1]][[1]]
##            [,1]
## V19.2 0.5852141
##
## [[1]][[2]]
## [1] -0.8122344  0.8122344
fit_Increase$rawfactor$weights[[1]][22]
## [[1]]
## [[1]][[1]]
##            [,1]
## V19.2 0.6671867
##
## [[1]][[2]]
## [1] -0.3205457  0.3205457
fit_Increase$rawfactor$weights[[1]][27]
## [[1]]
## [[1]][[1]]
##             [,1]
## V19.2 -0.6792773
##
## [[1]][[2]]
## [1]  0.2763501 -0.2763501
```

8. Network Stability

```
##############################################################
# BOOTNET
library(bootnet)
fit_DecreaseBoot <- estimateNetwork(as.matrix(DecreaseRS),
                                    default = "mgm",
                                    type = VarDomain,
                                    lev = VarLevel,
                                    degree = 2,
                                    criterion = "EBIC",
                                    tuning = 0.5)
```

```
fit_StableBoot <- estimateNetwork(as.matrix(StableRS),
                                  default = "mgm",
                                  type = VarDomain,
                                  lev = VarLevel,
                                  degree = 2,
                                  criterion = "EBIC",
                                  tuning = 0.5)

fit_IncreaseBoot <- estimateNetwork(as.matrix(IncreaseRS),
                                    default = "mgm",
                                    type = VarDomain,
                                    lev = VarLevel,
                                    degree = 2,
                                    criterion = "EBIC",
                                    tuning = 0.5)

fit_DecreaseBoot1 <- bootnet(fit_DecreaseBoot, nBoots = 1000, nCores = 8,type = "case")

fit_StableBoot1 <- bootnet(fit_StableBoot, nBoots = 1000, nCores = 8,type = "case")

fit_IncreaseBoot1 <- bootnet(fit_IncreaseBoot, nBoots = 1000, nCores = 8,type = "case")
pdf("CentralityStability.pdf")
plot(fit_DecreaseBoot1)
## Warning in plot.bootnet(fit_DecreaseBoot1): Statistic closeness does not
## contain any variance and is therefore not shown.
plot(fit_StableBoot1)
## Warning in plot.bootnet(fit_StableBoot1): Statistic closeness does not
## contain any variance and is therefore not shown.
plot(fit_IncreaseBoot1)
## Warning in plot.bootnet(fit_IncreaseBoot1): Statistic closeness does not
## contain any variance and is therefore not shown.
dev.off()
## png
##   2
```

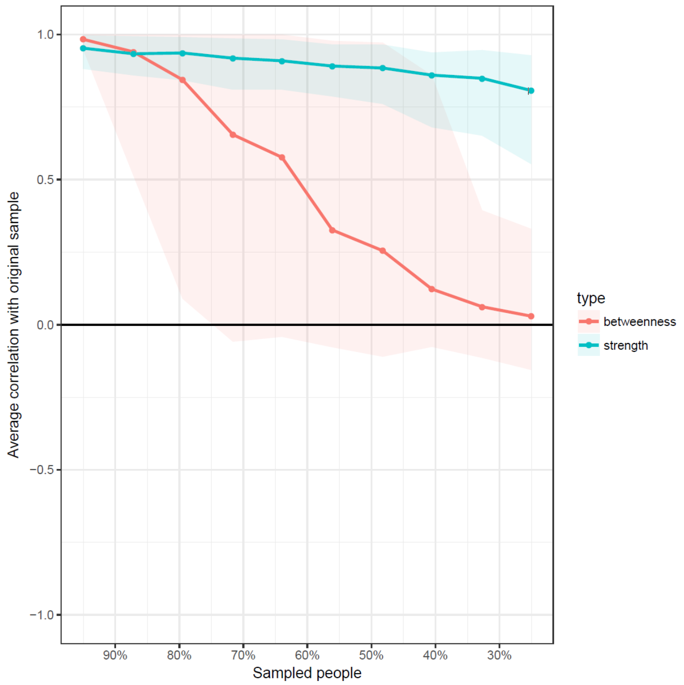

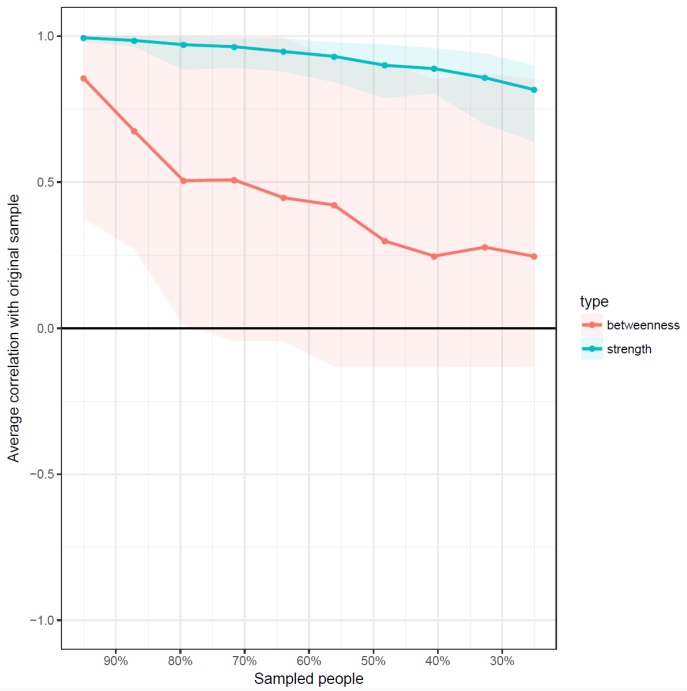

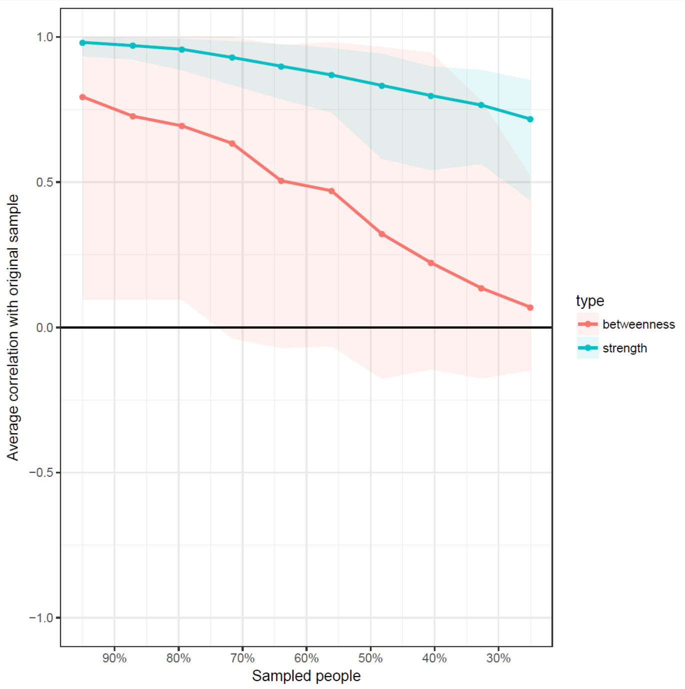


```
corStability(fit_DecreaseBoot1)
## === Correlation Stability Analysis ===
##
## Sampling levels tested:
##    nPerson Drop%   n
## 1      121  74.9  96
## 2      158  67.3  84
## 3      196  59.4 109
## 4      233  51.8 127
## 5      271  43.9 114
## 6      309  36.0 102
## 7      346  28.4  93
```

```
## 8      384  20.5  99
## 9      421  12.8  93
## 10     459   5.0  83
##
## Maximum drop proportions to retain correlation of 0.7 in at least 95% of the samples:
##
## betweenness: 0.05
##   - For more accuracy, run bootnet(..., caseMin = 0, caseMax = 0.128)
##
## closeness: 0
##   - For more accuracy, run bootnet(..., caseMin = 0, caseMax = 0.05)
##
## strength: 0.673
##   - For more accuracy, run bootnet(..., caseMin = 0.594, caseMax = 0.749)
##
## Accuracy can also be increased by increasing both 'nBoots' and 'caseN'.
```

```
corStability(fit_StableBoot1)
## === Correlation Stability Analysis ===
##
## Sampling levels tested:
##    nPerson Drop%   n
## 1      121  74.9 113
## 2      158  67.3  97
## 3      196  59.4  99
## 4      233  51.8 104
## 5      271  43.9 116
## 6      309  36.0  97
## 7      346  28.4  99
## 8      384  20.5  87
## 9      421  12.8  93
## 10     459   5.0  95
##
## Maximum drop proportions to retain correlation of 0.7 in at least 95% of the samples:
##
## betweenness: 0
##   - For more accuracy, run bootnet(..., caseMin = 0, caseMax = 0.05)
##
## closeness: 0
##   - For more accuracy, run bootnet(..., caseMin = 0, caseMax = 0.05)
##
## strength: 0.749
##   - For more accuracy, run bootnet(..., caseMin = 0.673, caseMax = 1)
##
## Accuracy can also be increased by increasing both 'nBoots' and 'caseN'.
```

```
corStability(fit_IncreaseBoot1)
## === Correlation Stability Analysis ===
##
## Sampling levels tested:
##    nPerson Drop%   n
## 1      121  74.9  85
## 2      158  67.3 100
## 3      196  59.4  96
## 4      233  51.8  97
## 5      271  43.9 100
## 6      309  36.0 111
## 7      346  28.4 105
## 8      384  20.5 100
## 9      421  12.8  99
## 10     459   5.0 107
##
## Maximum drop proportions to retain correlation of 0.7 in at least 95% of the samples:
```

```
##
## betweenness: 0
##   - For more accuracy, run bootnet(..., caseMin = 0, caseMax = 0.05)
##
## closeness: 0
##   - For more accuracy, run bootnet(..., caseMin = 0, caseMax = 0.05)
##
## strength: 0.518
##   - For more accuracy, run bootnet(..., caseMin = 0.439, caseMax = 0.594)
##
## Accuracy can also be increased by increasing both 'nBoots' and 'caseN'.

fit_DecreaseBoot2 <- bootnet(fit_DecreaseBoot, nBoots = 1000, nCores = 8)

fit_StableBoot2 <- bootnet(fit_StableBoot, nBoots = 1000, nCores = 8)

fit_IncreaseBoot2 <- bootnet(fit_IncreaseBoot, nBoots = 1000, nCores = 8)

# See Pdf for edge stability
pdf("EdgeStability.pdf", height = 25)
plot(fit_DecreaseBoot2, labels = TRUE, order = "sample")
plot(fit_StableBoot2, labels = TRUE, order = "sample")
plot(fit_IncreaseBoot2, labels = TRUE, order = "sample")
dev.off()
## png
##   2

pdf("EdgeSignificance.pdf")
plot(fit_DecreaseBoot2, "edge",plot = "difference",onlyNonZero = TRUE, order = "sample")
## Expected significance level given number of bootstrap samples is approximately: 0.05
plot(fit_StableBoot2, "edge", plot = "difference",onlyNonZero = TRUE, order = "sample")
## Expected significance level given number of bootstrap samples is approximately: 0.05
plot(fit_IncreaseBoot2, "edge", plot = "difference",onlyNonZero = TRUE, order = "sample")
## Expected significance level given number of bootstrap samples is approximately: 0.05
dev.off()
## png
##   2
```

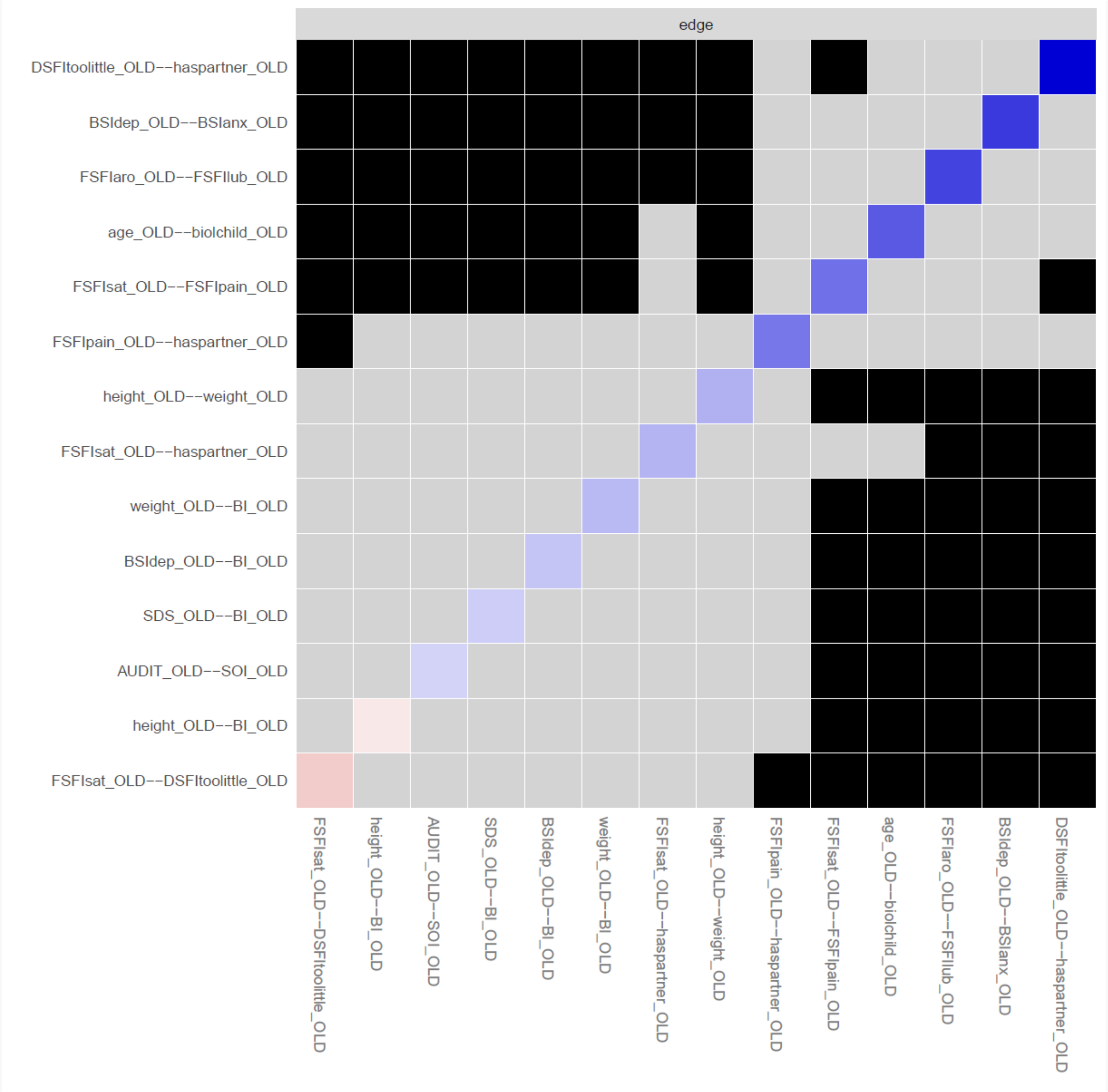
edge
DSFItoolittle_OLD--haspartner_OLD
BSIdep_OLD--BSIanx_OLD
FSFIaro_OLD--FSFIlub_OLD
age_OLD--biolchild_OLD
FSFIsat_OLD--FSFIpain_OLD
FSFIpain_OLD--haspartner_OLD
height_OLD--weight_OLD
FSFIsat_OLD--haspartner_OLD
weight_OLD--BI_OLD
BSIdep_OLD--BI_OLD
SDS_OLD--BI_OLD
AUDIT_OLD--SOI_OLD
height_OLD--BI_OLD
FSFIsat_OLD--DSFItoolittle_OLD

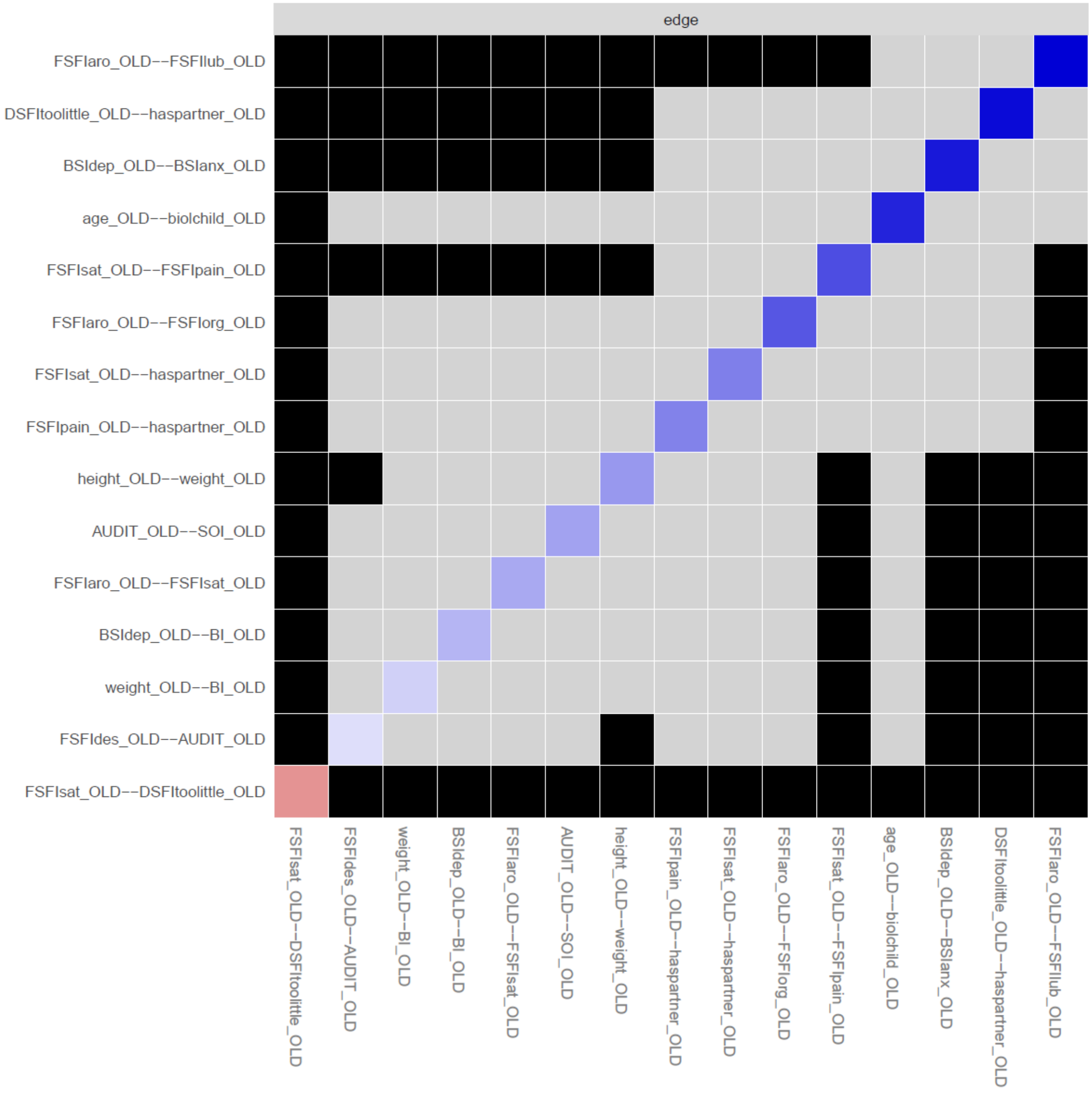
edge
FSFIaro_OLD−−FSFIlub_OLD
DSFItoolittle_OLD−−haspartner_OLD
BSIdep_OLD−−BSIanx_OLD
age_OLD−−biolchild_OLD
FSFIsat_OLD−−FSFIpain_OLD
FSFIaro_OLD−−FSFIorg_OLD
FSFIsat_OLD−−haspartner_OLD
FSFIpain_OLD−−haspartner_OLD
height_OLD−−weight_OLD
AUDIT_OLD−−SOI_OLD
FSFIaro_OLD−−FSFIsat_OLD
BSIdep_OLD−−BI_OLD
weight_OLD−−BI_OLD
FSFIdes_OLD−−AUDIT_OLD
FSFIsat_OLD−−DSFItoolittle_OLD

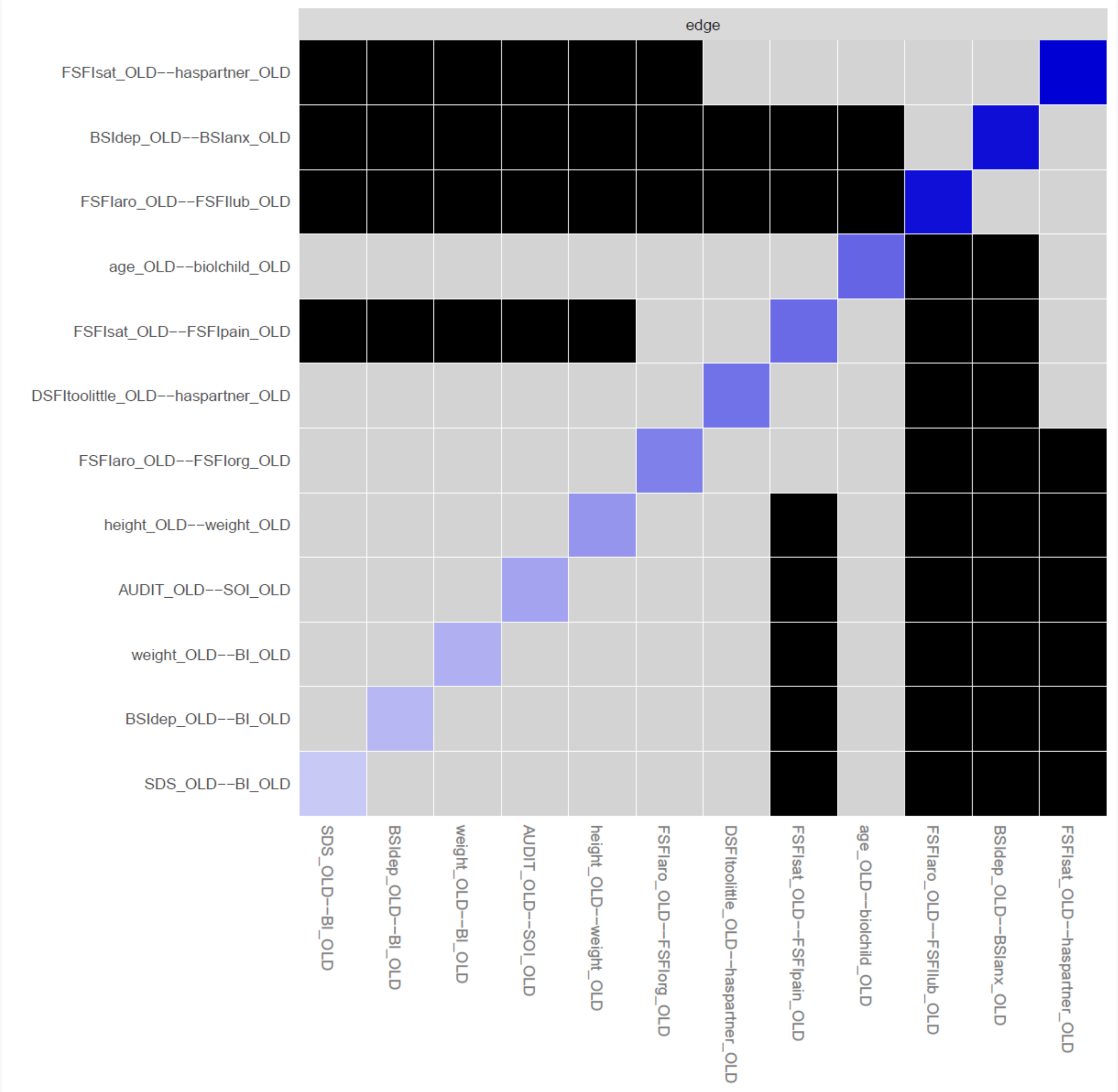


```
pdf("StrengthSignificance.pdf")
plot(fit_DecreaseBoot2, "strength", plot = "difference", order = "sample")
## Expected significance level given number of bootstrap samples is approximately: 0.05
plot(fit_StableBoot2, "strength", plot = "difference", order = "sample")
## Expected significance level given number of bootstrap samples is approximately: 0.05
plot(fit_IncreaseBoot2, "strength", plot = "difference", order = "sample")
## Expected significance level given number of bootstrap samples is approximately: 0.05
dev.off()
## png
##   2
```

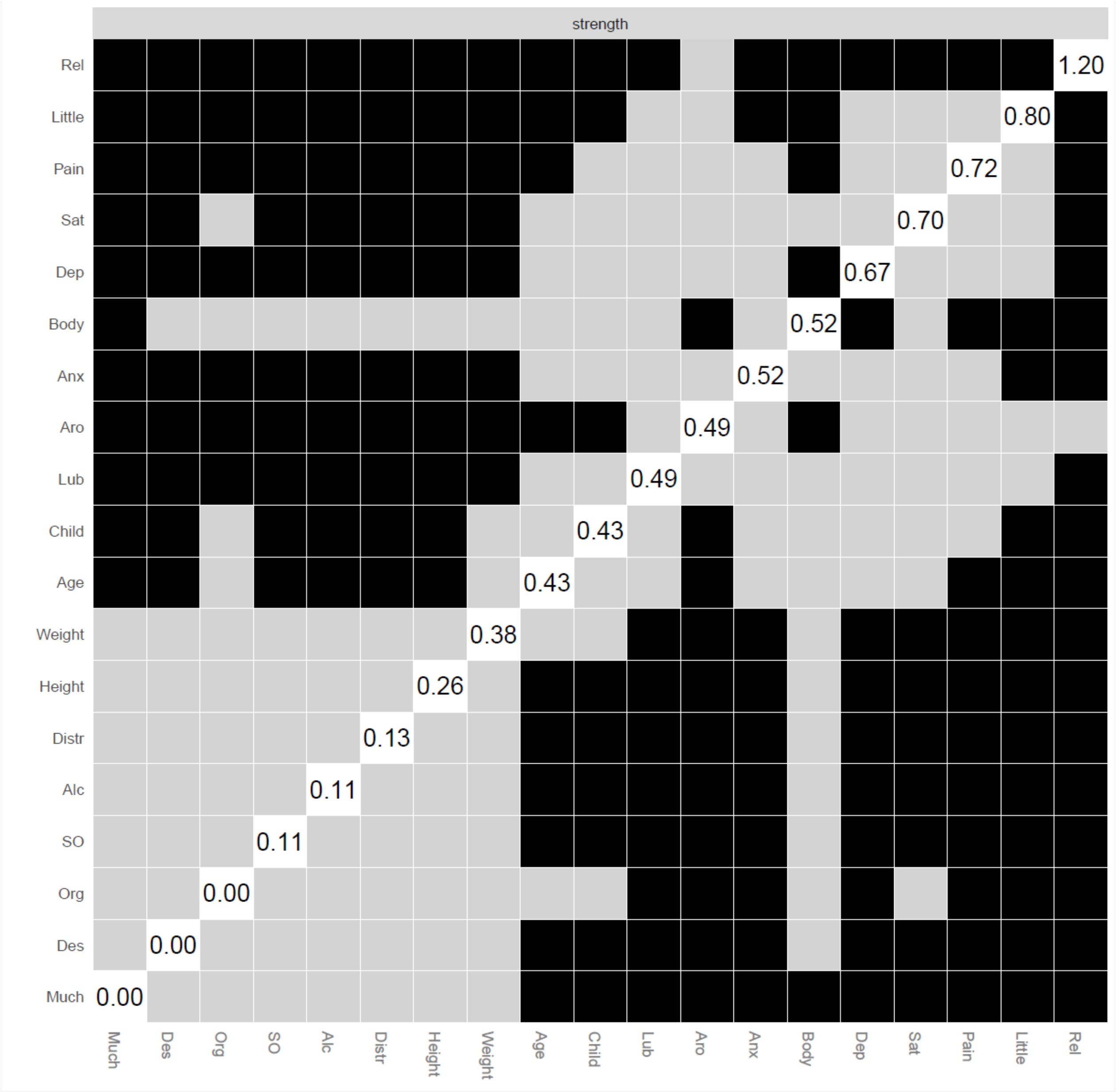
strength
Rel
Little
Pain
Sat
Dep
Body
Anx
Aro
Lub
Child
Age
Weight
Height
Distr
Alc
SO
Org
Des
Much
1.20
0.80
0.72
0.70
0.67
0.52
0.52
0.49
0.49
0.43
0.43
0.38
0.26
0.13
0.11
0.11
0.00
0.00
0.00
Much
Des
Org
SO
Alc
Distr
Height
Weight
Age
Child
Lub
Aro
Anx
Body
Dep
Sat
Pain
Little
Rel

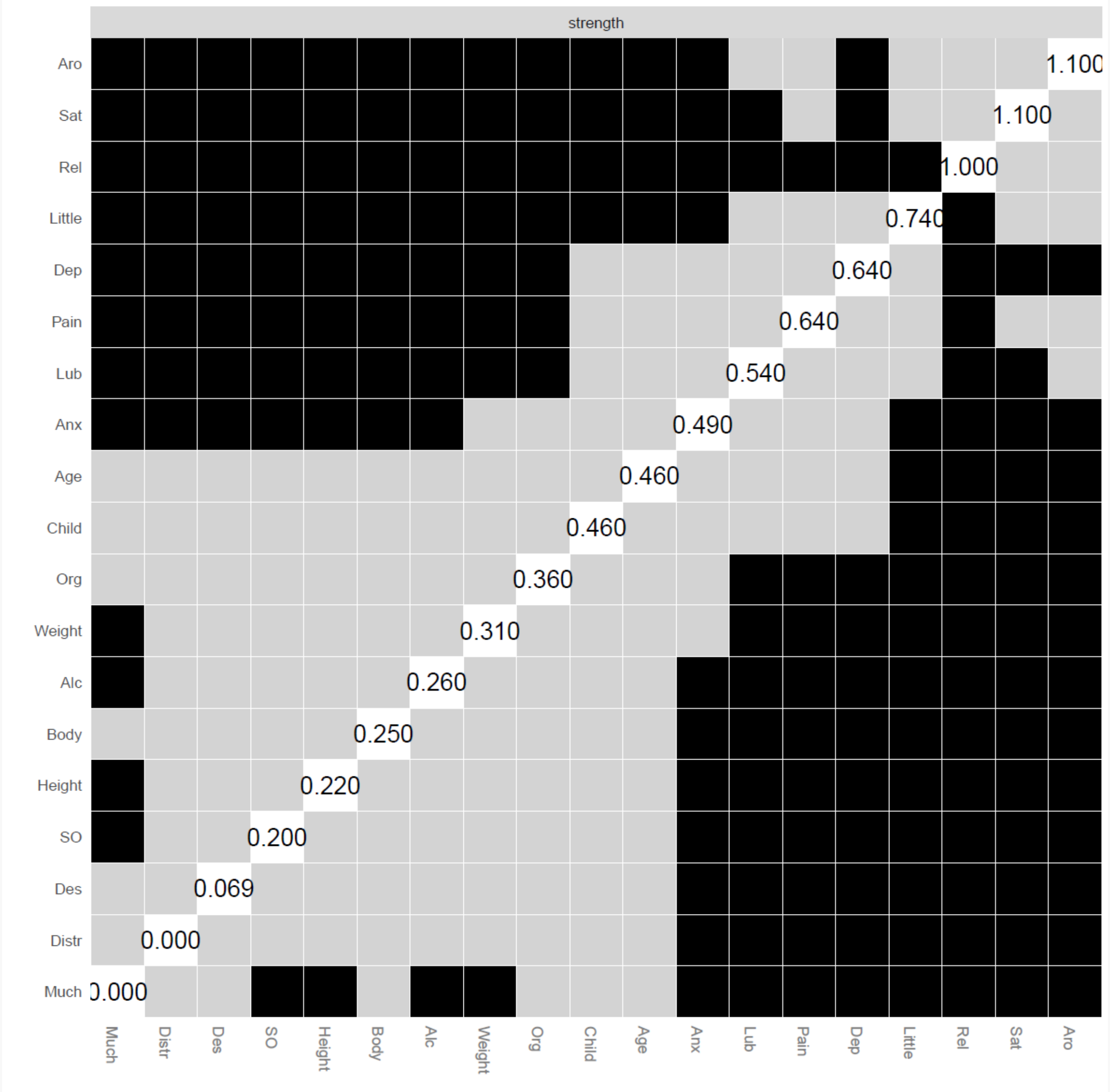
strength
Aro
Sat
Rel
Little
Dep
Pain
Lub
Anx
Age
Child
Org
Weight
Alc
Body
Height
SO
Des
Distr
Much
1.100
1.100
1.000
0.740
0.640
0.640
0.540
0.490
0.460
0.460
0.360
0.310
0.260
0.250
0.220
0.200
0.069
0.000
0.000
Much
Distr
Des
SO
Height
Body
Alc
Weight
Org
Child
Age
Anx
Lub
Pain
Dep
Little
Rel
Sat
Aro

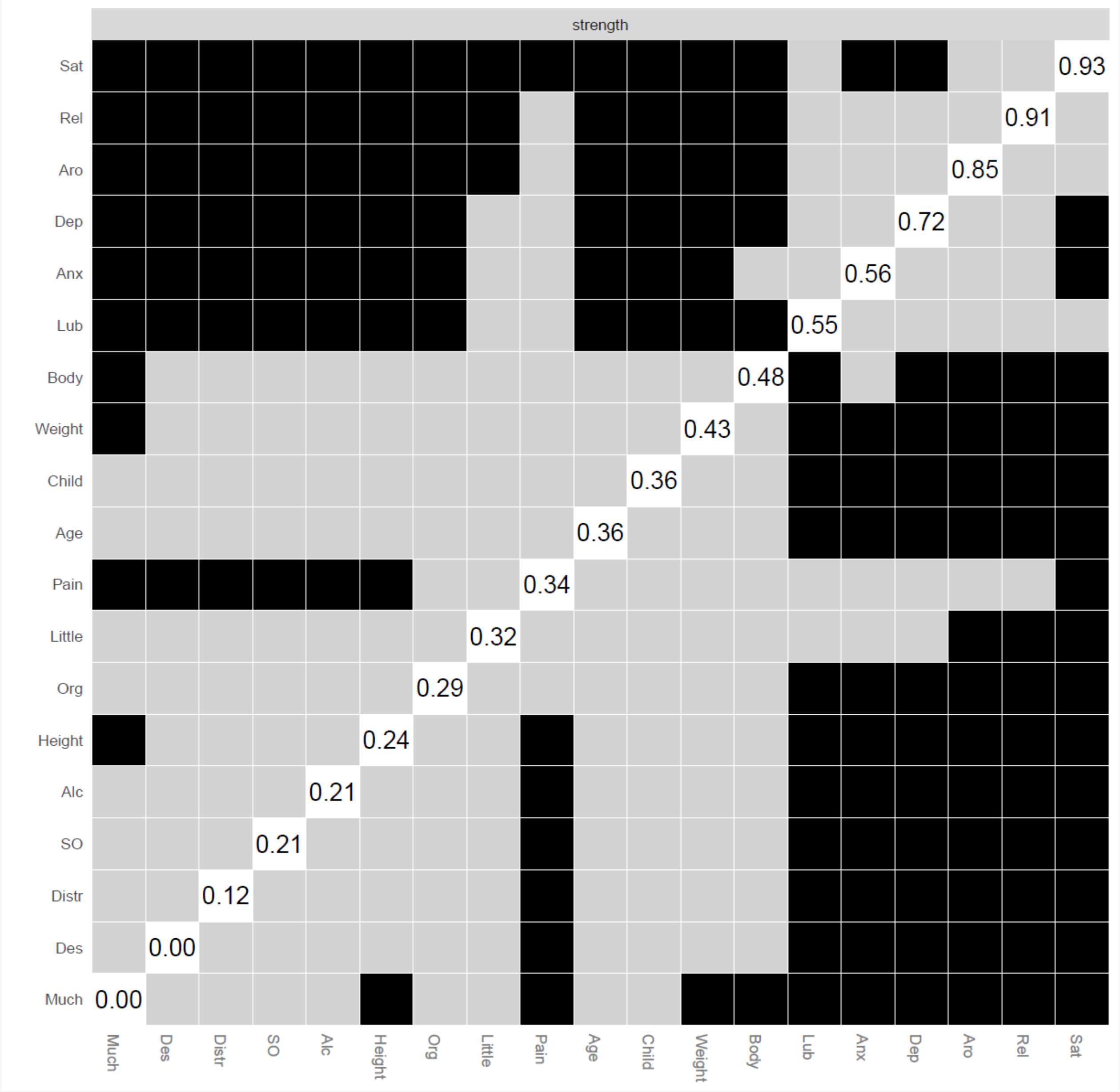

9. Network Comparison Test

```
#########################################################################################
# NCT
library(NetworkComparisonTest)
NCTDecStab <- NCT(DecreaseRS, StableRS, gamma = 0.5, it = 1000, binary.data=FALSE,
paired=FALSE, weighted=TRUE, progressbar=TRUE)
NCTDecStab$nwinv.pval
## [1] 0.56

NCTDecStab$glstrinv.real
## [1] 1.426189
NCTDecStab$glstrinv.pval
## [1] 0.071

NCTIncStab <- NCT(StableRS, IncreaseRS, gamma = 0.5, it = 1000, binary.data=FALSE,
paired=FALSE, weighted=TRUE, progressbar=TRUE)
NCTIncStab$nwinv.pval
```

```
## [1] 0.603

NCTIncStab$glstrinv.real
## [1] 0.0696736
NCTIncStab$glstrinv.pval
## [1] 0.938

NCTDecInc <- NCT(DecreaseRS, IncreaseRS, gamma = 0.5, it = 1000, binary.data=FALSE,
paired=FALSE, weighted=TRUE, progressbar=TRUE)
NCTDecInc$nwinv.pval
## [1] 0.245

NCTDecInc$glstrinv.real
## [1] 1.356516
NCTDecInc$glstrinv.pval
## [1] 0.081
# no significant differences, but presumably because of power

###########################################################################################
mean(as.matrix(clustcoef_auto(qDecrease2)))
## [1] 0.07866428
mean(as.matrix(clustcoef_auto(qStable2)))
## [1] 0.0504125
mean(as.matrix(clustcoef_auto(qIncrease2)))
## [1] 0.09442654
###########################################################################################
```

10. Network Communities

```
# CLUSTER ANALYSIS

#### DECREASE
library(igraph)
NetworkDecreaseIgraph <- as.igraph(qDecrease2)

noClusterDecrease <- 0
for (i in 1:1000){
  communityWTDecrease <- cluster_walktrap(NetworkDecreaseIgraph,
                                  weights = E(NetworkDecreaseIgraph)$weight,
                                  steps = i,
                                  merges = TRUE,
                                  modularity = TRUE,
                                  membership = TRUE)
  noClusterDecrease[i] <- length(communityWTDecrease)
}

plot(noClusterDecrease)
```

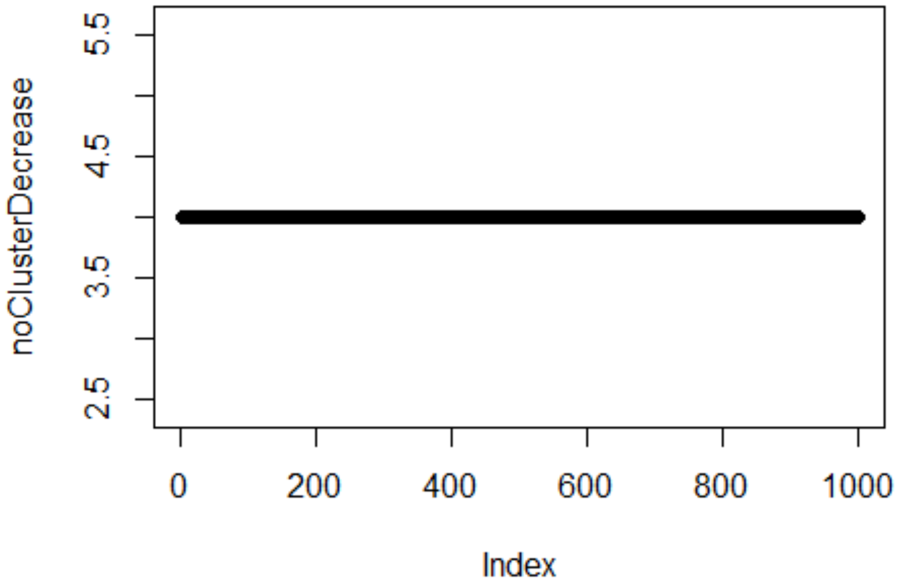


```r
median(noClusterDecrease)
## [1] 4
mean(noClusterDecrease)
## [1] 4

communityWTPlotDecrease <- cluster_walktrap(NetworkDecreaseIgraph,
                                            weights = E(NetworkDecreaseIgraph)$weight,
                                            steps = 200,
                                            merges = TRUE,
                                            modularity = TRUE,
                                            membership = TRUE)

plot(communityWTPlotDecrease, NetworkDecreaseIgraph, layout = qDecrease2$layout)
```

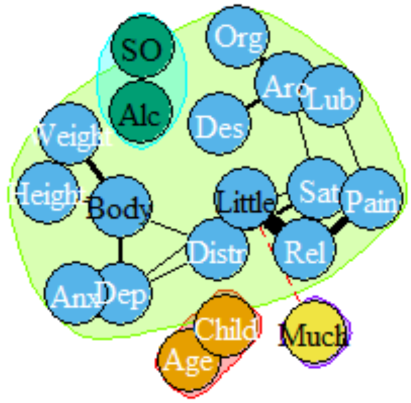


```r
# STABLE
NetworkStableIgraph <- as.igraph(qStable2)

noClusterStable <- 0
for (i in 1:1000){
  communityWTStable <- cluster_walktrap(NetworkStableIgraph,
```

```
                                  weights = E(NetworkStableIgraph)$weight,
                                  steps = i,
                                  merges = TRUE,
                                  modularity = TRUE,
                                  membership = TRUE)
  noClusterStable[i] <- length(communityWTStable)
}

plot(noClusterStable[0:1000])
```

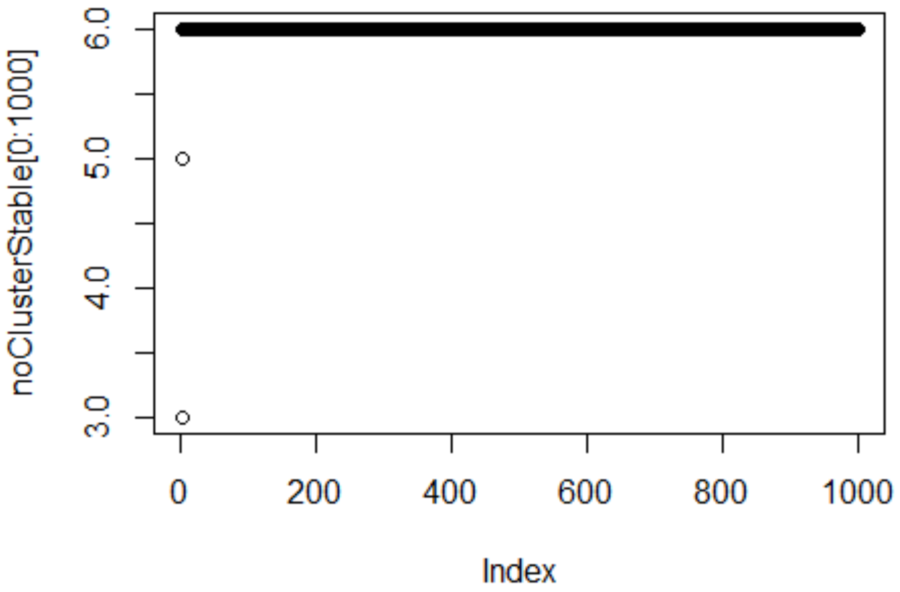


```
median(noClusterStable[0:1000])
## [1] 6
mean(noClusterStable[0:1000])
## [1] 5.996

communityWTPlotStable <- cluster_walktrap(NetworkStableIgraph,
                                    weights = E(NetworkStableIgraph)$weight,
                                    steps = 200,
                                    merges = TRUE,
                                    modularity = TRUE,
                                    membership = TRUE)

plot(communityWTPlotStable, NetworkStableIgraph, layout = qStable2$layout)
```

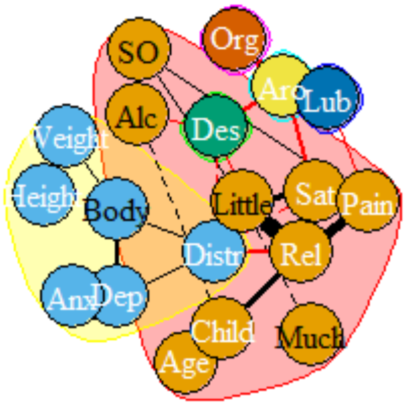


```
#### Increase
NetworkIncreaseIgraph <- as.igraph(qIncrease2)

noClusterIncrease <- 0
for (i in 1:1000){
  communityWTIncrease <- cluster_walktrap(NetworkIncreaseIgraph,
                                  weights = E(NetworkIncreaseIgraph)$weight,
                                  steps = i,
                                  merges = TRUE,
                                  modularity = TRUE,
                                  membership = TRUE)
  noClusterIncrease[i] <- length(communityWTIncrease)
}

plot(noClusterIncrease[0:1000])
```

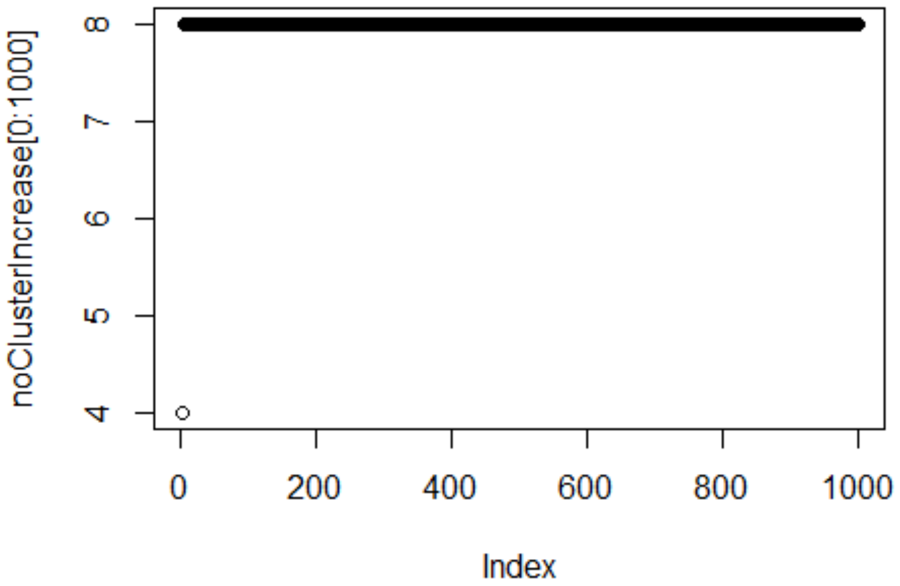


```
median(noClusterIncrease[0:1000])
## [1] 8
mean(noClusterIncrease[0:1000])
## [1] 7.988
```

```
communityWTPlotIncrease <- cluster_walktrap(NetworkIncreaseIgraph,
                                     weights = E(NetworkIncreaseIgraph)$weight,
                                     steps = 200,
                                     merges = TRUE,
                                     modularity = TRUE,
                                     membership = TRUE)

plot(communityWTPlotIncrease, NetworkIncreaseIgraph, layout = qIncrease2$layout)
```

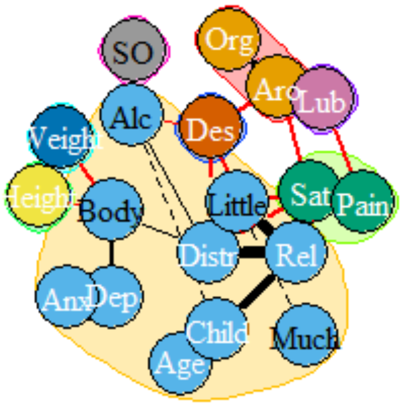


```
pdf("WalktrapCluster.pdf")
plot(communityWTPlotDecrease, NetworkDecreaseIgraph, layout = qDecrease2$layout)
plot(communityWTPlotStable, NetworkStableIgraph, layout = qStable2$layout)
plot(communityWTPlotIncrease, NetworkIncreaseIgraph, layout = qIncrease2$layout)
dev.off()
## png
##   2
```

11. Replication – Data Importation

```
##########################################################################
# NEW DATASET                                                            #
##########################################################################

DataNetworkNEW <- FSFI_Gunstetal_Imputed_NegTo0[,23:42]
colnames(DataNetworkNEW)
##  [1] "age_NEW"           "height_NEW"        "weight_NEW"
##  [4] "hc_NEW"            "biolchild_NEW"     "FSFIdes_NEW"
##  [7] "FSFIaro_NEW"       "FSFIlub_NEW"       "FSFIorg_NEW"
## [10] "FSFIsat_NEW"       "FSFIpain2_NEW"     "SDS_NEW"
## [13] "BSIdep_NEW"        "BSIanx_NEW"        "BI_NEW"
## [16] "AUDIT_NEW"         "DSFItoomuch_NEW"   "DSFItoolittle_NEW"
## [19] "SOI_NEW"           "haspartner_NEW"
colnames(DataNetwork)
##  [1] "age_OLD"           "height_OLD"        "weight_OLD"
##  [4] "biolchild_OLD"     "FSFIdes_OLD"       "FSFIaro_OLD"
##  [7] "FSFIlub_OLD"       "FSFIorg_OLD"       "FSFIsat_OLD"
## [10] "FSFIpain_OLD"      "SDS_OLD"           "BSIdep_OLD"
## [13] "BSIanx_OLD"        "BI_OLD"            "AUDIT_OLD"
## [16] "DSFItoolittle_OLD" "DSFItoomuch_OLD"   "SOI_OLD"
## [19] "haspartner_OLD"    "SDI_groups"        "SDI_change"
DSFItoolittle_NEW <- c()
DSFItoolittle_NEW <- DataNetworkNEW[,18]
DataNetworkNEW[,18] <- DataNetworkNEW[,17]
DataNetworkNEW[,17] <- DSFItoolittle_NEW
rm(DSFItoolittle_NEW)

colnames(DataNetworkNEW)[17] <- "DSFItoolittle_NEW"
```

```
colnames(DataNetworkNEW)[18] <- "DSFItoomuch_NEW"
colnames(DataNetworkNEW)
##  [1] "age_NEW"           "height_NEW"        "weight_NEW"
##  [4] "hc_NEW"            "biolchild_NEW"     "FSFIdes_NEW"
##  [7] "FSFIaro_NEW"       "FSFIlub_NEW"       "FSFIorg_NEW"
## [10] "FSFIsat_NEW"       "FSFIpain2_NEW"     "SDS_NEW"
## [13] "BSIdep_NEW"        "BSIanx_NEW"        "BI_NEW"
## [16] "AUDIT_NEW"         "DSFItoolittle_NEW" "DSFItoomuch_NEW"
## [19] "SOI_NEW"           "haspartner_NEW"
DataNetworkNEW[,21] <- FSFI_Gunstetal_Imputed_NegTo0$SDI_groups
colnames(DataNetworkNEW)[21] <- "SDI_groups"

DataNetworkNEW[,5] <- round(DataNetworkNEW[,5]) # adjust children to count variable

apply(apply(DataNetworkNEW, 2, is.na), 2, sum) # no NA
##           age_NEW        height_NEW        weight_NEW            hc_NEW
##                 0                 0                 0                 0
##     biolchild_NEW       FSFIdes_NEW       FSFIaro_NEW       FSFIlub_NEW
##                 0                 0                 0                 0
##       FSFIorg_NEW       FSFIsat_NEW     FSFIpain2_NEW           SDS_NEW
##                 0                 0                 0                 0
##        BSIdep_NEW        BSIanx_NEW            BI_NEW         AUDIT_NEW
##                 0                 0                 0                 0
## DSFItoolittle_NEW   DSFItoomuch_NEW           SOI_NEW    haspartner_NEW
##                 0                 0                 0                 0
##        SDI_groups
##                 0
DecreaseNEW <- subset(DataNetworkNEW, SDI_groups==1, select=age_NEW:haspartner_NEW)
StableNEW <- subset(DataNetworkNEW, SDI_groups==2, select=age_NEW:haspartner_NEW)
IncreaseNEW <- subset(DataNetworkNEW, SDI_groups==3, select=age_NEW:haspartner_NEW)
nrow(DecreaseNEW) + nrow(StableNEW) + nrow(IncreaseNEW)
## [1] 1692
library(psych)

describe(DecreaseNEW)
##                   vars   n   mean    sd median trimmed   mad   min   max
## age_NEW              1 522  33.21  4.85   33.0   32.90  5.93  25.0  51.0
## height_NEW           2 522 166.03  6.45  166.0  166.01  5.93 145.0 182.0
## weight_NEW           3 522  68.38 15.57   65.0   66.36 11.86  44.0 164.0
## hc_NEW               4 522   0.40  0.49    0.0    0.37  0.00   0.0   1.0
## biolchild_NEW        5 522   1.15  1.27    1.0    0.98  1.48   0.0   9.0
## FSFIdes_NEW          6 522   2.67  0.95    2.4    2.63  0.89   1.2   5.4
## FSFIaro_NEW          7 522   3.92  1.88    4.5    4.15  1.33   0.0   6.0
## FSFIlub_NEW          8 522   4.62  2.04    5.7    5.02  0.44   0.0   6.0
## FSFIorg_NEW          9 522   4.00  2.09    4.8    4.24  1.78   0.0   6.0
## FSFIsat_NEW         10 522   4.12  1.84    4.8    4.33  1.78   0.4   6.0
## FSFIpain2_NEW       11 522   4.33  2.25    5.4    4.66  0.89   0.0   6.0
## SDS_NEW             12 522   8.75  6.38    8.0    8.36  7.41   0.0  28.0
## BSIdep_NEW          13 522   4.50  4.74    3.0    3.69  2.97   0.0  23.0
## BSIanx_NEW          14 522   3.22  3.74    2.0    2.51  2.97   0.0  21.0
## BI_NEW              15 522  26.80  6.77   27.0   26.67  7.41  11.0  48.0
## AUDIT_NEW           16 522   5.08  3.87    4.0    4.58  2.97   0.0  23.0
## DSFItoolittle_NEW   17 522   3.44  3.97    2.0    2.71  2.97   0.0  25.0
## DSFItoomuch_NEW     18 522   0.18  0.69    0.0    0.00  0.00   0.0   8.0
## SOI_NEW             19 522  71.81 34.06   64.0   65.81 17.79  20.0 211.0
## haspartner_NEW      20 522   0.91  0.29    1.0    1.00  0.00   0.0   1.0
##                   range  skew kurtosis   se
## age_NEW            26.0  0.67     0.38 0.21
## height_NEW         37.0 -0.03    -0.06 0.28
## weight_NEW        120.0  2.15     8.15 0.68
## hc_NEW              1.0  0.42    -1.83 0.02
## biolchild_NEW       9.0  1.32     3.26 0.06
## FSFIdes_NEW         4.2  0.34    -0.11 0.04
```

```
## FSFIaro_NEW         6.0 -1.03    -0.16 0.08
## FSFIlub_NEW         6.0 -1.49     0.70 0.09
## FSFIorg_NEW         6.0 -0.83    -0.72 0.09
## FSFIsat_NEW         5.6 -0.77    -0.79 0.08
## FSFIpain2_NEW       6.0 -1.12    -0.35 0.10
## SDS_NEW            28.0  0.44    -0.69 0.28
## BSIdep_NEW         23.0  1.45     1.83 0.21
## BSIanx_NEW         21.0  1.84     3.64 0.16
## BI_NEW             37.0  0.17    -0.33 0.30
## AUDIT_NEW          23.0  1.32     1.98 0.17
## DSFItoolittle_NEW  25.0  1.66     2.99 0.17
## DSFItoomuch_NEW     8.0  5.64    42.70 0.03
## SOI_NEW           191.0  1.85     3.49 1.49
## haspartner_NEW      1.0 -2.82     5.94 0.01

describe(StableNEW)
##                   vars   n   mean    sd median trimmed   mad   min max
## age_NEW              1 687  32.92  4.89   33.0   32.57  4.45  25.0  53
## height_NEW           2 687 165.99  6.06  166.0  165.87  5.93 143.0 186
## weight_NEW           3 687  66.46 13.70   64.0   64.71 10.38  38.5 175
## hc_NEW               4 687   0.39  0.49    0.0    0.37  0.00   0.0   1
## biolchild_NEW        5 687   1.08  1.29    1.0    0.89  1.48   0.0   9
## FSFIdes_NEW          6 687   2.94  0.97    3.0    2.91  0.89   1.2   6
## FSFIaro_NEW          7 687   4.12  1.83    4.8    4.41  0.89   0.0   6
## FSFIlub_NEW          8 687   4.71  2.00    5.7    5.13  0.44   0.0   6
## FSFIorg_NEW          9 687   4.16  2.02    4.8    4.44  1.78   0.0   6
## FSFIsat_NEW         10 687   4.16  1.84    4.8    4.38  1.78   0.4   6
## FSFIpain2_NEW       11 687   4.48  2.21    6.0    4.85  0.00   0.0   6
## SDS_NEW             12 687   8.56  5.87    8.0    8.14  5.93   0.0  28
## BSIdep_NEW          13 687   4.67  4.48    3.0    4.00  2.97   0.0  23
## BSIanx_NEW          14 687   3.40  3.90    2.0    2.68  2.97   0.0  24
## BI_NEW              15 687  26.14  6.75   26.0   25.99  7.41  12.0  44
## AUDIT_NEW           16 687   5.18  4.01    4.0    4.67  2.97   0.0  30
## DSFItoolittle_NEW   17 687   4.00  4.57    3.0    3.19  4.45   0.0  28
## DSFItoomuch_NEW     18 687   0.20  0.80    0.0    0.00  0.00   0.0   8
## SOI_NEW             19 687  72.82 38.20   62.0   65.12 17.79  24.0 261
## haspartner_NEW      20 687   0.84  0.37    1.0    0.92  0.00   0.0   1
##                   range  skew kurtosis   se
## age_NEW            28.0  0.80     0.93 0.19
## height_NEW         43.0  0.11     0.13 0.23
## weight_NEW        136.5  1.89     7.44 0.52
## hc_NEW              1.0  0.44    -1.81 0.02
## biolchild_NEW       9.0  1.41     3.37 0.05
## FSFIdes_NEW         4.8  0.33    -0.05 0.04
## FSFIaro_NEW         6.0 -1.25     0.37 0.07
## FSFIlub_NEW         6.0 -1.60     1.04 0.08
## FSFIorg_NEW         6.0 -0.99    -0.37 0.08
## FSFIsat_NEW         5.6 -0.84    -0.65 0.07
## FSFIpain2_NEW       6.0 -1.28     0.01 0.08
## SDS_NEW            28.0  0.59    -0.17 0.22
## BSIdep_NEW         23.0  1.31     1.47 0.17
## BSIanx_NEW         24.0  1.81     3.74 0.15
## BI_NEW             32.0  0.18    -0.67 0.26
## AUDIT_NEW          30.0  1.73     4.96 0.15
## DSFItoolittle_NEW  28.0  1.49     2.02 0.17
## DSFItoomuch_NEW     8.0  5.22    31.59 0.03
## SOI_NEW           237.0  2.12     4.50 1.46
## haspartner_NEW      1.0 -1.83     1.37 0.01

describe(IncreaseNEW)
##                   vars   n   mean    sd median trimmed   mad    min   max
## age_NEW              1 483  33.13  4.94   32.0   32.84  5.93  25.00  52.0
## height_NEW           2 483 166.42  6.27  166.5  166.38  5.39 150.00 186.0
```

```
## weight_NEW           3 483  67.74 15.00   65.0    65.68 10.38  42.00 166.0
## hc_NEW               4 483   0.35  0.48    0.0     0.32  0.00   0.00   1.0
## biolchild_NEW        5 483   1.12  1.39    1.0     0.93  1.48   0.00  12.0
## FSFIdes_NEW          6 483   3.40  1.02    3.6     3.40  0.89   1.20   5.4
## FSFIaro_NEW          7 483   4.52  1.69    5.1     4.87  0.89   0.00   6.0
## FSFIlub_NEW          8 483   5.08  1.73    6.0     5.56  0.00   0.00   6.0
## FSFIorg_NEW          9 483   4.46  1.90    5.2     4.81  1.19   0.00   6.0
## FSFIsat_NEW         10 483   4.23  1.88    4.8     4.47  1.78   0.40   6.0
## FSFIpain2_NEW       11 483   4.64  2.23    6.0     5.05  0.00   0.00   6.0
## SDS_NEW             12 483   7.90  5.95    8.0     7.40  5.93   0.00  28.0
## BSIdep_NEW          13 483   4.77  4.42    4.0     4.14  4.45   0.00  22.0
## BSIanx_NEW          14 483   3.33  3.86    2.0     2.61  2.97   0.00  22.0
## BI_NEW              15 483  25.34  7.26   25.0    25.07  7.41  11.00  47.0
## AUDIT_NEW           16 483   5.62  4.22    5.0     5.05  2.97   0.00  30.0
## DSFItoolittle_NEW   17 483   5.24  5.05    4.0     4.55  4.45   0.00  27.0
## DSFItoomuch_NEW     18 483   0.06  0.34    0.0     0.00  0.00   0.00   4.0
## SOI_NEW             19 483  78.76 44.42   65.0    70.58 23.72  15.04 252.0
## haspartner_NEW      20 483   0.77  0.42    1.0     0.84  0.00   0.00   1.0
##                    range  skew kurtosis   se
## age_NEW            27.00  0.65     0.40 0.22
## height_NEW         36.00  0.07    -0.01 0.29
## weight_NEW        124.00  1.91     6.03 0.68
## hc_NEW              1.00  0.61    -1.63 0.02
## biolchild_NEW      12.00  1.82     7.66 0.06
## FSFIdes_NEW         4.20  0.09    -0.43 0.05
## FSFIaro_NEW         6.00 -1.62     1.74 0.08
## FSFIlub_NEW         6.00 -2.21     3.61 0.08
## FSFIorg_NEW         6.00 -1.24     0.30 0.09
## FSFIsat_NEW         5.60 -0.89    -0.63 0.09
## FSFIpain2_NEW       6.00 -1.42     0.32 0.10
## SDS_NEW            28.00  0.70     0.19 0.27
## BSIdep_NEW         22.00  1.18     1.04 0.20
## BSIanx_NEW         22.00  1.88     4.06 0.18
## BI_NEW             36.00  0.34    -0.29 0.33
## AUDIT_NEW          30.00  1.51     3.14 0.19
## DSFItoolittle_NEW  27.00  1.19     1.13 0.23
## DSFItoomuch_NEW     4.00  7.24    61.45 0.02
## SOI_NEW           236.96  1.71     2.40 2.02
## haspartner_NEW      1.00 -1.29    -0.33 0.02
```

12. Replication – Network Estimation (incl. hormonal contraception)

```
#########################
# 4. Network estimation #
#########################

VarDomain <- c("g","g","g","c","p","g","g",
               "g","g","g","g","g","g","g",
               "g","g","g","g","g","c")
VarLevel <- c(1,1,1,2,1,1,1,
              1,1,1,1,1,1,1,
              1,1,1,1,1,2)

library(mgm)

fit_DecreaseNEW <- mgm(data = as.matrix(DecreaseNEW),
                       type = VarDomain,
                       level = VarLevel,
                       k = 2,
                       lambdaSel = 'EBIC',
                       lambdaGam = 0.5) # hyper parameter
```

```
fit_StableNEW <- mgm(data = as.matrix(StableNEW),
                     type = VarDomain,
                     level = VarLevel,
                     k = 2,
                     lambdaSel = 'EBIC',
                     lambdaGam = 0.5) # hyper parameter

fit_IncreaseNEW <- mgm(data = as.matrix(IncreaseNEW),
                       type = VarDomain,
                       level = VarLevel,
                       k = 2,
                       lambdaSel = 'EBIC',
                       lambdaGam = 0.5) # hyper parameter

fit_DecreaseNEW$pairwise$wadj[,4]
##  [1] 0 0 0 0 0 0 0 0 0 0 0 0 0 0 0 0 0 0 0 0
fit_StableNEW$pairwise$wadj[,4]
##  [1] 0 0 0 0 0 0 0 0 0 0 0 0 0 0 0 0 0 0 0 0
fit_IncreaseNEW$pairwise$wadj[,4] # HC has no edge with anything again
##  [1] 0 0 0 0 0 0 0 0 0 0 0 0 0 0 0 0 0 0 0 0
```

13. Replication – Network Estimation (excl. hormonal contraception)

```
#########
# No HC #
#########
DataNetworkNEW <- DataNetworkNEW[,-4] # get rid off HC
colnames(DataNetworkNEW)
##  [1] "age_NEW"           "height_NEW"        "weight_NEW"
##  [4] "biolchild_NEW"     "FSFIdes_NEW"       "FSFIaro_NEW"
##  [7] "FSFIlub_NEW"       "FSFIorg_NEW"       "FSFIsat_NEW"
## [10] "FSFIpain2_NEW"     "SDS_NEW"           "BSIdep_NEW"
## [13] "BSIanx_NEW"        "BI_NEW"            "AUDIT_NEW"
## [16] "DSFItoolittle_NEW" "DSFItoomuch_NEW"   "SOI_NEW"
## [19] "haspartner_NEW"    "SDI_groups"
DecreaseNEW <- subset(DataNetworkNEW, SDI_groups==1, select=age_NEW:haspartner_NEW)
StableNEW <- subset(DataNetworkNEW, SDI_groups==2, select=age_NEW:haspartner_NEW)
IncreaseNEW <- subset(DataNetworkNEW, SDI_groups==3, select=age_NEW:haspartner_NEW)
nrow(DecreaseNEW) + nrow(StableNEW) + nrow(IncreaseNEW)
## [1] 1692

##################################
# Random Subsamples of same size #
##################################
set.seed(17)
nrow(DecreaseNEW)
## [1] 522
nrow(StableNEW)
## [1] 687
nrow(IncreaseNEW) # 483
## [1] 483
randomSample = function(df,n) {
  return (df[sample(nrow(df), n),])
}

DecreaseNEWRS <- randomSample(DecreaseNEW, n = 483)
StableNEWRS <- randomSample(StableNEW, n = 483)
IncreaseNEWRS <- IncreaseNEW

library(psych)
describe(DecreaseNEWRS)
```

```
##                   vars   n   mean    sd median trimmed   mad   min   max
## age_NEW              1 483  33.25  4.84   33.0   32.95  5.93  25.0  51.0
## height_NEW           2 483 166.13  6.39  166.0  166.07  5.93 145.0 182.0
## weight_NEW           3 483  68.38 15.13   65.0   66.47 10.38  44.0 164.0
## biolchild_NEW        4 483   1.14  1.27    1.0    0.96  1.48   0.0   9.0
## FSFIdes_NEW          5 483   2.67  0.95    2.4    2.63  0.89   1.2   5.4
## FSFIaro_NEW          6 483   3.94  1.88    4.5    4.18  1.33   0.0   6.0
## FSFIlub_NEW          7 483   4.63  2.03    5.7    5.03  0.44   0.0   6.0
## FSFIorg_NEW          8 483   4.02  2.08    4.8    4.27  1.78   0.0   6.0
## FSFIsat_NEW          9 483   4.15  1.83    4.8    4.36  1.78   0.4   6.0
## FSFIpain2_NEW       10 483   4.35  2.24    5.4    4.68  0.89   0.0   6.0
## SDS_NEW             11 483   8.61  6.41    8.0    8.17  7.41   0.0  28.0
## BSIdep_NEW          12 483   4.54  4.80    3.0    3.71  2.97   0.0  23.0
## BSIanx_NEW          13 483   3.20  3.77    2.0    2.46  2.97   0.0  21.0
## BI_NEW              14 483  26.76  6.72   27.0   26.63  7.41  11.0  48.0
## AUDIT_NEW           15 483   5.10  3.91    4.0    4.59  2.97   0.0  23.0
## DSFItoolittle_NEW   16 483   3.37  3.91    2.0    2.65  2.97   0.0  25.0
## DSFItoomuch_NEW     17 483   0.18  0.68    0.0    0.00  0.00   0.0   8.0
## SOI_NEW             18 483  72.47 34.68   64.0   66.28 17.79  20.0 211.0
## haspartner_NEW      19 483   0.91  0.29    1.0    1.00  0.00   0.0   1.0
##                   range  skew kurtosis   se
## age_NEW            26.0  0.66     0.37 0.22
## height_NEW         37.0  0.02    -0.14 0.29
## weight_NEW        120.0  2.14     8.36 0.69
## biolchild_NEW       9.0  1.40     3.62 0.06
## FSFIdes_NEW         4.2  0.33    -0.10 0.04
## FSFIaro_NEW         6.0 -1.06    -0.11 0.09
## FSFIlub_NEW         6.0 -1.50     0.74 0.09
## FSFIorg_NEW         6.0 -0.85    -0.68 0.09
## FSFIsat_NEW         5.6 -0.78    -0.77 0.08
## FSFIpain2_NEW       6.0 -1.13    -0.32 0.10
## SDS_NEW            28.0  0.49    -0.64 0.29
## BSIdep_NEW         23.0  1.47     1.82 0.22
## BSIanx_NEW         21.0  1.90     3.80 0.17
## BI_NEW             37.0  0.18    -0.28 0.31
## AUDIT_NEW          23.0  1.34     1.98 0.18
## DSFItoolittle_NEW  25.0  1.65     3.05 0.18
## DSFItoomuch_NEW     8.0  5.89    47.12 0.03
## SOI_NEW           191.0  1.83     3.31 1.58
## haspartner_NEW      1.0 -2.83     6.04 0.01
```

```
describe(StableNEWRS)
##                   vars   n   mean    sd median trimmed   mad   min   max
## age_NEW              1 483  32.78  4.75   33.0   32.48  4.45  25.0  51.0
## height_NEW           2 483 165.97  6.09  165.0  165.77  5.93 149.0 186.0
## weight_NEW           3 483  66.60 14.00   64.0   64.68 10.38  38.5 175.0
## biolchild_NEW        4 483   1.09  1.32    1.0    0.90  1.48   0.0   9.0
## FSFIdes_NEW          5 483   2.95  0.99    3.0    2.93  0.89   1.2   5.4
## FSFIaro_NEW          6 483   4.09  1.88    4.8    4.36  0.89   0.0   6.0
## FSFIlub_NEW          7 483   4.66  2.03    5.7    5.07  0.44   0.0   6.0
## FSFIorg_NEW          8 483   4.11  2.06    4.8    4.39  1.78   0.0   6.0
## FSFIsat_NEW          9 483   4.11  1.86    4.8    4.31  1.78   0.4   6.0
## FSFIpain2_NEW       10 483   4.42  2.25    6.0    4.77  0.00   0.0   6.0
## SDS_NEW             11 483   8.63  5.95    8.0    8.22  5.93   0.0  28.0
## BSIdep_NEW          12 483   4.75  4.54    3.0    4.06  2.97   0.0  23.0
## BSIanx_NEW          13 483   3.59  4.17    2.0    2.79  2.97   0.0  24.0
## BI_NEW              14 483  26.36  6.75   26.0   26.24  7.41  12.0  44.0
## AUDIT_NEW           15 483   5.32  4.20    4.0    4.76  2.97   0.0  30.0
## DSFItoolittle_NEW   16 483   4.11  4.70    3.0    3.27  4.45   0.0  28.0
## DSFItoomuch_NEW     17 483   0.20  0.78    0.0    0.00  0.00   0.0   8.0
## SOI_NEW             18 483  74.09 39.17   63.0   66.42 19.27  24.0 261.0
## haspartner_NEW      19 483   0.85  0.36    1.0    0.93  0.00   0.0   1.0
##                   range  skew kurtosis   se
```

```
## age_NEW             26.0  0.66     0.53 0.22
## height_NEW          37.0  0.26     0.03 0.28
## weight_NEW         136.5  2.16     9.23 0.64
## biolchild_NEW        9.0  1.55     4.18 0.06
## FSFIdes_NEW          4.2  0.26    -0.23 0.04
## FSFIaro_NEW          6.0 -1.19     0.15 0.09
## FSFIlub_NEW          6.0 -1.54     0.83 0.09
## FSFIorg_NEW          6.0 -0.94    -0.51 0.09
## FSFIsat_NEW          5.6 -0.77    -0.81 0.08
## FSFIpain2_NEW        6.0 -1.20    -0.18 0.10
## SDS_NEW             28.0  0.55    -0.23 0.27
## BSIdep_NEW          23.0  1.32     1.56 0.21
## BSIanx_NEW          24.0  1.80     3.47 0.19
## BI_NEW              32.0  0.15    -0.74 0.31
## AUDIT_NEW           30.0  1.83     5.37 0.19
## DSFItoolittle_NEW  28.0  1.51     2.22 0.21
## DSFItoomuch_NEW     8.0  5.42    35.47 0.04
## SOI_NEW            237.0  2.00     3.92 1.78
## haspartner_NEW       1.0 -1.92     1.69 0.02

describe(IncreaseNEWRS)
##                    vars   n   mean    sd median trimmed   mad    min   max
## age_NEW               1 483  33.13  4.94   32.0   32.84  5.93  25.00  52.0
## height_NEW            2 483 166.42  6.27  166.5  166.38  5.39 150.00 186.0
## weight_NEW            3 483  67.74 15.00   65.0   65.68 10.38  42.00 166.0
## biolchild_NEW         4 483   1.12  1.39    1.0    0.93  1.48   0.00  12.0
## FSFIdes_NEW           5 483   3.40  1.02    3.6    3.40  0.89   1.20   5.4
## FSFIaro_NEW           6 483   4.52  1.69    5.1    4.87  0.89   0.00   6.0
## FSFIlub_NEW           7 483   5.08  1.73    6.0    5.56  0.00   0.00   6.0
## FSFIorg_NEW           8 483   4.46  1.90    5.2    4.81  1.19   0.00   6.0
## FSFIsat_NEW           9 483   4.23  1.88    4.8    4.47  1.78   0.40   6.0
## FSFIpain2_NEW        10 483   4.64  2.23    6.0    5.05  0.00   0.00   6.0
## SDS_NEW              11 483   7.90  5.95    8.0    7.40  5.93   0.00  28.0
## BSIdep_NEW           12 483   4.77  4.42    4.0    4.14  4.45   0.00  22.0
## BSIanx_NEW           13 483   3.33  3.86    2.0    2.61  2.97   0.00  22.0
## BI_NEW               14 483  25.34  7.26   25.0   25.07  7.41  11.00  47.0
## AUDIT_NEW            15 483   5.62  4.22    5.0    5.05  2.97   0.00  30.0
## DSFItoolittle_NEW    16 483   5.24  5.05    4.0    4.55  4.45   0.00  27.0
## DSFItoomuch_NEW      17 483   0.06  0.34    0.0    0.00  0.00   0.00   4.0
## SOI_NEW              18 483  78.76 44.42   65.0   70.58 23.72  15.04 252.0
## haspartner_NEW       19 483   0.77  0.42    1.0    0.84  0.00   0.00   1.0
##                     range  skew kurtosis   se
## age_NEW             27.00  0.65     0.40 0.22
## height_NEW          36.00  0.07    -0.01 0.29
## weight_NEW         124.00  1.91     6.03 0.68
## biolchild_NEW       12.00  1.82     7.66 0.06
## FSFIdes_NEW          4.20  0.09    -0.43 0.05
## FSFIaro_NEW          6.00 -1.62     1.74 0.08
## FSFIlub_NEW          6.00 -2.21     3.61 0.08
## FSFIorg_NEW          6.00 -1.24     0.30 0.09
## FSFIsat_NEW          5.60 -0.89    -0.63 0.09
## FSFIpain2_NEW        6.00 -1.42     0.32 0.10
## SDS_NEW             28.00  0.70     0.19 0.27
## BSIdep_NEW          22.00  1.18     1.04 0.20
## BSIanx_NEW          22.00  1.88     4.06 0.18
## BI_NEW              36.00  0.34    -0.29 0.33
## AUDIT_NEW           30.00  1.51     3.14 0.19
## DSFItoolittle_NEW  27.00  1.19     1.13 0.23
## DSFItoomuch_NEW     4.00  7.24    61.45 0.02
## SOI_NEW            236.96  1.71     2.40 2.02
## haspartner_NEW       1.00 -1.29    -0.33 0.02

table(DecreaseNEWRS[,19])
```

```
##
##   0   1
##  44 439
table(DecreaseNEWRS[,19])[1]/483
##          0
## 0.09109731
table(DecreaseNEWRS[,19])[2]/483
##         1
## 0.9089027

table(StableNEWRS[,19])
##
##   0   1
##  74 409
table(StableNEWRS[,19])[1]/483
##         0
## 0.1532091
table(StableNEWRS[,19])[2]/483
##         1
## 0.8467909

table(IncreaseNEWRS[,19])
##
##   0   1
## 110 373
table(IncreaseNEWRS[,19])[1]/483
##         0
## 0.2277433
table(IncreaseNEWRS[,19])[2]/483
##         1
## 0.7722567


VarDomain <- c("g","g","g",
               "p","g","g",
               "g","g","g",
               "g","g","g",
               "g","g","g",
               "g","g","g",
               "c")
VarLevel <- c(1,1,1,
              1,1,1,
              1,1,1,
              1,1,1,
              1,1,1,
              1,1,1,
              2)

fit_DecreaseNEW <- mgm(data = as.matrix(DecreaseNEWRS),
                       type = VarDomain,
                       level = VarLevel,
                       k = 2,
                       lambdaSel = 'EBIC',
                       lambdaGam = 0.5) # hyper parameter

fit_StableNEW <- mgm(data = as.matrix(StableNEWRS),
                     type = VarDomain,
                     level = VarLevel,
                     k = 2,
                     lambdaSel = 'EBIC',
                     lambdaGam = 0.5) # hyper parameter
```

```
fit_IncreaseNEW <- mgm(data = as.matrix(IncreaseNEWRS),
                       type = VarDomain,
                       level = VarLevel,
                       k = 2,
                       lambdaSel = 'EBIC',
                       lambdaGam = 0.5) # hyper parameter

########################
# Predictive Strength ##
########################

pred_DecreaseNEW <- predict(fit_DecreaseNEW,
                        DecreaseNEWRS,
                        errorCon = 'R2') #save R2 into an object (varExpl = cont var)

pred_StableNEW <- predict(fit_StableNEW,
                       StableNEWRS,
                       errorCon = 'R2') #save R2 into an object (varExpl = cont var)

pred_IncreaseNEW <- predict(fit_IncreaseNEW,
                        IncreaseNEWRS,
                        errorCon = 'R2') #save R2 into an object (varExpl = cont var)

predR_DecreaseNEW <- pred_DecreaseNEW$errors$Error.R2
predR_DecreaseNEW <- as.numeric(as.character(predR_DecreaseNEW))

predR_StableNEW <- pred_StableNEW$errors$Error.R2
predR_StableNEW <- as.numeric(as.character(predR_StableNEW))

predR_IncreaseNEW <- pred_IncreaseNEW$errors$Error.R2
predR_IncreaseNEW <- as.numeric(as.character(predR_IncreaseNEW))

# set 'predictability# of categorical variable

predR_DecreaseNEW[19] <- pred_DecreaseNEW$errors$Error.CC[19]
predR_StableNEW[19] <- pred_StableNEW$errors$Error.CC[19]
predR_IncreaseNEW[19] <- pred_IncreaseNEW$errors$Error.CC[19]

mean(predR_DecreaseNEW, na.rm = TRUE) #mean variance explained across all nodes
## [1] 0.4255789
sd(predR_DecreaseNEW, na.rm = TRUE)
## [1] 0.3061924

pred_DecreaseNEW$errors
##              Variable Error.R2 Error.CC Error.nCC CCmarg
## 1             age_NEW    0.140       NA        NA     NA
## 2          height_NEW    0.056       NA        NA     NA
## 3          weight_NEW    0.125       NA        NA     NA
## 4       biolchild_NEW    0.175       NA        NA     NA
## 5         FSFIdes_NEW    0.391       NA        NA     NA
## 6         FSFIaro_NEW    0.894       NA        NA     NA
## 7         FSFIlub_NEW    0.816       NA        NA     NA
## 8         FSFIorg_NEW    0.747       NA        NA     NA
## 9         FSFIsat_NEW    0.804       NA        NA     NA
## 10      FSFIpain2_NEW    0.603       NA        NA     NA
## 11            SDS_NEW    0.417       NA        NA     NA
## 12         BSIdep_NEW    0.576       NA        NA     NA
## 13         BSIanx_NEW    0.539       NA        NA     NA
## 14             BI_NEW    0.212       NA        NA     NA
## 15          AUDIT_NEW    0.146       NA        NA     NA
## 16 DSFItoolittle_NEW    0.366       NA        NA     NA
## 17   DSFItoomuch_NEW    0.101       NA        NA     NA
```

```
## 18           SOI_NEW    0.048       NA         NA      NA
## 19    haspartner_NEW       NA     0.93      0.227   0.909

mean(predR_StableNEW, na.rm = TRUE) #mean variance explained across all nodes
## [1] 0.4344211
sd(predR_StableNEW, na.rm = TRUE)
## [1] 0.2920332

pred_StableNEW$errors
##              Variable Error.R2 Error.CC Error.nCC CCmarg
## 1             age_NEW    0.204       NA         NA      NA
## 2          height_NEW    0.136       NA         NA      NA
## 3          weight_NEW    0.258       NA         NA      NA
## 4       biolchild_NEW    0.274       NA         NA      NA
## 5         FSFIdes_NEW    0.188       NA         NA      NA
## 6         FSFIaro_NEW    0.883       NA         NA      NA
## 7         FSFIlub_NEW    0.856       NA         NA      NA
## 8         FSFIorg_NEW    0.726       NA         NA      NA
## 9         FSFIsat_NEW    0.768       NA         NA      NA
## 10      FSFIpain2_NEW    0.655       NA         NA      NA
## 11            SDS_NEW    0.313       NA         NA      NA
## 12          BSIdep_NEW    0.591       NA         NA      NA
## 13          BSIanx_NEW    0.519       NA         NA      NA
## 14             BI_NEW    0.264       NA         NA      NA
## 15          AUDIT_NEW    0.203       NA         NA      NA
## 16 DSFItoolittle_NEW    0.474       NA         NA      NA
## 17   DSFItoomuch_NEW    0.021       NA         NA      NA
## 18            SOI_NEW    0.043       NA         NA      NA
## 19     haspartner_NEW       NA    0.878      0.203   0.847

mean(predR_IncreaseNEW, na.rm = TRUE) #mean variance explained across all nodes
## [1] 0.4360526
sd(predR_IncreaseNEW, na.rm = TRUE)
## [1] 0.2661239

pred_IncreaseNEW$errors
##              Variable Error.R2 Error.CC Error.nCC CCmarg
## 1             age_NEW    0.170       NA         NA      NA
## 2          height_NEW    0.142       NA         NA      NA
## 3          weight_NEW    0.235       NA         NA      NA
## 4       biolchild_NEW    0.250       NA         NA      NA
## 5         FSFIdes_NEW    0.250       NA         NA      NA
## 6         FSFIaro_NEW    0.877       NA         NA      NA
## 7         FSFIlub_NEW    0.836       NA         NA      NA
## 8         FSFIorg_NEW    0.632       NA         NA      NA
## 9         FSFIsat_NEW    0.695       NA         NA      NA
## 10      FSFIpain2_NEW    0.608       NA         NA      NA
## 11            SDS_NEW    0.374       NA         NA      NA
## 12          BSIdep_NEW    0.594       NA         NA      NA
## 13          BSIanx_NEW    0.515       NA         NA      NA
## 14             BI_NEW    0.336       NA         NA      NA
## 15          AUDIT_NEW    0.227       NA         NA      NA
## 16 DSFItoolittle_NEW    0.558       NA         NA      NA
## 17   DSFItoomuch_NEW    0.023       NA         NA      NA
## 18            SOI_NEW    0.135       NA         NA      NA
## 19     haspartner_NEW       NA    0.828      0.245   0.772
```

14. Replication – Network Visualization

```
############################
# Network Layout Specifics #
############################
```

```
colNames <- c("Age", "Height", "Weight", "Child", "Des", "Aro",
              "Lub", "Org", "Sat", "Pain", "Distr", "Dep", "Anx",
              "Body", "Alc", "Little", "Much", "SO", "Rel")

nodeDescr <- c("", "", "", "Number of children", "Desire",
               "Arousal","Lubrication", "Orgasm", "Satisfaction", "Pain",
               "Sexually-related personal distress", "Depression", "Anxiety",
               "Body image and dissatisfaction", "(Hazardous) Alcohol use",
               "Too little sexual sctivity", "Too much sexual activity",
               "Tendency/attitudes to(wards) uncommitted sexual relationships",
               "Relationship status (partnered or single)")

groupsNoHc <- list(Demographics = c(1:4,19),
                   Female_Sexual_Function_Index = c(5:10),
                   Sexual_Distress_Scale = c(11),
                   Brief_Symptom_Inventory = c(12:13),
                   Derogatis_Sexual_Function_Inventory = c(14),
                   Alcohol_Use_Disorders_Identification_Test = c(15),
                   Desired_and_Actual_Sexual_Activity_Scale = c(16:17),
                   Sociosexual_Orientation_Inventory = c(18))

nodeLabelColors <- c("white", "white", "white", "white",
                     "white","white","white","white","white","white",
                     "white",
                     "white","white",
                     "black","black","black","black","black",
                     "white")

#Plot Networks
library(qgraph)

fit_DecreaseNEW$pairwise$edgecolor[which(fit_DecreaseNEW$pairwise$edgecolor=="red")]<- "gray50"
fit_DecreaseNEW$pairwise$edgecolor[which(fit_DecreaseNEW$pairwise$edgecolor=="darkgreen")] <- "
gray70"
fit_DecreaseNEW$pairwise$edgecolor[which(fit_DecreaseNEW$pairwise$edgecolor=="darkgrey")] <- "b
lack"
fit_DecreaseNEW$pairwise$edgecolor[c(16),19] <- "gray50" # categ relationship
fit_DecreaseNEW$pairwise$edgecolor[c(19),16] <- "gray50"
fit_DecreaseNEW$pairwise$edgecolor[c(4,11),19] <- "gray70"
fit_DecreaseNEW$pairwise$edgecolor[19,c(4,11)] <- "gray70"
fit_DecreaseNEW$pairwise$wadj[which(fit_DecreaseNEW$pairwise$edgecolor=="gray50")] <- fit_Decre
aseNEW$pairwise$wadj[which(fit_DecreaseNEW$pairwise$edgecolor=="gray50")]*-1

fit_DecreaseNEW$pairwise$wadj[which(fit_DecreaseNEW$pairwise$edgecolor=="gray50")]
##  [1] -0.0734519 -0.1072308 -0.1290201 -0.2589329 -0.2615573 -0.1290201
##  [7] -0.2589329 -0.0734519 -0.1072308 -0.2615573 -0.1343503 -0.5925076
## [13] -0.1343503 -0.5925076

qDecreaseNEW <- qgraph(fit_DecreaseNEW$pairwise$wadj,
                    edge.color = fit_DecreaseNEW$pairwise$edgecolor,
                    layout = LayoutAverage,
                    labels = colNames,
                    maximum = 1,
                    minimum = 0,
                    cut = 0.1,
                    details = TRUE,
                    vsize = 7.5,
                    esize = 10,
                    label.cex = 1.2,
                    label.scale.equal = TRUE,
                    pie = predR_DecreaseNEW,
                    pieColor = "gray21",
```

```r
                     pieBorder = 0.25,
                     groups = groupsNoHc,
                     nodeNames = nodeDescr,
                     legend = TRUE,
                     legend.mode = "style1",
                     legend.cex = 0.5,
                     palette = "gray",
                     negDashed = TRUE,
                     label.color = nodeLabelColors)
```

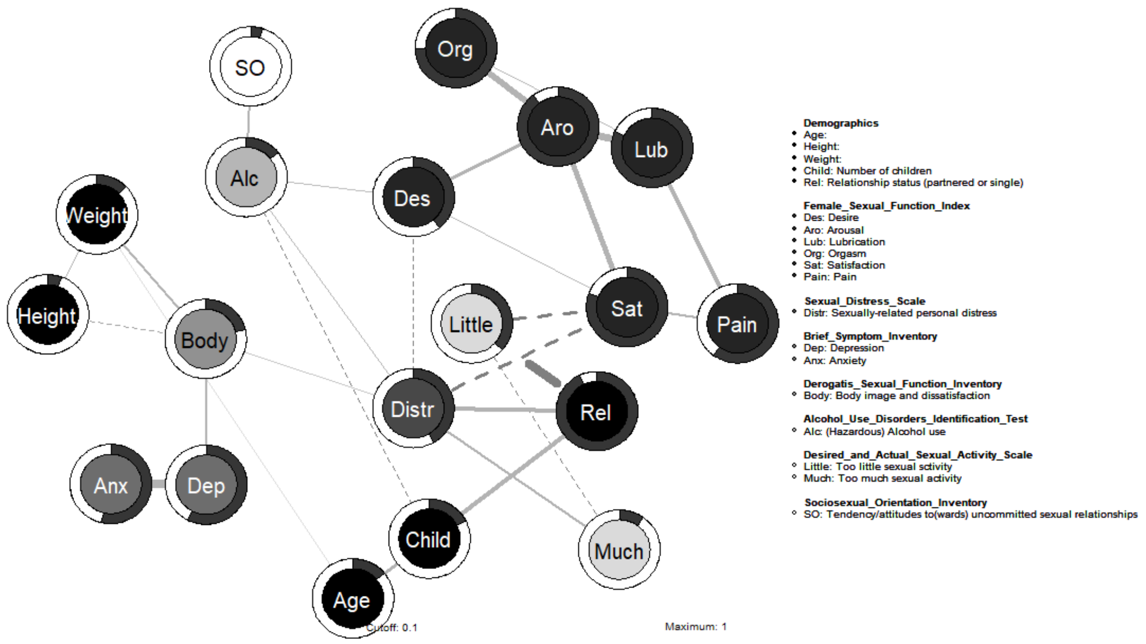


```r
fit_StableNEW$pairwise$edgecolor[which(fit_StableNEW$pairwise$edgecolor=="red")] <- "gray50"
fit_StableNEW$pairwise$edgecolor[which(fit_StableNEW$pairwise$edgecolor=="darkgreen")] <- "gray
70"
fit_StableNEW$pairwise$edgecolor[which(fit_StableNEW$pairwise$edgecolor=="darkgrey")] <- "black
"
fit_StableNEW$pairwise$edgecolor[c(16),19] <- "gray50" # categ relationship
fit_StableNEW$pairwise$edgecolor[c(19),16] <- "gray50"
fit_StableNEW$pairwise$edgecolor[c(4,10),19] <- "gray70"
fit_StableNEW$pairwise$edgecolor[19,c(4,10)] <- "gray70"
fit_StableNEW$pairwise$wadj[which(fit_StableNEW$pairwise$edgecolor=="gray50")] <- fit_StableNEW
$pairwise$wadj[which(fit_StableNEW$pairwise$edgecolor=="gray50")]*-1

fit_StableNEW$pairwise$wadj[which(fit_StableNEW$pairwise$edgecolor=="gray50")]
##  [1] -0.12336030 -0.17160096 -0.18511236 -0.24273522 -0.18511236
##  [6] -0.12336030 -0.17160096 -0.24273522 -0.09988514 -0.73386154
## [11] -0.09988514 -0.73386154

qStableNEW <- qgraph(fit_StableNEW$pairwise$wadj,
                  edge.color = fit_StableNEW$pairwise$edgecolor,
                  layout = LayoutAverage,
                  labels = colNames,
                  maximum = 1,
                  minimum = 0,
                  cut = 0.1,
                  details = TRUE,
                  vsize = 7.5,
                  esize = 10,
                  label.cex = 1.2,
```

```
                  label.scale.equal = TRUE,
                  pie = predR_StableNEW,
                  pieColor = "gray21",
                  pieBorder = 0.25,
                  groups = groupsNoHc,
                  nodeNames = nodeDescr,
                  legend = TRUE,
                  legend.mode = "style1",
                  legend.cex = 0.5,
                  palette = "gray",
                  negDashed = TRUE,
                  label.color = nodeLabelColors)
```

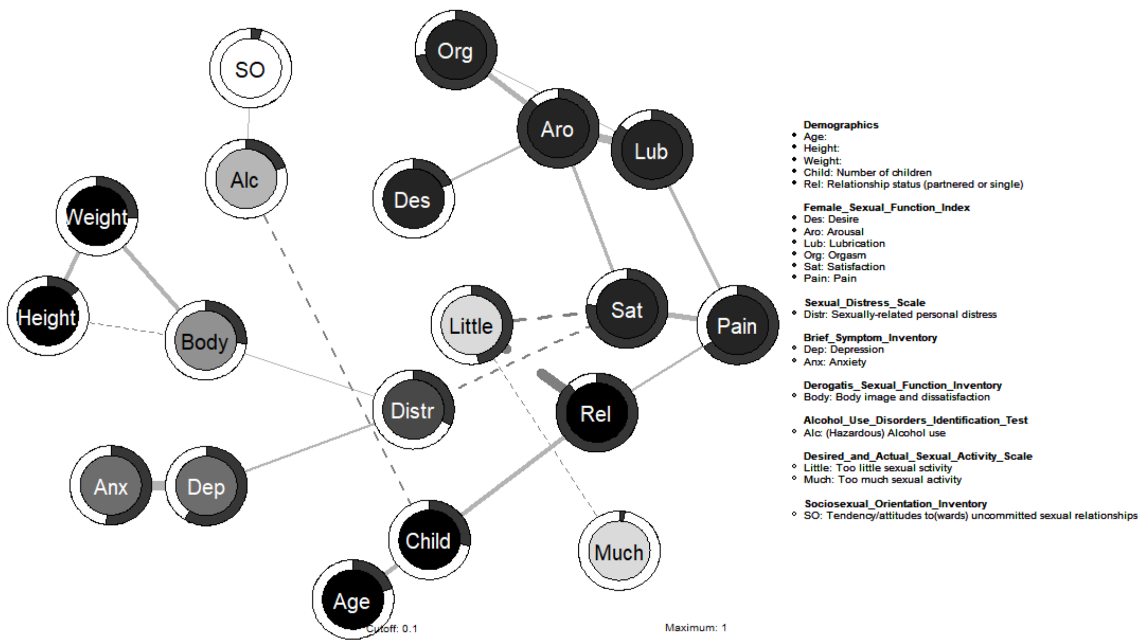


```
fit_IncreaseNEW$pairwise$edgecolor[which(fit_IncreaseNEW$pairwise$edgecolor=="red")] <- "gray50
"
fit_IncreaseNEW$pairwise$edgecolor[which(fit_IncreaseNEW$pairwise$edgecolor=="darkgreen")] <- "
gray70"
fit_IncreaseNEW$pairwise$edgecolor[which(fit_IncreaseNEW$pairwise$edgecolor=="darkgrey")] <- "b
lack"
fit_IncreaseNEW$pairwise$edgecolor[c(16),19] <- "gray50" # categ relationship
fit_IncreaseNEW$pairwise$edgecolor[c(19),16] <- "gray50"
fit_IncreaseNEW$pairwise$edgecolor[c(10),19] <- "gray70"
fit_IncreaseNEW$pairwise$edgecolor[19,c(10)] <- "gray70"
fit_IncreaseNEW$pairwise$wadj[which(fit_IncreaseNEW$pairwise$edgecolor=="gray50")] <- fit_Incre
aseNEW$pairwise$wadj[which(fit_IncreaseNEW$pairwise$edgecolor=="gray50")]*-1

fit_IncreaseNEW$pairwise$wadj[which(fit_IncreaseNEW$pairwise$edgecolor=="gray50")]
##  [1] -0.12932642 -0.07260171 -0.13256000 -0.24693582 -0.13256000
##  [6] -0.12932642 -0.07260171 -0.24693582 -0.09528064 -0.68644211
## [11] -0.09528064 -0.68644211

qIncreaseNEW <- qgraph(fit_IncreaseNEW$pairwise$wadj,
                    edge.color = fit_IncreaseNEW$pairwise$edgecolor,
                    layout = LayoutAverage,
                    labels = colNames,
                    maximum = 1,
```

```
                    minimum = 0,
                    cut = 0.1,
                    details = TRUE,
                    vsize = 7.5,
                    esize = 10,
                    label.cex = 1.2,
                    label.scale.equal = TRUE,
                    pie = predR_IncreaseNEW,
                    pieColor = "gray21",
                    pieBorder = 0.25,
                    groups = groupsNoHc,
                    nodeNames = nodeDescr,
                    legend = TRUE,
                    legend.mode = "style1",
                    legend.cex = 0.5,
                    palette = "gray",
                    negDashed = TRUE,
                    label.color = nodeLabelColors)
```

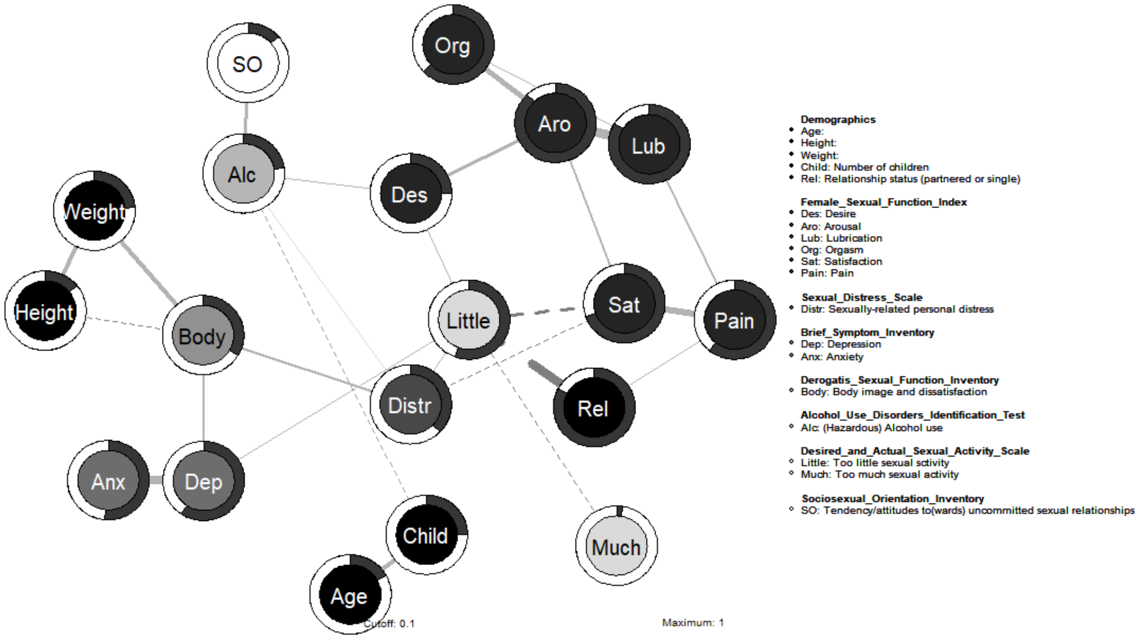


```
pdf('FSFINetworkWithPrednoHCGrayScaleReplication.pdf', width = 55, height = 15)
par(mfrow = c(1,3))
qDecreaseNEW <- qgraph(fit_DecreaseNEW$pairwise$wadj,
                       edge.color = fit_DecreaseNEW$pairwise$edgecolor,
                       layout = LayoutAverage,
                       labels = colNames,
                       maximum = 1,
                       minimum = 0,
                       cut = 0.1,
                       details = TRUE,
                       vsize = 10,
                       label.cex = 1.2,
                       label.scale.equal = TRUE,
                       pie = predR_DecreaseNEW,
                       pieColor = "gray21",
                       pieBorder = 0.25,
                       groups = groupsNoHc,
                       nodeNames = nodeDescr,
                       legend = TRUE,
```

```
                       legend.mode = "style1",
                       legend.cex = 0.5,
                       palette = "gray",
                       negDashed = TRUE,
                       label.color = nodeLabelColors,
                       repulsion = 2)

qStableNEW <- qgraph(fit_StableNEW$pairwise$wadj,
                     edge.color = fit_StableNEW$pairwise$edgecolor,
                     layout = LayoutAverage,
                     labels = colNames,
                     maximum = 1,
                     minimum = 0,
                     cut = 0.1,
                     details = TRUE,
                     vsize = 10,
                     label.cex = 1.2,
                     label.scale.equal = TRUE,
                     pie = predR_StableNEW,
                     pieColor = "gray21",
                     pieBorder = 0.25,
                     groups = groupsNoHc,
                     nodeNames = nodeDescr,
                     legend = TRUE,
                     legend.mode = "style1",
                     legend.cex = 0.5,
                     palette = "gray",
                     negDashed = TRUE,
                     label.color = nodeLabelColors,
                     repulsion = 2)

qIncreaseNEW <- qgraph(fit_IncreaseNEW$pairwise$wadj,
                       edge.color = fit_IncreaseNEW$pairwise$edgecolor,
                       layout = LayoutAverage,
                       labels = colNames,
                       maximum = 1,
                       minimum = 0,
                       cut = 0.1,
                       details = TRUE,
                       vsize = 10,
                       label.cex = 1.2,
                       label.scale.equal = TRUE,
                       pie = predR_IncreaseNEW,
                       pieColor = "gray21",
                       pieBorder = 0.25,
                       groups = groupsNoHc,
                       nodeNames = nodeDescr,
                       legend = TRUE,
                       legend.mode = "style1",
                       legend.cex = 0.5,
                       palette = "gray",
                       negDashed = TRUE,
                       label.color = nodeLabelColors,
                       repulsion = 2)
dev.off()
## png
##   2
```

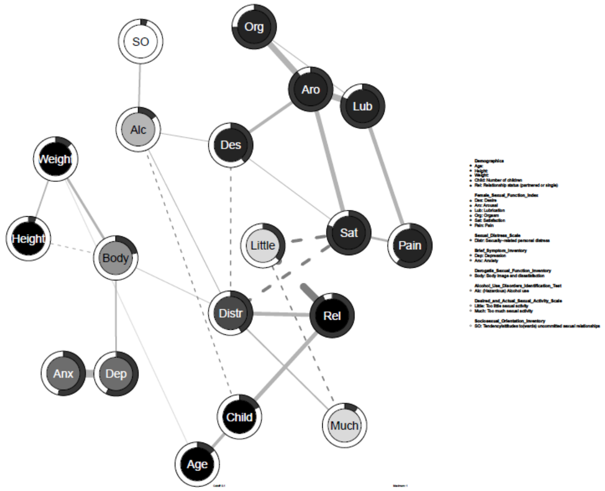

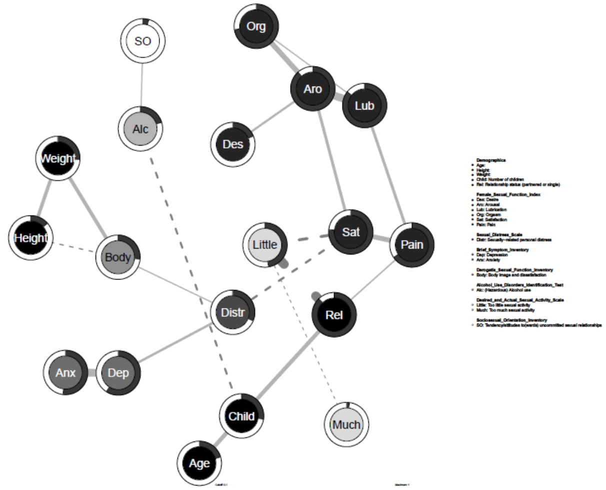

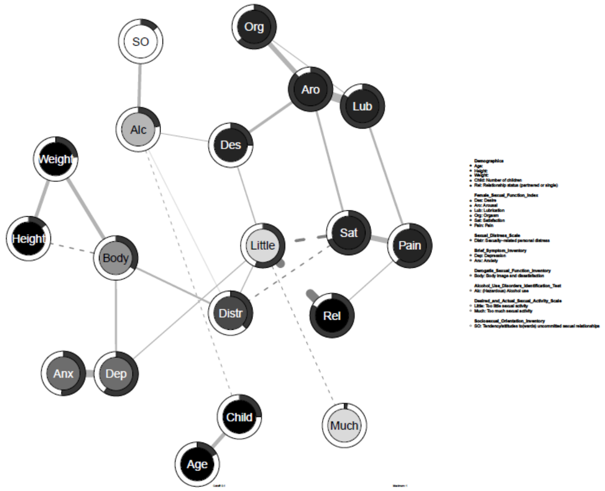


```
# For comparison's sake we copied the first graph here below:
```

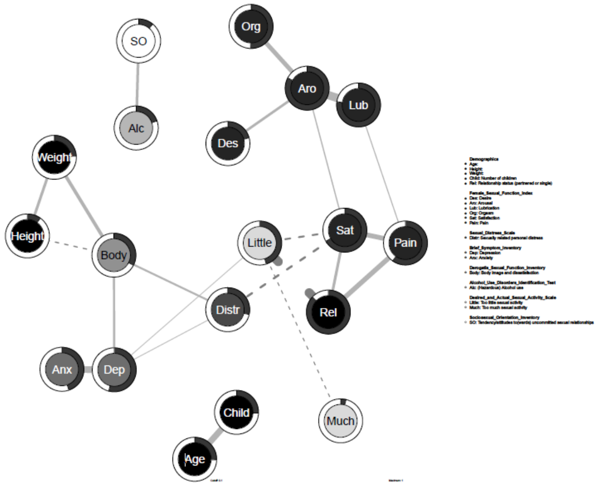

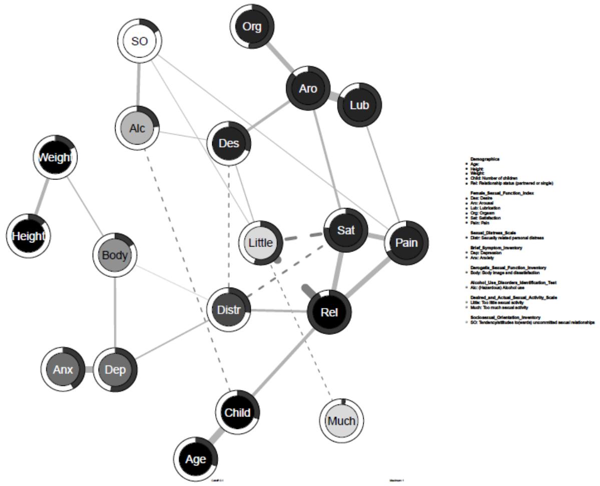

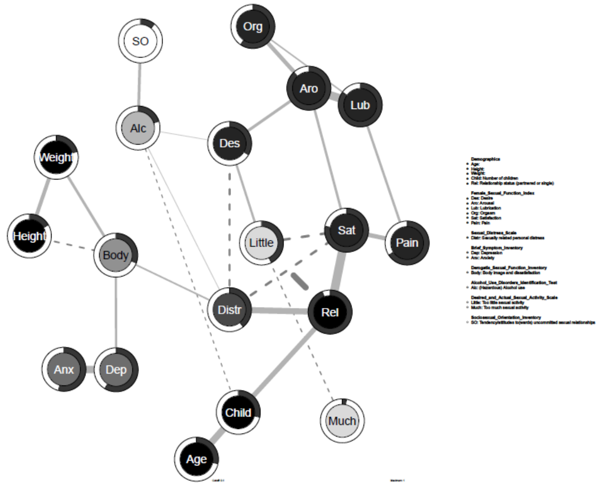

```r
length(qDecreaseNEW$Edgelist$weight)
## [1] 28
length(qStableNEW$Edgelist$weight)
## [1] 22
length(qIncreaseNEW$Edgelist$weight)
## [1] 26

# CUSTOM MADE CENTRALITY PLOT
cenDecreaseNEW <- centralityTable(qDecreaseNEW, standardized = FALSE, relative = TRUE)
cenIncreaseNEW <- centralityTable(qIncreaseNEW, standardized = FALSE, relative = TRUE)
cenStableNEW <- centralityTable(qStableNEW, standardized = FALSE, relative = TRUE)
pdf("CustomCentralityFSFINetworkReplication.pdf", width = 15, height = 7)
plot(cenDecreaseNEW$value[39:57],
     type = "b",
     bty = "l",
     family = "sans",
     las = 1,
     lwd = 1,
     xaxt="n",
     xlab = "Node",
     ylab = "Strength Centrality")
axis(side = 1, labels = colNames, at = c(1:19))
lines(cenIncreaseNEW$value[39:57], type = "b", lty = "dotted")
lines(cenStableNEW$value[39:57], type = "b", lty = "dashed")
legend(x = 1, legend=c("Decrease", "Stable", "Increase"),
       lty=c("solid", "dashed", "dotted"))
dev.off()
```

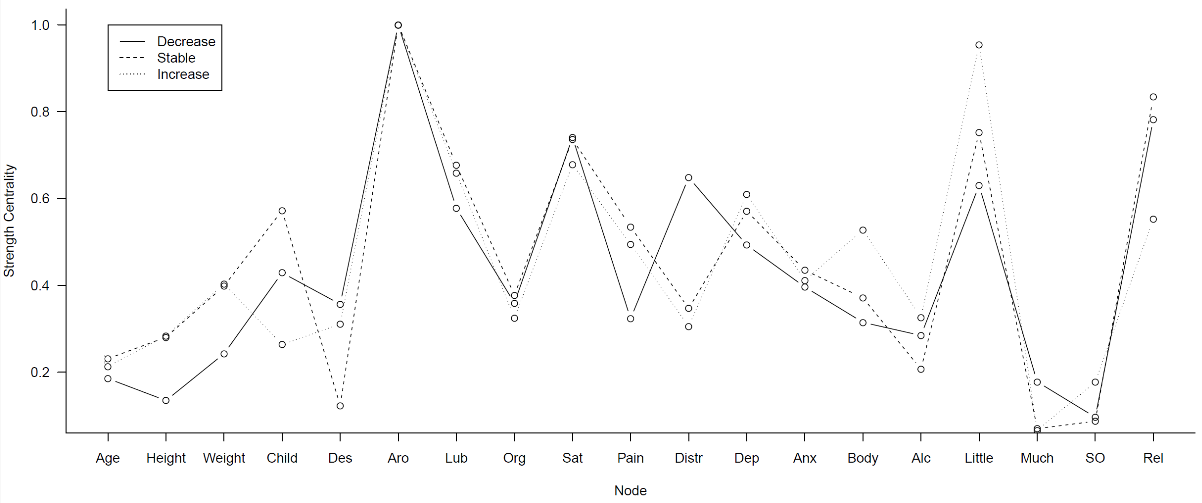


```r
# For comparison's sake we copied the first graph here below:
```

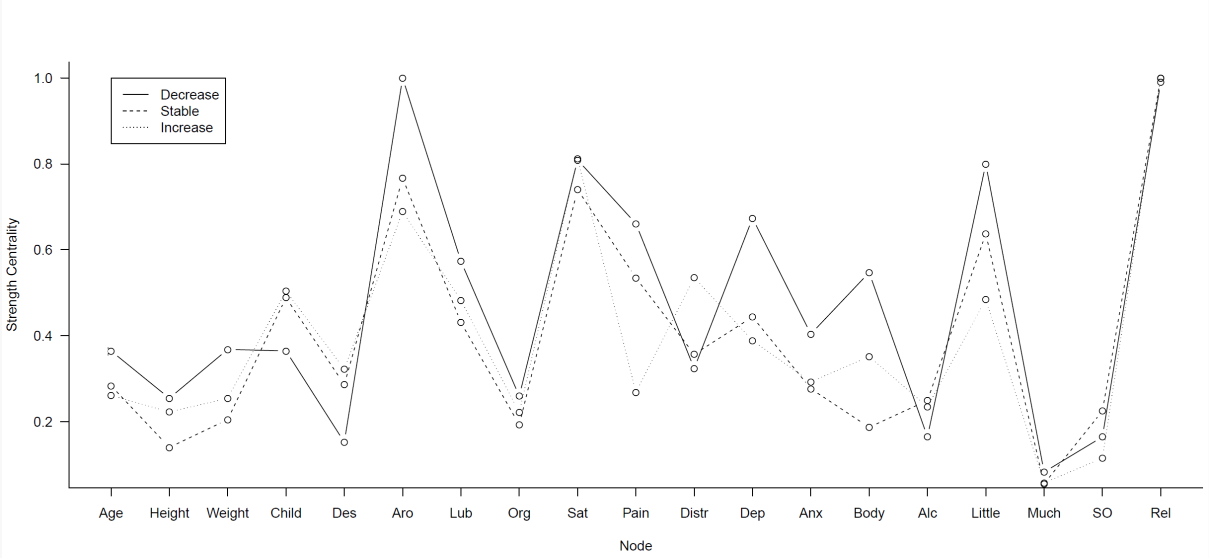


```
########################################################################
# EDGE COEFFICIENTS

fit_DecreaseNEW$rawfactor$indicator[[1]][7,] # 4 child
## [1]  4 19
fit_DecreaseNEW$rawfactor$indicator[[1]][23,] # 11 distress
## [1] 11 19
fit_DecreaseNEW$rawfactor$indicator[[1]][28,] # 16 too little
## [1] 16 19
fit_DecreaseNEW$rawfactor$weights[[1]][7]
## [[1]]
## [[1]][[1]]
##             [,1]
## V19.1 0.4850364
##
## [[1]][[2]]
## [1] -0.1563666  0.1563666
fit_DecreaseNEW$rawfactor$weights[[1]][23]
## [[1]]
## [[1]][[1]]
##             [,1]
## V19.1 0.5823611
##
## [[1]][[2]]
## [1] -0.04556507  0.04556507
fit_DecreaseNEW$rawfactor$weights[[1]][28]
## [[1]]
## [[1]][[1]]
##             [,1]
## V19.1 -0.793869
##
## [[1]][[2]]
## [1]  0.3911461 -0.3911461
fit_StableNEW$rawfactor$indicator[[1]][6,] # 4 child
## [1]  4 19
fit_StableNEW$rawfactor$indicator[[1]][16,] # 10 pain
## [1] 10 19
fit_StableNEW$rawfactor$indicator[[1]][22,] # 16 too little
## [1] 16 19
fit_StableNEW$rawfactor$weights[[1]][6]
## [[1]]
## [[1]][[1]]
##             [,1]
```

```
## V19.1 0.5694836
##
## [[1]][[2]]
## [1] -0.06555586  0.06555586
fit_StableNEW$rawfactor$weights[[1]][16]
## [[1]]
## [[1]][[1]]
##           [,1]
## V19.1 0.160404
##
## [[1]][[2]]
## [1] -0.1257695  0.1257695
fit_StableNEW$rawfactor$weights[[1]][22]
## [[1]]
## [[1]][[1]]
##           [,1]
## V19.1 -1.03362
##
## [[1]][[2]]
## [1]  0.4341031 -0.4341031

fit_IncreaseNEW$rawfactor$indicator[[1]][17,] # 10 pain
## [1] 10 19
fit_IncreaseNEW$rawfactor$indicator[[1]][26,] # 16 too little
## [1] 16 19
fit_IncreaseNEW$rawfactor$weights[[1]][17]
## [[1]]
## [[1]][[1]]
##            [,1]
## V19.1 0.1343324
##
## [[1]][[2]]
## [1] -0.08722788  0.08722788
fit_IncreaseNEW$rawfactor$weights[[1]][26]
## [[1]]
## [[1]][[1]]
##             [,1]
## V19.1 -0.8716168
##
## [[1]][[2]]
## [1]  0.5012674 -0.5012674
```

15. Replication – Network Stability

```
#############################################################
# BOOTNET
library(bootnet)

fit_DecreaseBootNEW <- estimateNetwork(as.matrix(DecreaseNEWRS),
                                    default = "mgm",
                                    type = VarDomain,
                                    lev = VarLevel,
                                    degree = 2,
                                    criterion = "EBIC",
                                    tuning = 0.5)

fit_StableBootNEW <- estimateNetwork(as.matrix(StableNEWRS),
                                  default = "mgm",
                                  type = VarDomain,
                                  lev = VarLevel,
                                  degree = 2,
```

```
                                   criterion = "EBIC",
                                   tuning = 0.5)

fit_IncreaseBootNEW <- estimateNetwork(as.matrix(IncreaseNEWRS),
                                     default = "mgm",
                                     type = VarDomain,
                                     lev = VarLevel,
                                     degree = 2,
                                     criterion = "EBIC",
                                     tuning = 0.5)

fit_DecreaseBootNEW1 <- bootnet(fit_DecreaseBootNEW, nBoots = 1000, nCores = 8,type = "case")

fit_StableBootNEW1 <- bootnet(fit_StableBootNEW, nBoots = 1000, nCores = 8,type = "case")

fit_IncreaseBootNEW1 <- bootnet(fit_IncreaseBootNEW, nBoots = 1000, nCores = 8,type = "case")

pdf("CentralityStabilityReplication.pdf")
plot(fit_DecreaseBootNEW1)
## Warning in plot.bootnet(fit_DecreaseBootNEW1): Statistic closeness does not
## contain any variance and is therefore not shown.
plot(fit_StableBootNEW1)
## Warning in plot.bootnet(fit_StableBootNEW1): Statistic closeness does not
## contain any variance and is therefore not shown.
plot(fit_IncreaseBootNEW1)
## Warning in plot.bootnet(fit_IncreaseBootNEW1): Statistic closeness does not
## contain any variance and is therefore not shown.
dev.off()
## png
##   2
```

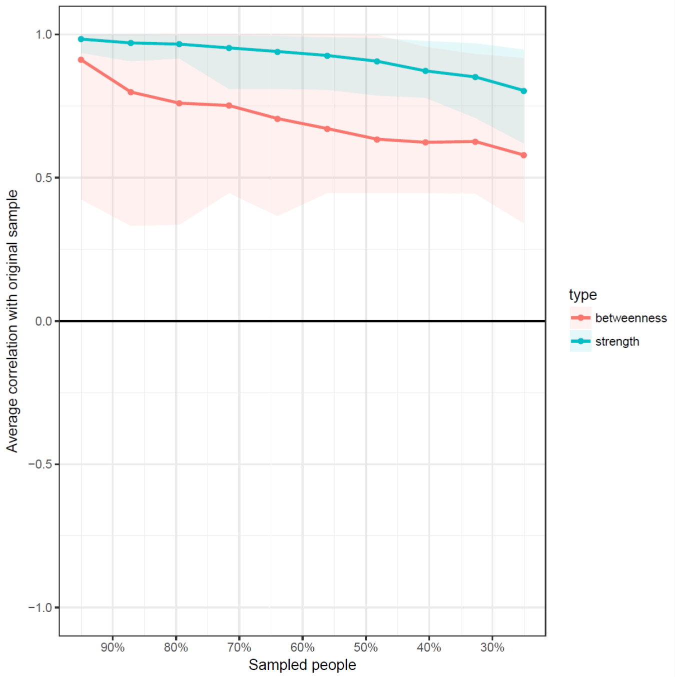

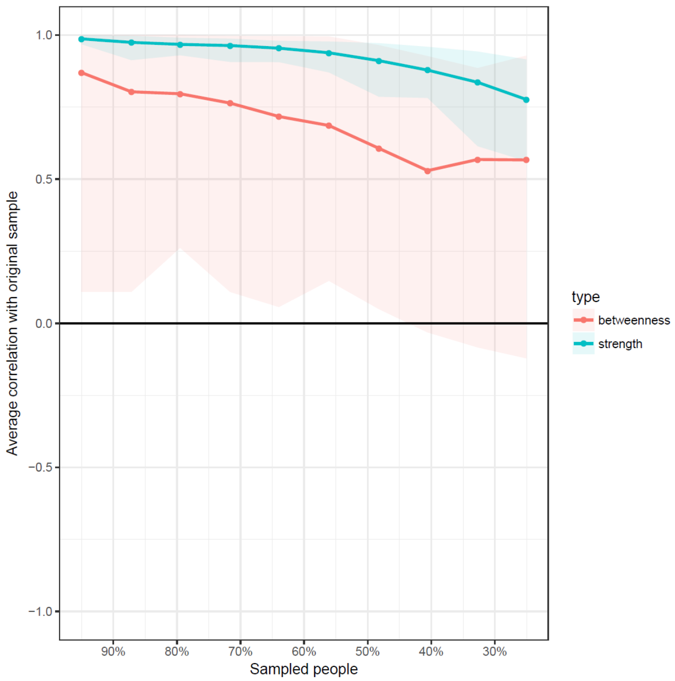

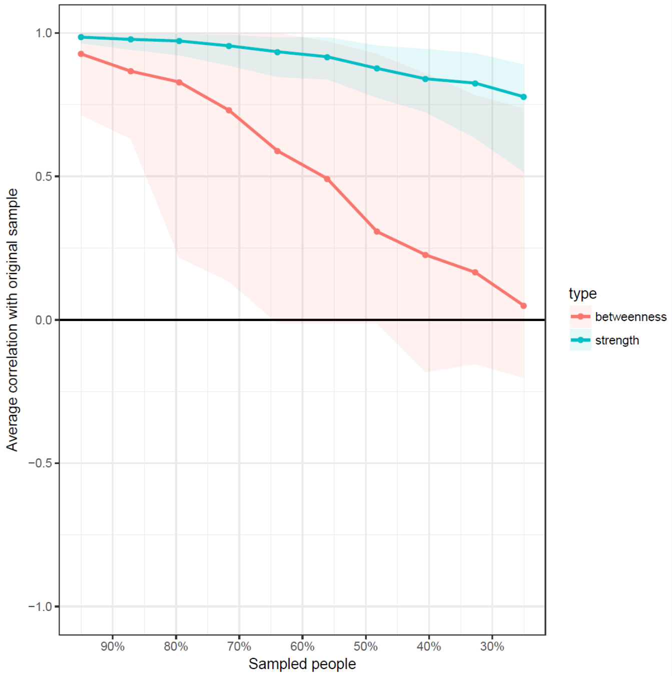


```
corStability(fit_DecreaseBootNEW1)
## === Correlation Stability Analysis ===
##
## Sampling levels tested:
##    nPerson Drop%   n
## 1      121  74.9  93
## 2      158  67.3 119
## 3      196  59.4 109
## 4      233  51.8  99
## 5      271  43.9 100
## 6      309  36.0  90
## 7      346  28.4 112
## 8      384  20.5  85
```

```
## 9      421  12.8  93
## 10     459   5.0 100
##
## Maximum drop proportions to retain correlation of 0.7 in at least 95% of the samples:
##
## betweenness: 0
##   - For more accuracy, run bootnet(..., caseMin = 0, caseMax = 0.05)
##
## closeness: 0
##   - For more accuracy, run bootnet(..., caseMin = 0, caseMax = 0.05)
##
## strength: 0.673
##   - For more accuracy, run bootnet(..., caseMin = 0.594, caseMax = 0.749)
##
## Accuracy can also be increased by increasing both 'nBoots' and 'caseN'.
```

```
corStability(fit_StableBootNEW1)
## === Correlation Stability Analysis ===
##
## Sampling levels tested:
##    nPerson Drop%   n
## 1      121  74.9  95
## 2      158  67.3 113
## 3      196  59.4  96
## 4      233  51.8 106
## 5      271  43.9 107
## 6      309  36.0  91
## 7      346  28.4  88
## 8      384  20.5 101
## 9      421  12.8 113
## 10     459   5.0  90
##
## Maximum drop proportions to retain correlation of 0.7 in at least 95% of the samples:
##
## betweenness: 0
##   - For more accuracy, run bootnet(..., caseMin = 0, caseMax = 0.05)
##
## closeness: 0
##   - For more accuracy, run bootnet(..., caseMin = 0, caseMax = 0.05)
##
## strength: 0.594
##   - For more accuracy, run bootnet(..., caseMin = 0.518, caseMax = 0.673)
##
## Accuracy can also be increased by increasing both 'nBoots' and 'caseN'.
```

```
corStability(fit_IncreaseBootNEW1)
## === Correlation Stability Analysis ===
##
## Sampling levels tested:
##    nPerson Drop%   n
## 1      121  74.9  96
## 2      158  67.3  94
## 3      196  59.4 105
## 4      233  51.8  87
## 5      271  43.9 108
## 6      309  36.0 111
## 7      346  28.4 103
## 8      384  20.5 108
## 9      421  12.8  92
## 10     459   5.0  96
##
## Maximum drop proportions to retain correlation of 0.7 in at least 95% of the samples:
##
```

```
## betweenness: 0.128
##   - For more accuracy, run bootnet(..., caseMin = 0.05, caseMax = 0.205)
##
## closeness: 0
##   - For more accuracy, run bootnet(..., caseMin = 0, caseMax = 0.05)
##
## strength: 0.673
##   - For more accuracy, run bootnet(..., caseMin = 0.594, caseMax = 0.749)
##
## Accuracy can also be increased by increasing both 'nBoots' and 'caseN'.
fit_DecreaseBootNEW2 <- bootnet(fit_DecreaseBootNEW, nBoots = 1000, nCores = 8)

fit_StableBootNEW2 <- bootnet(fit_StableBootNEW, nBoots = 1000, nCores = 8)

fit_IncreaseBootNEW2 <- bootnet(fit_IncreaseBootNEW, nBoots = 1000, nCores = 8)

# See pdf for edge stability plots
pdf("EdgeStabilityReplication.pdf", height = 25)
plot(fit_DecreaseBootNEW2, labels = TRUE, order = "sample")
plot(fit_StableBootNEW2, labels = TRUE, order = "sample")
plot(fit_IncreaseBootNEW2, labels = TRUE, order = "sample")
dev.off()
## png
##   2

pdf("EdgeSignificanceReplication.pdf")
plot(fit_DecreaseBootNEW2, "edge",plot = "difference",onlyNonZero = TRUE,
     order = "sample")
## Expected significance level given number of bootstrap samples is approximately: 0.05
plot(fit_StableBootNEW2, "edge", plot = "difference",onlyNonZero = TRUE,
     order = "sample")
## Expected significance level given number of bootstrap samples is approximately: 0.05
plot(fit_IncreaseBootNEW2, "edge", plot = "difference",onlyNonZero = TRUE,
     order = "sample")
## Expected significance level given number of bootstrap samples is approximately: 0.05
dev.off()
## png
##   2
```

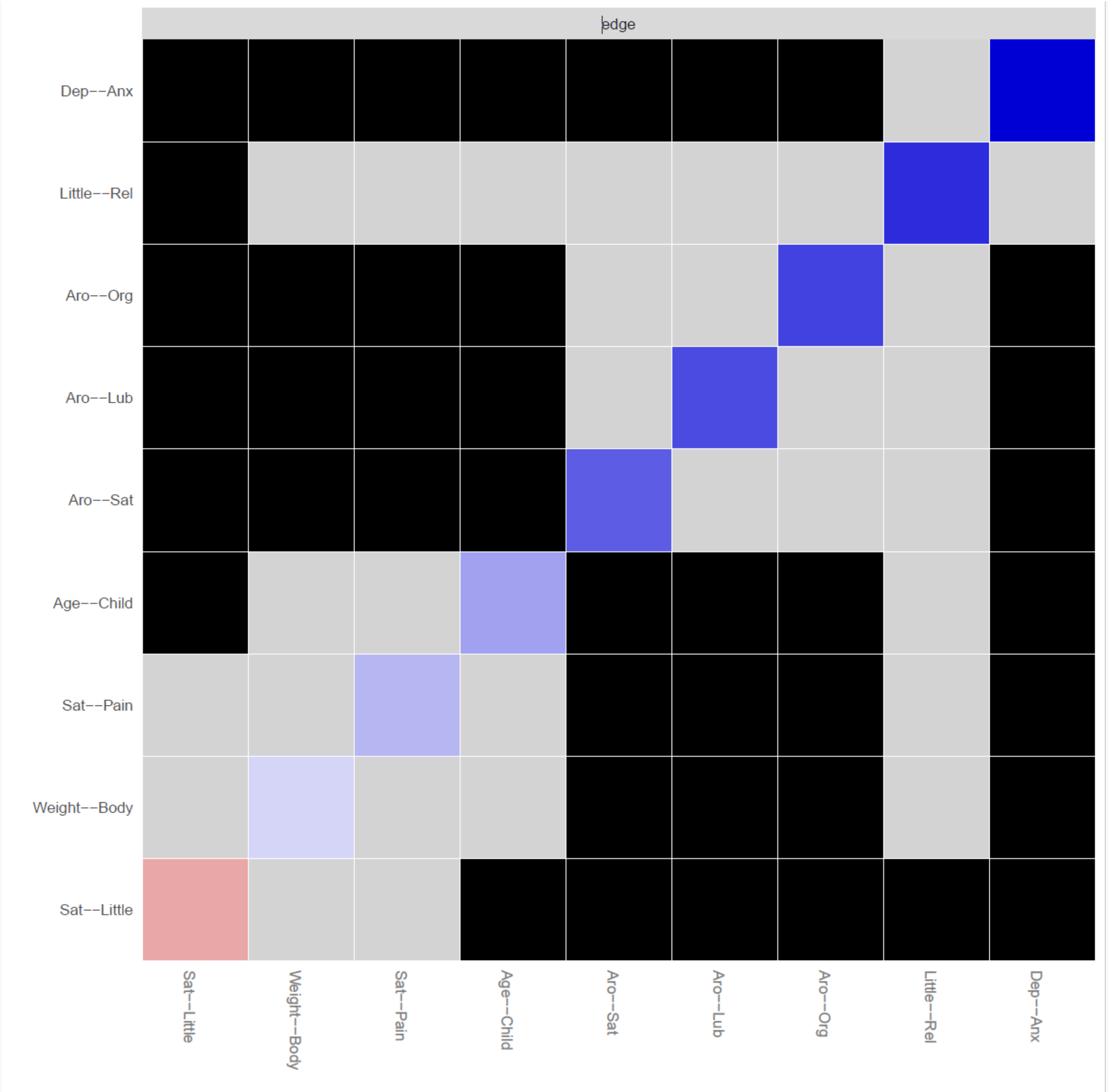
edge
Dep−−Anx
Little−−Rel
Aro−−Org
Aro−−Lub
Aro−−Sat
Age−−Child
Sat−−Pain
Weight−−Body
Sat−−Little
Sat−−Little
Weight−−Body
Sat−−Pain
Age−−Child
Aro−−Sat
Aro−−Lub
Aro−−Org
Little−−Rel
Dep−−Anx

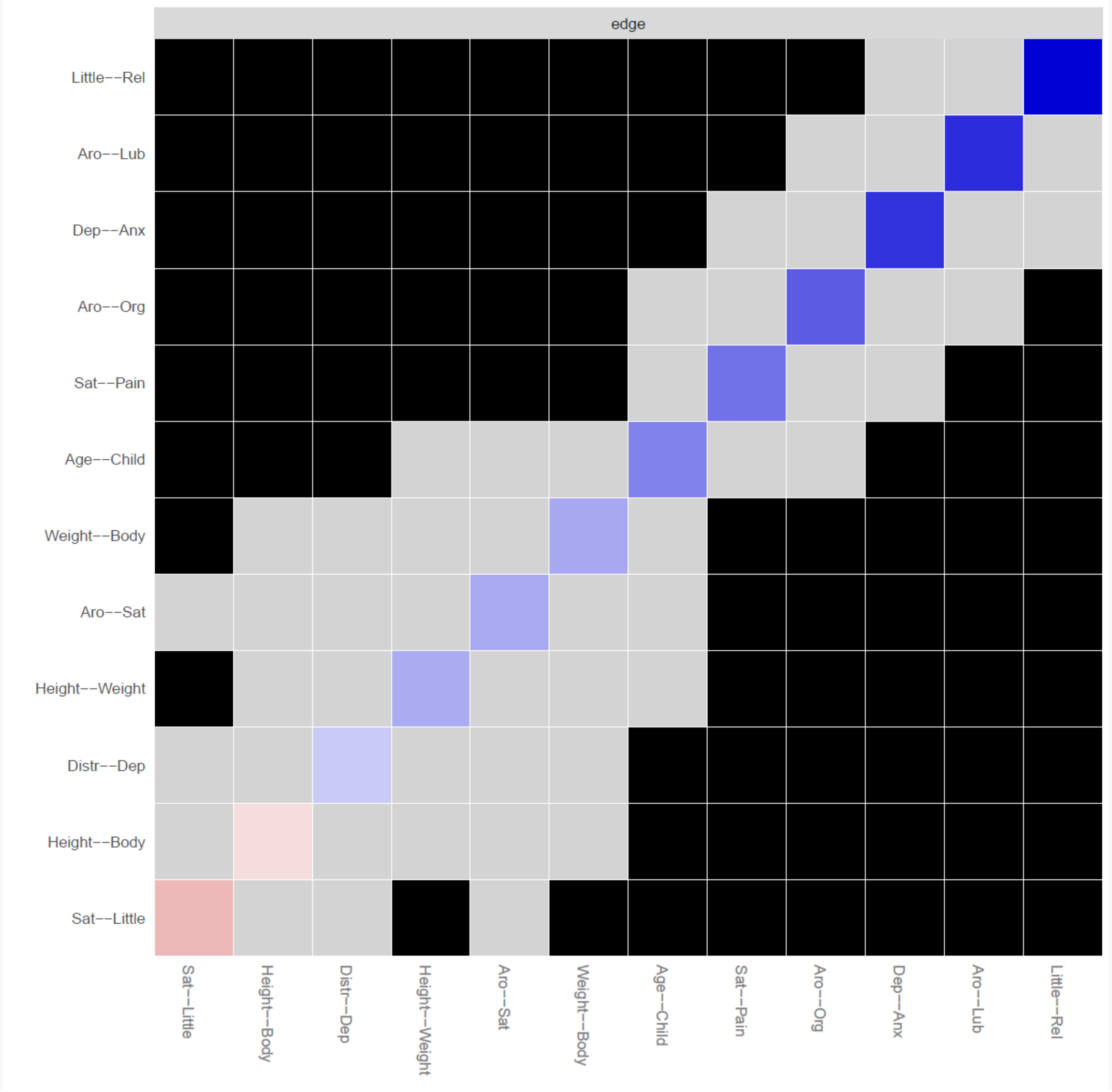
edge
Little--Rel
Aro--Lub
Dep--Anx
Aro--Org
Sat--Pain
Age--Child
Weight--Body
Aro--Sat
Height--Weight
Distr--Dep
Height--Body
Sat--Little

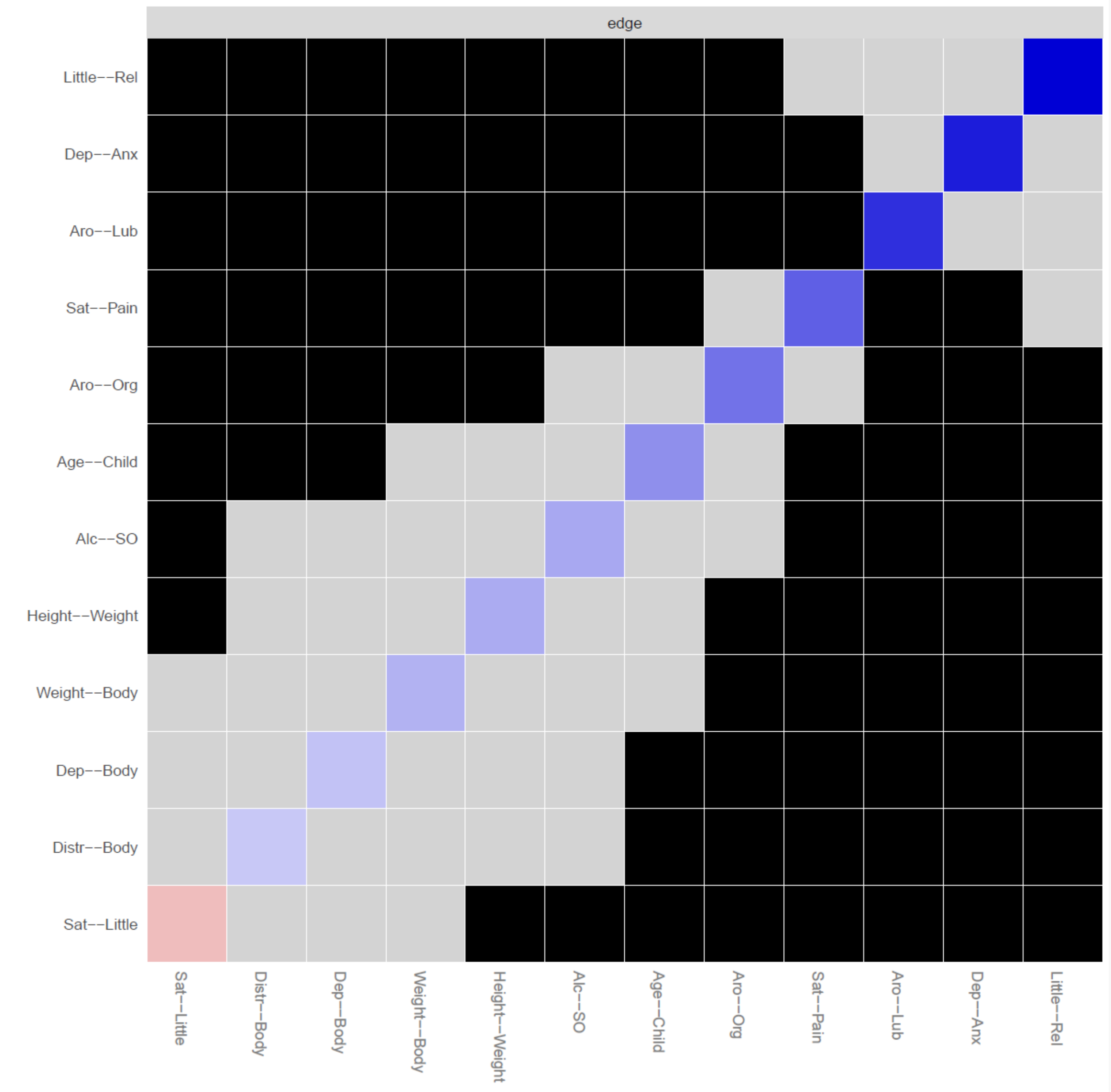


```
pdf("StrengthSignificanceReplication.pdf")
plot(fit_DecreaseBootNEW2, "strength", plot = "difference", order = "sample")
## Expected significance level given number of bootstrap samples is approximately: 0.05
plot(fit_StableBootNEW2, "strength", plot = "difference", order = "sample")
## Expected significance level given number of bootstrap samples is approximately: 0.05
plot(fit_IncreaseBootNEW2, "strength", plot = "difference", order = "sample")
## Expected significance level given number of bootstrap samples is approximately: 0.05
dev.off()
## png
##   2
```

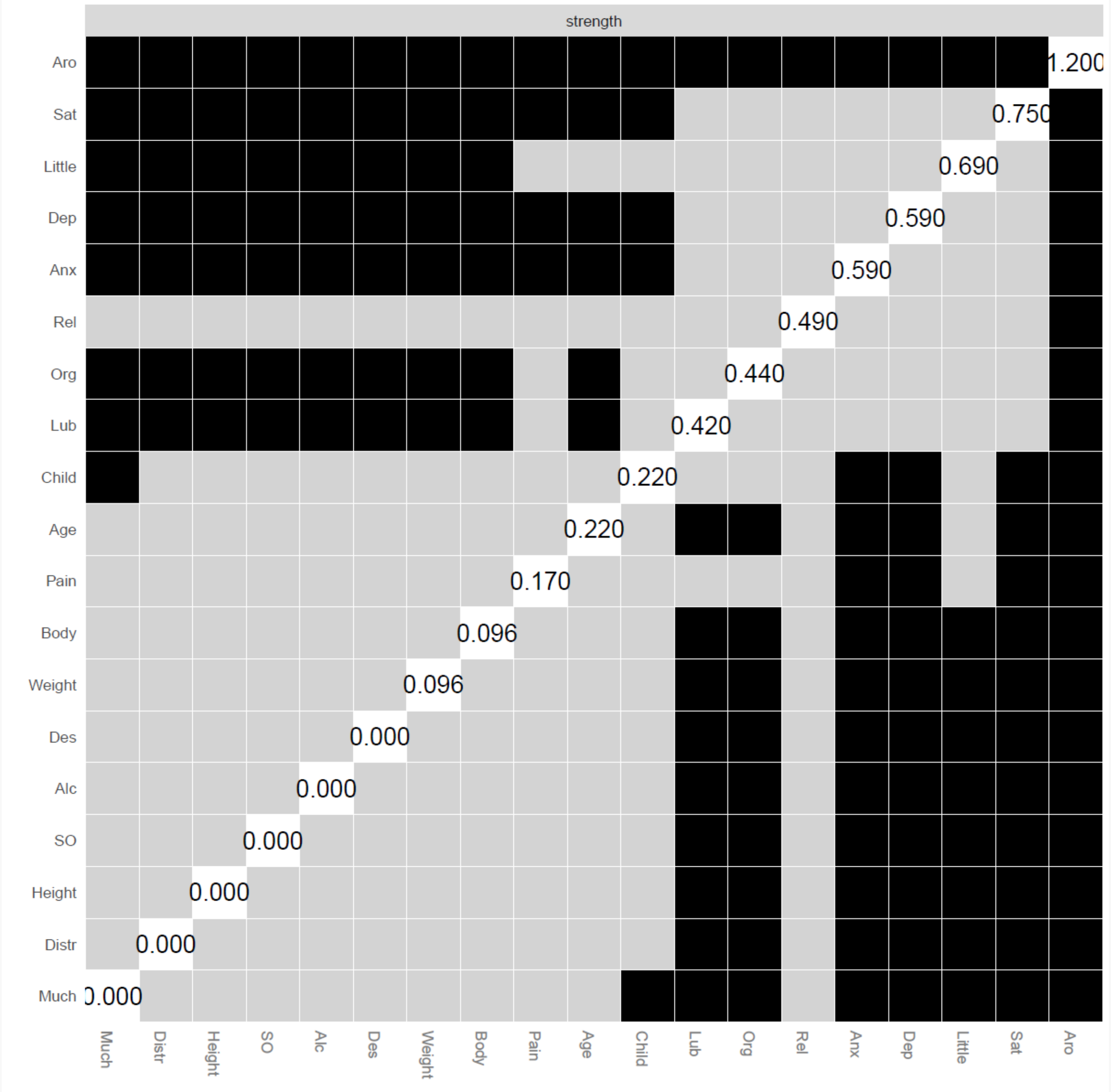

strength
Aro
Sat
Little
Dep
Anx
Rel
Org
Lub
Child
Age
Pain
Body
Weight
Des
Alc
SO
Height
Distr
Much
1.200
0.750
0.690
0.590
0.590
0.490
0.440
0.420
0.220
0.220
0.170
0.096
0.096
0.000
0.000
0.000
0.000
0.000
0.000

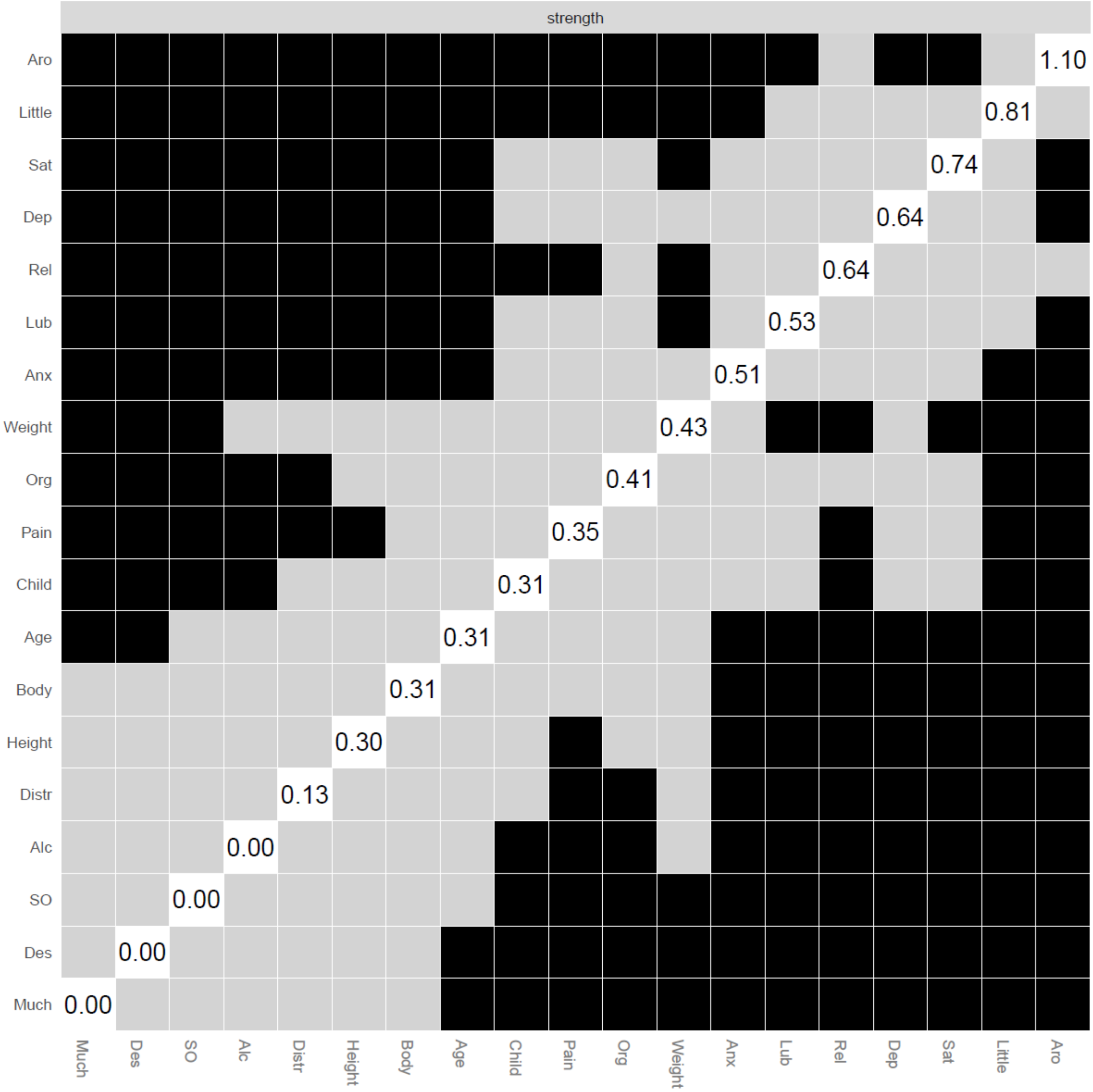

strength
Aro
Little
Sat
Dep
Rel
Lub
Anx
Weight
Org
Pain
Child
Age
Body
Height
Distr
Alc
SO
Des
Much
1.10
0.81
0.74
0.64
0.64
0.53
0.51
0.43
0.41
0.35
0.31
0.31
0.31
0.30
0.13
0.00
0.00
0.00
0.00

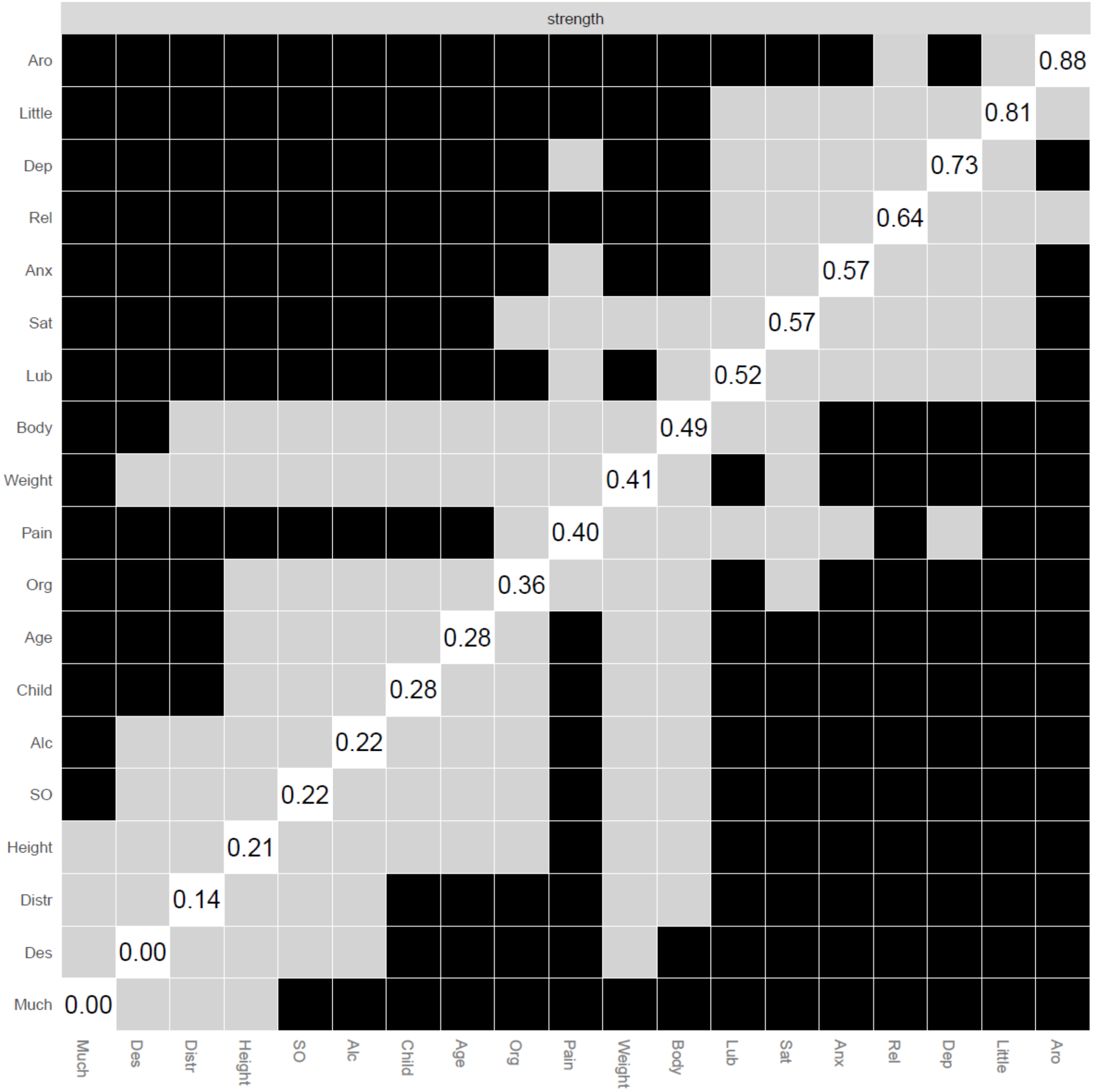

16. Replication – Network Comparison

```
###########################################################################################
# NCT

library(NetworkComparisonTest)

NCTDecStabNEW <- NCT(DecreaseNEWRS, StableNEWRS, gamma = 0.5, it = 1000, binary.data=FALSE, paired=FALSE, weighted=TRUE, progressbar=TRUE)

NCTDecStabNEW$nwinv.pval
## [1] 0.128

NCTDecStabNEW$glstrinv.real
## [1] 0.419842
NCTDecStabNEW$glstrinv.pval
## [1] 0.577
```

```
NCTIncStabNEW <- NCT(StableNEWRS, IncreaseNEWRS, gamma = 0.5, it = 1000, binary.data=FALSE, pai
red=FALSE, weighted=TRUE, progressbar=TRUE)

NCTIncStabNEW$nwinv.pval
## [1] 0.55

NCTIncStabNEW$glstrinv.real
## [1] 0.4022591
NCTIncStabNEW$glstrinv.pval
## [1] 0.556

NCTDecIncNEW <- NCT(DecreaseNEWRS, IncreaseNEWRS, gamma = 0.5, it = 1000, binary.data=FALSE, pa
ired=FALSE, weighted=TRUE, progressbar=TRUE)

NCTDecIncNEW$nwinv.pval
## [1] 0.003

NCTDecIncNEW$glstrinv.real
## [1] 0.01758292
NCTDecIncNEW$glstrinv.pval
## [1] 0.974
# no significant differences, but presumably because of power
###########################################################################################
mean(as.matrix(clustcoef_auto(qDecreaseNEW)))
## [1] 0.08534148
mean(as.matrix(clustcoef_auto(qStableNEW)))
## [1] 0.07164526
mean(as.matrix(clustcoef_auto(qIncreaseNEW)))
## [1] 0.08100884
###########################################################################################
```

17. Replication – Network Communities

```
# CLUSTER ANALYSIS
#### DECREASE

library(igraph)

NetworkDecreaseIgraphNEW <- as.igraph(qDecreaseNEW)

noClusterDecreaseNEW <- 0
for (i in 1:1000){
  communityWTDecreaseNEW <- cluster_walktrap(NetworkDecreaseIgraphNEW,
                                          weights = E(NetworkDecreaseIgraphNEW)$weight,
                                          steps = i,
                                          merges = TRUE,
                                          modularity = TRUE,
                                          membership = TRUE)
  noClusterDecreaseNEW[i] <- length(communityWTDecreaseNEW)
}

plot(noClusterDecreaseNEW)
```

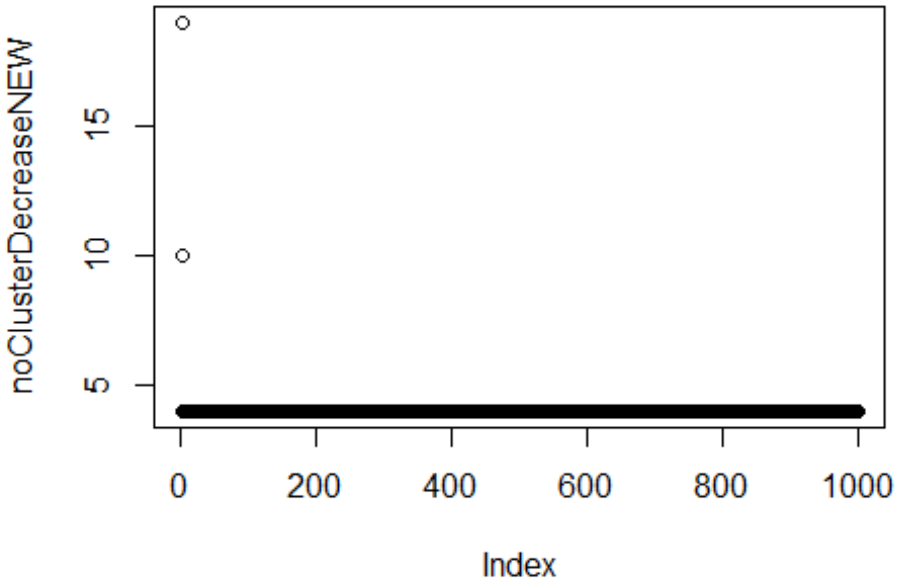


```
median(noClusterDecreaseNEW)
## [1] 4
mean(noClusterDecreaseNEW)
## [1] 4.021

communityWTPlotDecreaseNEW <- cluster_walktrap(NetworkDecreaseIgraphNEW,
                                               weights = E(NetworkDecreaseIgraphNEW)$weight,
                                               steps = 200,
                                               merges = TRUE,
                                               modularity = TRUE,
                                               membership = TRUE)

plot(communityWTPlotDecreaseNEW, NetworkDecreaseIgraphNEW, layout = qDecreaseNEW$layout)
```

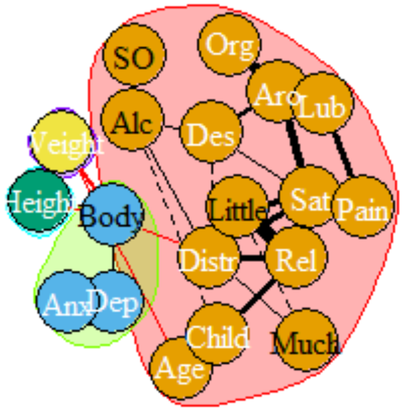


```
# For comparison’s sake we copied the first graph here below:
```

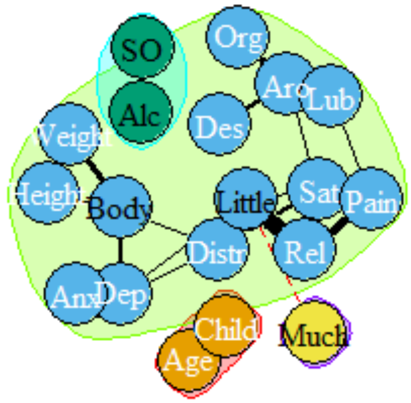


```
# STABLE
NetworkStableIgraphNEW <- as.igraph(qStableNEW)

noClusterStableNEW <- 0
for (i in 1:1000){
  communityWTStableNEW <- cluster_walktrap(NetworkStableIgraphNEW,
                                           weights = E(NetworkStableIgraphNEW)$weight,
                                           steps = i,
                                           merges = TRUE,
                                           modularity = TRUE,
                                           membership = TRUE)
  noClusterStableNEW[i] <- length(communityWTStableNEW)
}

plot(noClusterStableNEW[0:1000])
```

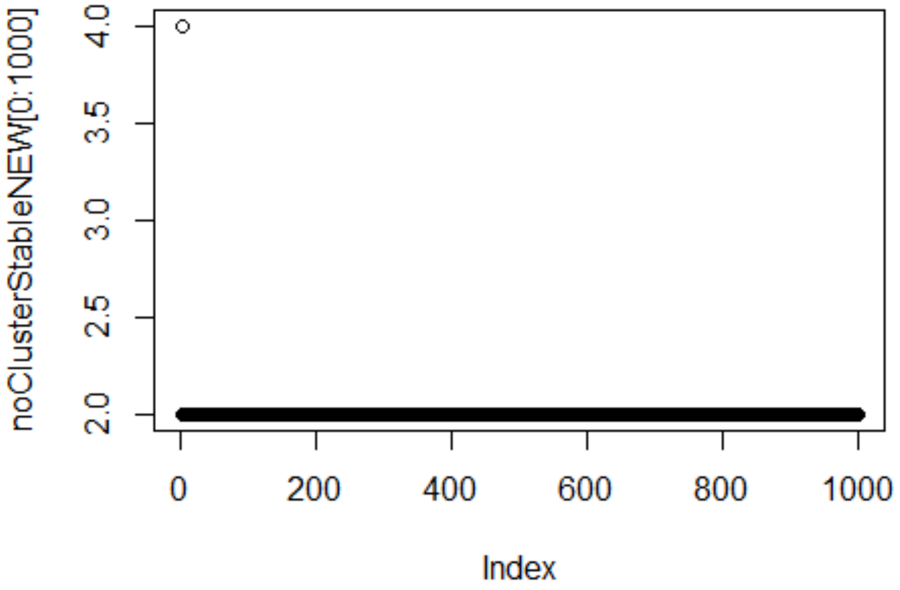


```
median(noClusterStableNEW[0:1000])
## [1] 2
mean(noClusterStableNEW[0:1000])
```

```
## [1] 2.002

communityWTPlotStableNEW <- cluster_walktrap(NetworkStableIgraphNEW,
                                             weights = E(NetworkStableIgraphNEW)$weight,
                                             steps = 200,
                                             merges = TRUE,
                                             modularity = TRUE,
                                             membership = TRUE)

plot(communityWTPlotStableNEW, NetworkStableIgraphNEW, layout = qStableNEW$layout)
```

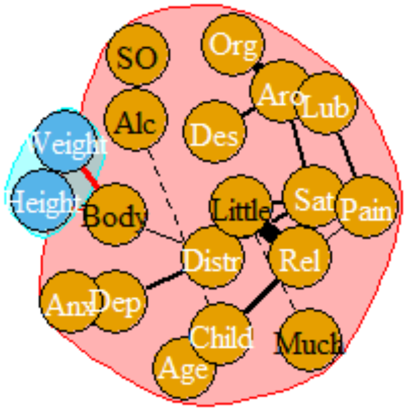


```
# For comparison's sake we copied the first graph here below:
```

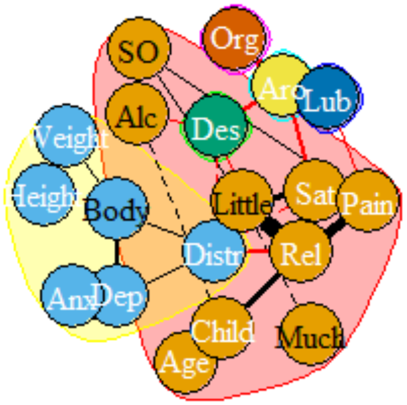


```
#### Increase
NetworkIncreaseIgraphNEW <- as.igraph(qIncreaseNEW)

noClusterIncreaseNEW <- 0
for (i in 1:1000){
  communityWTIncreaseNEW <- cluster_walktrap(NetworkIncreaseIgraphNEW,
                                             weights = E(NetworkIncreaseIgraphNEW)$weight,
                                             steps = i,
                                             merges = TRUE,
                                             modularity = TRUE,
                                             membership = TRUE)
  noClusterIncreaseNEW[i] <- length(communityWTIncreaseNEW)
}

plot(noClusterIncreaseNEW[0:1000])
```

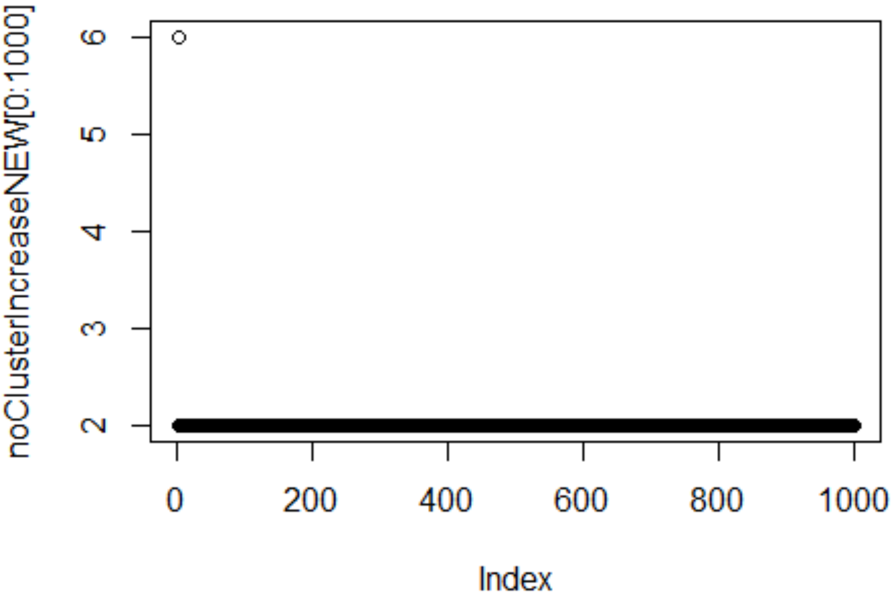


```
median(noClusterIncreaseNEW[0:1000])
## [1] 2
mean(noClusterIncreaseNEW[0:1000])
## [1] 2.004

communityWTPlotIncreaseNEW <- cluster_walktrap(NetworkIncreaseIgraphNEW,
                                               weights = E(NetworkIncreaseIgraphNEW)$weight,
                                               steps = 200,
                                               merges = TRUE,
                                               modularity = TRUE,
                                               membership = TRUE)

plot(communityWTPlotIncreaseNEW, NetworkIncreaseIgraphNEW, layout = qIncreaseNEW$layout)
```

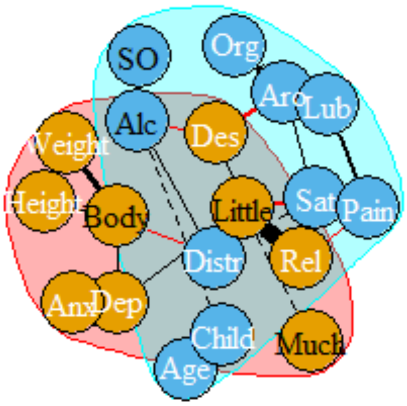


```
# For comparison's sake we copied the first graph here below:
```

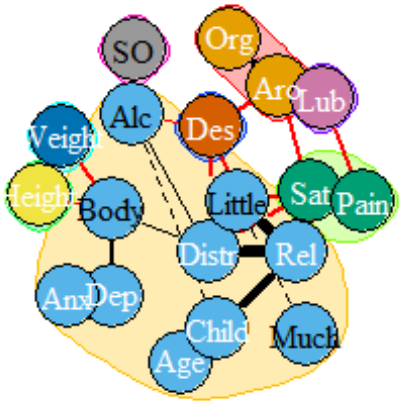


```r
pdf("WalktrapClusterReplication.pdf")
plot(communityWTPlotDecreaseNEW, NetworkDecreaseIgraphNEW, layout = qDecreaseNEW$layout)
plot(communityWTPlotStableNEW, NetworkStableIgraphNEW, layout = qStableNEW$layout)
plot(communityWTPlotIncreaseNEW, NetworkIncreaseIgraphNEW, layout = qIncreaseNEW$layout)
dev.off()
## png
##   2
```
